# HPV transmission and optimal control of cervical cancer in China

**DOI:** 10.1038/s41598-025-05514-y

**Published:** 2025-07-01

**Authors:** Huarong Ren, Rui Xu, Juan Zhang

**Affiliations:** 1 Complex Systems Research Center, Shanxi University, Taiyuan, 030006 Shanxi People’s Republic of China; 2 Complex Systems and Data Science Key Laboratory of Ministry of Education, Taiyuan, 030006 Shanxi People’s Republic of China; 3 School of Mathematics and Statistics, Shanxi University, Taiyuan, 030006 Shanxi People’s Republic of China

**Keywords:** Cervical cancer, Human papillomavirus, Optimum control strategy, Dynamical model, Age structure, Sensitivity analysis, Diseases, Risk factors

## Abstract

Cervical cancer (CC), the fourth most common female malignancy globally, is predominantly caused by persistent infection with high-risk human papillomavirus (HPV) strains. China faces a particularly severe burden, exhibiting both the world’s second-highest CC prevalence and an alarming epidemiological trend characterized by steadily increasing incidence rates and younger age of onset. In response to the WHO’s global elimination strategy, China launched the Action Plan for Accelerated Elimination of Cervical Cancer (2023–2030). Aligned with national prevention and control objectives, considering two key interventions, that is widespread HPV vaccination and systematic CC screening, this study develops a novel two-sex, age-structured transmission model to characterize HPV transmission dynamics and CC progression, and implementation of intervention strategies. Through mathematical modeling analysis and simulation, we quantify age- and sex-specific risk profiles, predict long-term epidemiological trends. Using optimal control theory, we propose a supply-constrained vaccine allocation strategy for maximal population protection and a cost-effective screening implementation plan, which will provide evidence-based recommendations to support China’s national CC elimination goals.

## Introduction

Cervical cancer (CC) is the fourth most common cancer in women^1^. In 2018, the estimated age-standardised incidence of CC was 13.1 per 100,000 women globally^2^. Age-standardised incidence of CC varied widely among countries, with rates ranging from less than 2–75 per 100,000 women^2^. In 2022, there were estimated to be about 660,000 new cases and nearly 350,000 deaths globally^1^. Almost all CC cases (99%) are linked to infection with high-risk human papillomaviruses (HPV). HPV is an extremely common virus transmitted through sexual contact^1^. HPV16 and HPV18 are the most prevalent high-risk HPV types in CC, with HPV16 accounting for 55.2% of cases and HPV18 for 14.2%^3^. High-risk groups are those with multiple sexual partners, early age sexual debut, or immune deficiency^4^. CC may be asymptomatic in the early stages, but can cause vaginal bleeding and abnormal vaginal drainage as the disease progresses^4^.

In China, whether in rural or urban areas, the risk of CC is on the rise, and the age of onset of patients with CC tends to be younger^5^. In terms of total cases and total deaths, China is the second-largest country with the disease burden of CC in the world^6^. Since 2000, the incidence and mortality rates of CC in China have been increasing. In 2020, CC cases in China accounted for 18% of the world, CC deaths accounted for 17% of the world^7^. The incidence of CC among females aged 15–44 ranks third among female tumors^6^. In 2022, the number of new cases of CC in China was 151,000, and the incidence rate was 13.8 per 100,000, ranking fifth in the incidence of female cancer^5^. There were 56,000 deaths in that year, with a death rate of 4.5 per 100,000, ranking sixth in the death rate of female cancer^5^.

The primary prevention measures of CC encompass health education and HPV vaccination. The secondary prevention measures comprise CC screening and the early treatment of patients with cervical precancerous lesions. The tertiary prevention measure of CC is the treatment of patients with CC^8,9^. Existing HPV vaccines comprise bivalent vaccines for HPV16/18, quadrivalent vaccines for HPV6/11/16/18, and nonavalent vaccines for HPV6/11/16/18/31/33/45/52/58. All three vaccines are protective against HPV16/18. At present, the only HPV vaccine that has been developed and approved for marketing in China is the bivalent vaccine^10^. Therefore, this study focuses on investigating the transmission dynamics of HPV16 and HPV18 in human populations. Existing vaccines are available for women aged 9–45. Adolescent girls who have not experienced sexual debut will achieve the best preventive efficacy by receiving the HPV vaccine. Therefore, WHO recommends designating girls aged 9–14 as the primary targets for vaccination and ensuring high vaccination coverage among them. Females aged $\ge 15$ years or males are recommended as secondary target populations if this is affordable, cost-effective, and does not divert resources from effective CC screening^11^. Additionally, vaccination recommendations from the United States Centers for Disease Control and Prevention (CDC) are as follows^12^: (1) All adolescent boys and girls who are 11 or 12 years old can receive HPV vaccine. (2) Catch-up vaccine is recommended for males up to age 21 and for females up to age 26, if they did not get vaccinated when they were younger. Due to shortages of the HPV vaccine, in late 2019, WHO called for a suspension of HPV vaccination for adolescent boys until girls have been fully vaccinated^13^.

Since the introduction of HPV vaccines in China in 2017, vaccination coverage has demonstrated a consistent annual increase^14^. However, immunization rates among young women remain substantially below both national targets and global averages^14^. As of 2022, only 10.15% of the target population had received the first vaccine dose, with merely 6.21% completing the full three-dose regimen^15^. These vaccination levels fall far short of the threshold required to achieve herd immunity against CC. Multiple systemic factors that contribute to this challenge are persistent vaccine supply shortages, insufficient public health education initiatives, limited vaccination awareness among target populations, and prohibitive costs creating financial barriers to access vaccination^14^. In light of WHO and CDC vaccination guidelines, coupled with China’s current immunization landscape, several critical policy questions emerge:

- Should vaccination programs be extended to the male population?
- How to address the immunization needs of unvaccinated individuals aged $\ge 15$ years?
- What constitutes the optimal vaccine allocation strategy under supply constraints?
- Which cost-effective intervention framework can most efficiently achieve elimination targets?

At present, the impact of HPV vaccines on HPV transmission has been studied in some works through mathematical modeling. Elbasha et al. used an age-structured HPV transmission model to compare the cost-effectiveness of different vaccination strategies in the United States^16–18^. The study found that incorporating males and adolescent boys into the vaccination plan is the most effective strategy^16–18^. Xia et al. formulated an age-structured HPV transmission model, and evaluated different strategies for female vaccination and CC screening based on public health effect and cost-effectiveness, aiming to find the best path to eliminate CC in China^19^. Gao et al. used a two-sex transmission model that only depicts the vaccination of 9–14 year olds to evaluate HPV vaccination strategies, and performed numerical simulations using data from Guangxi Province in China as an example^20^. They found that when the number of vaccines is fixed, priority should be given to vaccinating genders with lower recruit rate rather than evenly distributing vaccines^20^. The above works only conducted numerical simulations on some alternative vaccination and screening strategies, and then formulated some evaluation indicators to find better strategies. However, the above works did not find the optimal time-varying control strategy through optimal control theory^21,22^.

Some scholars have employed optimal control theory to address challenges in disease control and prevention. Madhu introduced an optimal control problem to estimate the optimal vaccination strategy needed to control HPV within 25 years^23^. Saldaña et al. formulated a deterministic mathematical model that includes the most important epidemiological features of HPV infection and related cancers, and also formulated an optimal control problem^24^. They found that it is possible to eradicate HPV-related cancers even if adolescent boys and adult males are not vaccinated^24^. And the optimal vaccine deployment is to allocate as many vaccines as possible during the initial stage of the epidemic, and then gradually reduce the vaccination rate after a period of time^24^. The above two works did not perform data fitting on the number of CC cases and deaths to confirm the rationality of the model. Instead, they assumed the values of some parameters and then carried out the numerical simulations of the optimal control. In addition, they did not consider the control strategy of CC screening, and did not take into account the real cost of vaccines, screening and treatment. In the optimal control problems of other diseases, the cost of the real control strategy was considered, but the authors did not take into account the terminal control target^25,26^. The terminal control targets refer to the targets that the state variables need to achieve under control measures at the terminal time *T*. For example, the number of infected individuals at the terminal time *T* is required to be 0. In this paper, for the optimal control problem, we construct an objective function based on the terminal control target and related cost data.

The framework of this paper is organized as follows. Firstly, based on the transmission dynamics of HPV, the pathogenesis of CC, and the prevention and control measures, a two-sex transmission model with age structure is formulated. Threshold dynamics of the model is analyzed. Secondly, the number of CC cases and deaths related to HPV16/18 in China is fitted to obtain values and 95% confidence intervals of the parameters to be estimated. Thirdly, the optimization problem of vaccine allocation under limited vaccine supply is formulated, and the optimal vaccine allocation is obtained through numerical simulations. Fourthly, an optimal control problem and several evaluation indicators are proposed according to the control cost and the terminal control targets for variables. The existence of an optimal control is proved, and the necessary conditions and characterization of optimal control are given. Control strategies with minimum control costs for vaccination and CC screening in China are studied through numerical simulations.

Based on the cases and deaths of CC related to HPV16 and HPV18 from 2006 to 2016 in China, this study develops a dynamical model to analyze the influence of age and gender on the transmission dynamics of HPV16/18 in China, and to provide critical scientific evidence for the formulation of related prevention and control strategies. This study establishes optimal vaccination priority rankings for vaccination populations (adolescent girls and boys aged 14 years, females and males aged 15–44 years) to minimize HPV16/18 transmission under limited vaccine supplies, and conducts a comparative analysis of basic reproduction number across distinct prioritization strategies. Additionally, this study simulates the cost-effective CC prevention and control program that minimizes cost for vaccination, screening and treatment, aiming to achieve the terminal control objective of CC elimination.

## Materials and methods

### Data

In China, the cumulative cases and cumulative deaths of CC related to HPV16 and HPV18 from 2006 to 2016 and the data sources are shown in Appendix 4.

### Model formulation

**Fig. 1:**
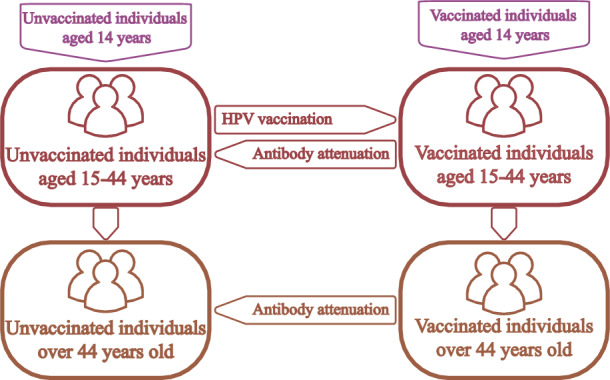
Classification and relationship of populations according to vaccination strategies.

**Fig. 2:**
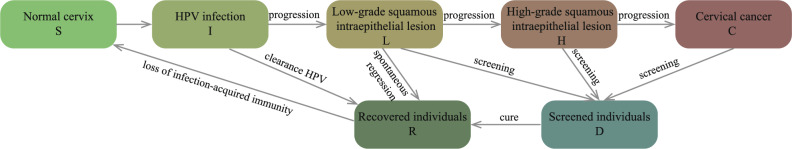
The natural course of HPV infection in the cervix.

In this section, we develop a compartment model based on the transmission process of HPV. Sexual transmission is the most important mode of transmission for HPV. Therefore, our study focuses on the population aged 15 years and above, and the population is divided into two groups: males and females, which are denoted by subscripts *m* and *f*, respectively. At time *t*, the total population aged 15 and above is denoted by *N*(*t*), the total male population and the total female population are recorded as $N_m(t)$ and $N_f(t)$, respectively. In addition, the male and female populations are stratified into two age subgroups based on HPV vaccination eligibility criteria: 15–44 years, $\ge 45$ years. These age strata are subsequently denoted by subscripts 1 and 2, respectively. At time *t*, the total female and male populations in the first (second) age group are denoted as $N_{f1}(t)$ ($N_{f2}(t)$) and $N_{m1}(t)$ ($N_{m2}(t)$) respectively. Finally, combining with the natural course of HPV infection in the cervix^11^ (see Fig. 2) and the status of vaccination (see Fig. 1), females are classified into 24 compartments (see Table 1). Males are simply classified into 10 compartments (see Table 1). According to the classification of the population, the following equations hold:

$$\begin{aligned} N_{f}&=N_{f1}+N_{f2},\\ N_{m}&=N_{m1}+N_{m2},\\ N_{f1}&=S^{u}_{f1}+I^{u}_{f1}+L^{u}_{f1}+H^{u}_{f1}+C^{u}_{f1}+S^{v}_{f1} +I^{v}_{f1}+L^{v}_{f1}+H^{v}_{f1}+C^{v}_{f1}+D_{f1}+R_{f1},\\ N_{f2}&=S^{u}_{f2}+I^{u}_{f2}+L^{u}_{f2}+H^{u}_{f2}+C^{u}_{f2}+S^{v}_{f2} +I^{v}_{f2}+L^{v}_{f2}+H^{v}_{f2}+C^{v}_{f2}+D_{f2}+R_{f2},\\ N_{m1}&=S^{u}_{m1}+I^{u}_{m1}+S^{v}_{m1}+I^{v}_{m1}+R_{m1},\\ N_{m2}&=S^{u}_{m2}+I^{u}_{m2}+S^{v}_{m2}+I^{v}_{m2}+R_{m2}. \end{aligned}$$

**Table 1:** Description of state variables in model (1)–(4).

Variables	Description
$S_{fi}^{u}(S_{fi}^{v})$	Unvaccinated (vaccinated) susceptible females in the *i-*th age group ($i\in \{1,2\}$)
$I_{fi}^{u}(I_{fi}^{v})$	Unvaccinated (vaccinated) infected females in the *i-*th age group
$L_{fi}^{u}(L_{fi}^{v})$	Unvaccinated (vaccinated) females in the *i-*th age group with Low-grade squamous intraepithelial lesion (LSIL)
$H_{fi}^{u}(H_{fi}^{v})$	Unvaccinated (vaccinated) females in the *i-*th age group with High-grade squamous intraepithelial lesion (HSIL)
$C_{fi}^{u}(C_{fi}^{v})$	Unvaccinated (vaccinated) females in the *i-*th age group with CC
$D_{fi}$	HPV-infected females and female patients with LSIL, HSIL and CC screened out in the *i-*th age group
$R_{fi}$	Female recoveries in the *i-*th age group
$S_{mi}^{u}(S_{mi}^{v})$	Unvaccinated (vaccinated) susceptible males in the *i-*th age group
$I_{mi}^{u}(I_{mi}^{v})$	Unvaccinated (vaccinated) infected males in the *i-*th age group
$R_{mi}$	Male recoverers in the *i-*th age group

**Fig. 3:**
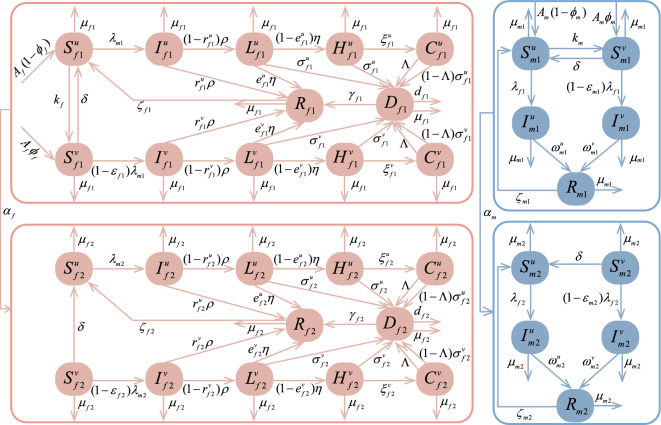
Transfer diagram of model (1)–(4).

Next, with the help of transfer diagram (see Fig. 3) and parameter definition (see Table 2), the process of population transfer among various compartments is described in detail. The first age group is used as an example for specific illustration, and the second age group is similar. In unit time, as the age increases, the number of unvaccinated people entering $S^{u}_{f1}$ ($S^{u}_{m1}$) is $A_{f}(1-\phi _{f})$ ($A_{m}(1-\phi _{m})$), and the number of vaccinated people entering $S^{v}_{f1}$ ($S^{v}_{m1}$) is $A_{f}\phi _{f}$ ($A_{m}\phi _{m}$). In unit time, the number of people removed from $S^{u}_{f1}$ due to natural death is $\mu _{f1}S^{u}_{f1}$, and other compartments are similar. In unit time, the number of people who are transferred from $S^{u}_{f1}$ ($S^{u}_{m1}$) to $S^{v}_{f1}$ ($S^{v}_{m1}$) due to vaccination is $kS^{u}_{f1}$ ($kS^{u}_{m1}$), and the number of people transferred from $S^{v}_{f1}$ ($S^{v}_{m1}$) to $S^{u}_{f1}$ ($S^{u}_{m1}$) due to antibody attenuation is $\delta S^{v}_{f1}$ ($\delta S^{v}_{m1}$). After effective contact with infected males (i.e. $I_{mi}^{u}$ and $I_{mi}^{v}$, where $i\in \{1,2\}$), individuals in $S^{u}_{f1}$ and $S^{u}_{f2}$ are infected with HPV at rates $\lambda _{m1}$ and $\lambda _{m2}$, where

$$\begin{aligned} \lambda _{m1}&=\frac{\beta _{m}c_{m11}(I_{m1}^{u}+I_{m1}^{v}) +\beta _{m}c_{m21}(I_{m2}^{u}+I_{m2}^{v})}{N_{f1}},\\ \lambda _{m2}&=\frac{\beta _{m}c_{m12}(I_{m1}^{u}+I_{m1}^{v}) +\beta _{m}c_{m22}(I_{m2}^{u}+I_{m2}^{v})}{N_{f2}}. \end{aligned}$$

Likewise, after effective contact with infected females (i.e. $I_{fi}^{h}$, $L_{fi}^{h}$, $H_{fi}^{h}$ and $C_{fi}^{h}$, where $i\in \{1,2\}$ and $h\in \{u,v\}$), individuals in $S^{u}_{m1}$ and $S^{u}_{m2}$ are infected with HPV at rates $\lambda _{f1}$ and $\lambda _{f2}$, where

$$\begin{aligned} \lambda _{f1}&=\frac{\beta _{f}c_{f11}\left( \sum _{h\in \{u,v\}}I_{f1}^{h} +L_{f1}^{h}+H_{f1}^{h}+C_{f1}^{h}\right) }{N_{m1}} +\frac{\beta _{f}c_{f21}\left( \sum _{h\in \{u,v\}}I_{f2}^{h}+L_{f2}^{h} +H_{f2}^{h}+C_{f2}^{h}\right) }{N_{m1}},\\ \lambda _{f2}&=\frac{\beta _{f}c_{f12}\left( \sum _{h\in \{u,v\}}I_{f1}^{h} +L_{f1}^{h}+H_{f1}^{h}+C_{f1}^{h}\right) }{N_{m2}} +\frac{\beta _{f}c_{f22}\left( \sum _{h\in \{u,v\}}I_{f2}^{h}+L_{f2}^{h} +H_{f2}^{h}+C_{f2}^{h}\right) }{N_{m2}}. \end{aligned}$$

Due to the reduced susceptibility after vaccination, individuals in $S^{v}_{f1}$, $S^{v}_{f2}$, $S^{v}_{m1}$ and $S^{v}_{m2}$ are infected at rates $(1-\varepsilon _{f1})\lambda _{m1}$, $(1-\varepsilon _{f2})\lambda _{m2}$, $(1-\varepsilon _{m1})\lambda _{f1}$ and $(1-\varepsilon _{m2})\lambda _{f2}$, respectively.

In unit time, the number of people who move from $I^{u}_{f1}$ ($I^{v}_{f1}$) to the $R_{f1}$ due to the clearing of virus is $r_{f1}^{u}\rho I^{u}_{f1}$ ($r_{f1}^{v}\rho I^{v}_{f1}$), and the number of people who move from $I^{u}_{f1}$ ($I^{v}_{f1}$) to the $L^{u}_{f1}$ ($L^{v}_{f1}$) due to persistent infection is $(1-r_{f1}^{u})\rho I^{u}_{f1}$ ($(1-r_{f1}^{v})\rho I^{v}_{f1}$). For individuals in $L^{u}_{f1}$ ($L^{v}_{f1}$), the natural recovery rate is $e_{f1}^{u}\eta L^{u}_{f1}$ ($e_{f1}^{v}\eta L^{v}_{f1}$), the screening rate is $\sigma ^{u}_{f1}L^{u}_{f1}$ ($\sigma ^{v}_{f1}L^{v}_{f1}$), and the rate of transition to HSIL is $(1-e_{f1}^{u})\eta L^{u}_{f1}$ ($(1-e_{f1}^{v})\eta L^{v}_{f1}$). HSIL has the potential to become cancerous and need to be screened and treated in a timely manner^27^. For individuals in $H^{u}_{f1}$ ($H^{v}_{f1}$), the screening rate is $\sigma ^{u}_{f1}H^{u}_{f1}$ ($\sigma ^{v}_{f1}H^{v}_{f1}$), and the rate of canceration is $\xi ^{u}_{f1} H^{u}_{f1}$ ($\xi ^{v}_{f1} H^{v}_{f1}$). For the individuals in $C^{u}_{f1}$ ($C^{v}_{f1}$), the number of diagnosed patients who spontaneously go to the hospital for treatment due to symptoms within a unit time is $\Lambda C^{u}_{f1}$ ($\Lambda C^{v}_{f1}$), and the number of asymptomatic CC patients diagnosed due to CC screening is $(1-\Lambda )\sigma ^{u}_{f1}C^{u}_{f1}$ ($(1-\Lambda )\sigma ^{v}_{f1}C^{v}_{f1}$). In unit time, individuals in $D_{f1}$ die from illness at rate $d_{f1}D_{f1}$ and recover at rate $\gamma _{f1}D_{f1}$. In unit time, the number of individuals in $I^{u}_{m1}$ ($I^{v}_{m1}$) is reduced by $\omega ^{u}_{m1} I^{u}_{m1}$ ($\omega ^{v}_{m1} I^{v}_{m1}$). Due to the loss of infection-acquired immunity, the number of people entering $S^{u}_{f1}$ ($S^{u}_{m1}$) from $R_{f1}$ ($R_{m1}$) per unit time is $\zeta _{f1}R_{f1}$ ($\zeta _{m1}R_{m1}$). In unit time, with the growth of age, the numbers of females and males who transfer from the first age group to the second age group are $\alpha _{f}N_{f1}=\alpha _{f}\left( S^{u}_{f1}+I^{u}_{f1}+L^{u}_{f1}+H^{u}_{f1}+C^{u}_{f1} +S^{v}_{f1}+I^{v}_{f1}+L^{v}_{f1}+H^{v}_{f1}+C^{v}_{f1}+D_{f1}+R_{f1}\right)$ and $\alpha _{m}N_{m1}=\alpha _{m}\left( S^{u}_{m1}+I^{u}_{m1}+S^{v}_{m1}+I^{v}_{m1}+R_{m1}\right)$ respectively.

To assess the effect of CC prevention measures on eliminating CC in China, we construct the following model:

1 $$\begin{aligned} \frac{\textrm{d}S^{u}_{f1}}{dt}&=A_{f}(1-\phi _{f})-\lambda _{m1}S^{u}_{f1}-k_{f}S_{f1}^{u}+\delta S^{v}_{f1}-\mu _{f1} S^{u}_{f1}-\alpha _{f}S^{u}_{f1}+\zeta _{f1}R_{f1},\nonumber \\ \frac{\textrm{d}I^{u}_{f1}}{dt}&=\lambda _{m1}S^{u}_{f1}-\rho I^{u}_{f1}-\mu _{f1} I^{u}_{f1}-\alpha _{f}I^{u}_{f1},\nonumber \\ \frac{\textrm{d}L^{u}_{f1}}{dt}&=(1-r_{f1}^{u})\rho I^{u}_{f1}-\eta L^{u}_{f1}-\sigma _{f1}^{u}L^{u}_{f1}-\mu _{f1} L^{u}_{f1}-\alpha _{f}L^{u}_{f1},\nonumber \\ \frac{\textrm{d}H^{u}_{f1}}{dt}&=(1-e_{f1}^{u})\eta L^{u}_{f1}-\xi ^{u}_{f1} H^{u}_{f1}-\sigma ^{u}_{f1}H^{u}_{f1}-\mu _{f1}H^{u}_{f1}-\alpha _{f}H^{u}_{f1},\nonumber \\ \frac{\textrm{d}C^{u}_{f1}}{dt}&=\xi ^{u}_{f1} H^{u}_{f1}-\Lambda C^{u}_{f1}-(1-\Lambda )\sigma ^{u}_{f1}C^{u}_{f1}-\mu _{f1}C^{u}_{f1}-\alpha _{f}C^{u}_{f1},\nonumber \\ \frac{\textrm{d}S^{v}_{f1}}{dt}&=A_{f}\phi _{f}-(1-\varepsilon _{f1})\lambda _{m1}S^{v}_{f1}+k_{f}S_{f1}^{u}-\delta S^{v}_{f1}-\mu _{f1} S^{v}_{f1}-\alpha _{f}S^{v}_{f1},\nonumber \\ \frac{\textrm{d}I^{v}_{f1}}{dt}&=(1-\varepsilon _{f1})\lambda _{m1}S^{v}_{f1}-\rho I^{v}_{f1}-\mu _{f1} I^{v}_{f1}-\alpha _{f}I^{v}_{f1},\nonumber \\ \frac{\textrm{d}L^{v}_{f1}}{dt}&=(1-r_{f1}^{v})\rho I^{v}_{f1}-\eta L^{v}_{f1}-\sigma _{f1}^{v}L^{v}_{f1}-\mu _{f1} L^{v}_{f1}-\alpha _{f}L^{v}_{f1},\nonumber \\ \frac{\textrm{d}H^{v}_{f1}}{dt}&=(1-e_{f1}^{v})\eta L^{v}_{f1}-\xi ^{v}_{f1} H^{v}_{f1}-\sigma ^{v}_{f1}H^{v}_{f1}-\mu _{f1}H^{v}_{f1}-\alpha _{f}H^{v}_{f1},\nonumber \\ \frac{\textrm{d}C^{v}_{f1}}{dt}&=\xi ^{v}_{f1} H^{v}_{f1}-\Lambda C^{v}_{f1}-(1-\Lambda ) \sigma ^{v}_{f1}C^{v}_{f1}-\mu _{f1}C^{v}_{f1}-\alpha _{f}C^{v}_{f1},\nonumber \\ \frac{\textrm{d}D_{f1}}{dt}&=\sigma ^{u}_{f1}(L^{u}_{f1}+H^{u}_{f1}) +[\Lambda +(1-\Lambda )\sigma ^{u}_{f1}]C^{u}_{f1}+\sigma ^{v}_{f1}(L^{v}_{f1}+H^{v}_{f1})\nonumber \\&\quad +[\Lambda +(1-\Lambda )\sigma ^{v}_{f1}]C^{v}_{f1}-(\gamma _{f1}+d_{f1}+\mu _{f1}+\alpha _{f})D_{f1},\nonumber \\ \frac{\textrm{d}R_{f1}}{dt}&=\gamma _{f1}D_{f1}+r_{f1}^{u}\rho I^{u}_{f1}+e_{f1}^{u}\eta L^{u}_{f1}+r_{f1}^{v}\rho I^{v}_{f1}+e_{f1}^{v}\eta L^{v}_{f1}-\mu _{f1}R_{f1}-\alpha _{f}R_{f1}-\zeta _{f1}R_{f1},\end{aligned}$$

2 $$\begin{aligned} \frac{\textrm{d}S^{u}_{f2}}{dt}&=\alpha _{f}S^{u}_{f1}-\lambda _{m2}S^{u}_{f2}+\delta S^{v}_{f2}-\mu _{f2} S^{u}_{f2}+\zeta _{f2}R_{f2},\nonumber \\ \frac{\textrm{d}I^{u}_{f2}}{dt}&=\alpha _{f}I^{u}_{f1}+\lambda _{m2}S^{u}_{f2}-\rho I^{u}_{f2}-\mu _{f2} I^{u}_{f2},\nonumber \\ \frac{\textrm{d}L^{u}_{f2}}{dt}&=\alpha _{f}L^{u}_{f1}+(1-r_{f2}^{u})\rho I^{u}_{f2}-\eta L^{u}_{f2}-\sigma _{f2}^{u}L^{u}_{f2}-\mu _{f2} L^{u}_{f2},\nonumber \\ \frac{\textrm{d}H^{u}_{f2}}{dt}&=\alpha _{f}H^{u}_{f1}+(1-e_{f2}^{u})\eta L^{u}_{f2}-\xi ^{u}_{f2} H^{u}_{f2}-\sigma ^{u}_{f2}H^{u}_{f2}-\mu _{f2}H^{u}_{f2},\nonumber \\ \frac{\textrm{d}C^{u}_{f2}}{dt}&=\alpha _{f}C^{u}_{f1}+\xi ^{u}_{f2} H^{u}_{f2}-\Lambda C^{u}_{f2} -(1-\Lambda )\sigma ^{u}_{f2}C^{u}_{f2}-\mu _{f2}C^{u}_{f2},\nonumber \\ \frac{\textrm{d}S^{v}_{f2}}{dt}&=\alpha _{f}S^{v}_{f1}-(1-\varepsilon _{f2})\lambda _{m2}S^{v}_{f2}-\delta S^{v}_{f2}-\mu _{f2} S^{v}_{f2},\nonumber \\ \frac{\textrm{d}I^{v}_{f2}}{dt}&=\alpha _{f}I^{v}_{f1}+(1-\varepsilon _{f2})\lambda _{m2}S^{v}_{f2}-\rho I^{v}_{f2}-\mu _{f2} I^{v}_{f2},\nonumber \\ \frac{\textrm{d}L^{v}_{f2}}{dt}&=\alpha _{f}L^{v}_{f1}+(1-r_{f2}^{v})\rho I^{v}_{f2}-\eta L^{v}_{f2}-\sigma _{f2}^{v}L^{v}_{f2}-\mu _{f2} L^{v}_{f2},\nonumber \\ \frac{\textrm{d}H^{v}_{f2}}{dt}&=\alpha _{f}H^{v}_{f1}+(1-e_{f2}^{v})\eta L^{v}_{f2}-\xi ^{v}_{f2} H^{v}_{f2}-\sigma ^{v}_{f2}H^{v}_{f2}-\mu _{f2}H^{v}_{f2},\nonumber \\ \frac{\textrm{d}C^{v}_{f2}}{dt}&=\alpha _{f}C^{v}_{f1}+\xi ^{v}_{f2} H^{v}_{f2}-\Lambda C^{v}_{f2}-(1-\Lambda )\sigma ^{v}_{f2}C^{v}_{f2}-\mu _{f2}C^{v}_{f2},\nonumber \\ \frac{\textrm{d}D_{f2}}{dt}&=\alpha _{f}D_{f1}+\sigma ^{u}_{f2}(L^{u}_{f2}+H^{u}_{f2}) +[\Lambda +(1-\Lambda )\sigma ^{u}_{f2}]C^{u}_{f2}+\sigma ^{v}_{f2}(L^{v}_{f2}+H^{v}_{f2})\nonumber \\&\quad +[\Lambda +(1-\Lambda )\sigma ^{v}_{f2}]C^{v}_{f2}-(\gamma _{f2}+d_{f2}+\mu _{f2})D_{f2},\nonumber \\ \frac{\textrm{d}R_{f2}}{dt}&=\alpha _{f}R_{f1}+\gamma _{f2}D_{f2}+r_{f2}^{u}\rho I^{u}_{f2}+e_{f2}^{u}\eta L^{u}_{f2}+r_{f2}^{v}\rho I^{v}_{f2}+e_{f2}^{v}\eta L^{v}_{f2}-\mu _{f2}R_{f2}-\zeta _{f2}R_{f2}, \end{aligned}$$

3 $$\begin{aligned} \frac{\textrm{d}S^{u}_{m1}}{dt}&=A_{m}(1-\phi _{m})-\lambda _{f1}S^{u}_{m1}-k_{m}S_{m1}^{u}+\delta S^{v}_{m1}-\mu _{m1} S^{u}_{m1}-\alpha _{m}S^{u}_{m1}+\zeta _{m1}R_{m1},\nonumber \\ \frac{\textrm{d}I^{u}_{m1}}{dt}&=\lambda _{f1}S^{u}_{m1}-\omega ^{u}_{m1} I^{u}_{m1}-\mu _{m1} I^{u}_{m1}-\alpha _{m}I^{u}_{m1},\nonumber \\ \frac{\textrm{d}S^{v}_{m1}}{dt}&=A_{m}\phi _{m}-(1-\varepsilon _{m1})\lambda _{f1}S^{v}_{m1}+k_{m}S_{m1}^{u}-\delta S^{v}_{m1}-\mu _{m1} S^{v}_{m1}-\alpha _{m}S^{v}_{m1},\nonumber \\ \frac{\textrm{d}I^{v}_{m1}}{dt}&=(1-\varepsilon _{m1})\lambda _{f1}S^{v}_{m1}-\omega ^{v}_{m1} I^{v}_{m1}-\mu _{m1} I^{v}_{m1}-\alpha _{m}I^{v}_{m1},\nonumber \\ \frac{\textrm{d}R_{m1}}{dt}&=\omega ^{u}_{m1} I^{u}_{m1}+\omega ^{v}_{m1} I^{v}_{m1}-\mu _{m1}R_{m1}-\alpha _{m}R_{m1}-\zeta _{m1}R_{m1},\end{aligned}$$

4 $$\begin{aligned} \frac{\textrm{d}S^{u}_{m2}}{dt}&=\alpha _{m}S^{u}_{m1}-\lambda _{f2}S^{u}_{m2}+\delta S^{v}_{m2}-\mu _{m2} S^{u}_{m2}+\zeta _{m2}R_{m2},\nonumber \\ \frac{\textrm{d}I^{u}_{m2}}{dt}&=\alpha _{m}I^{u}_{m1}+\lambda _{f2}S^{u}_{m2}-\omega ^{u}_{m2} I^{u}_{m2}-\mu _{m2} I^{u}_{m2},\nonumber \\ \frac{\textrm{d}S^{v}_{m2}}{dt}&=\alpha _{m}S^{v}_{m1}-(1-\varepsilon _{m2})\lambda _{f2}S^{v}_{m2}-\delta S^{v}_{m2}-\mu _{m2} S^{v}_{m2},\nonumber \\ \frac{\textrm{d}I^{v}_{m2}}{dt}&=\alpha _{m}I^{v}_{m1}+(1-\varepsilon _{m2})\lambda _{f2}S^{v}_{m2}-\omega ^{v}_{m2} I^{v}_{m2}-\mu _{m2} I^{v}_{m2},\nonumber \\ \frac{\textrm{d}R_{m2}}{dt}&=\alpha _{m}R_{m1}+\omega ^{u}_{m2} I^{u}_{m2}+\omega ^{v}_{m2} I^{v}_{m2}-\mu _{m2}R_{m2}-\zeta _{m2}R_{m2}. \end{aligned}$$

Denote $\mathbb {R}_{+}^{34}$ as the cone composed of all nonnegative vectors in $\mathbb {R}^{34}$ and

$$\begin{aligned} x=(&S^{u}_{f1}, I^{u}_{f1}, L^{u}_{f1}, H^{u}_{f1}, C^{u}_{f1}, S^{v}_{f1}, I^{v}_{f1}, L^{v}_{f1}, H^{v}_{f1}, C^{v}_{f1},D_{f1}, R_{f1}, S^{u}_{f2}, I^{u}_{f2}, L^{u}_{f2}, H^{u}_{f2}, C^{u}_{f2}, S^{v}_{f2}, I^{v}_{f2}, L^{v}_{f2}, H^{v}_{f2}, C^{v}_{f2}, D_{f2}, R_{f2},\\ &S^{u}_{m1}, I^{u}_{m1}, S^{v}_{m1}, I^{v}_{m1}, R_{m1}, S^{u}_{m2}, I^{u}_{m2}, S^{v}_{m2}, I^{v}_{m2}, R_{m2}). \end{aligned}$$

Here, $x(0)\in \mathbb {R}_{+}^{34}$ for system (1)–(4).

**Table 2:** Description of parameters in model (1)–(4).

Parameters	Description
$A_f(A_m)$	The growth rate of females (males) in the first age group per unit time
$\mu _{fi}(\mu _{mi})$	Natural mortality coefficient for females (males) in the *i-*th age group per unit time ($i\in \{1,2\}$)
$d_{fi}$	Mortality coefficient due to disease for females in the *i-*th age group per unit time
$\phi _f(\phi _m)$	Proportion of 14-year-old girls (boys) vaccinated
$k_f(k_m)$	Vaccination rate coefficient for females (males) in the first age group
$\delta$	Antibody attenuation rate coefficient
$\varepsilon _{fi}(\varepsilon _{mi})$	The protective efficacy of the vaccine against females (males) in the *i-*th age group
$\alpha _f(\alpha _m)$	Transfer rate coefficient for females (males) in the first age group to the second age group
$\omega _{mi}^{u}(\omega _{mi}^{v})$	Recovery rate coefficient for unvaccinated (vaccinated) infected males in the *i-*th age group
$\rho$	Removal rate coefficient for infected females ($I_f$)
$r_{fi}^{u}(r_{fi}^{v})$	The proportion of unvaccinated (vaccinated) infected women who do not develop LSIL
$\eta$	Removal rate coefficient for females with LSIL due to development of HSIL or self-recovery
$e_{fi}^{u}(e_{fi}^{v})$	Self-recovery proportion of unvaccinated (vaccinated) women with LSIL in the *i-*th age group
$\xi _{fi}^{u}(\xi _{fi}^{v})$	Coefficient of transfer rate from HSIL to CC among unvaccinated (vaccinated) females in the *i-*th age group
$\sigma _{fi}^{u}(\sigma _{fi}^{v})$	CC screening rate among unvaccinated (vaccinated) women in the *i-*th age group
$\Lambda$	Proportion of symptomatic CC patients
$\gamma _{fi}$	Recovery rate coefficient for females in $D_{fi}$
$\zeta _{fi}$	Loss rate of infection-acquired immunity for females in $R_{fi}$
$\zeta _{mi}$	Loss rate of infection-acquired immunity for males in $R_{mi}$
$\beta _{m}$	Probability of transmission from a female to a susceptible male per sexual contact
$\beta _{f}$	Probability of transmission from a male to a susceptible female per sexual contact
$c_{mij}$	The average number of sexual contacts per unit time between a male in the *i-*th age group and females in the *j-*th age group ($i=1,2$; $j=1,2$)
$c_{fij}$	The average number of sexual contacts per unit time between a female in the *i-*th age group and males in the *j-*th age group ($i=1,2$; $j=1,2$)

### Analysis of the model

#### Theorem 1

*For any* $x(0)\in \mathbb {R}_{+}^{34}$, *system* (1)–(4) *has a unique nonnegative solution defined on* $[0,\infty )$. *Furthermore, the solution is ultimately bounded, that is*,

$$\begin{aligned} \limsup \limits _{t\rightarrow +\infty }N_{f}(t)\le \frac{A_{f}}{\min \{\mu _{f1}, \mu _{f2}\}} \ \text {and}\ \limsup \limits _{t\rightarrow +\infty }N_{m}(t)\le \frac{A_{m}}{\min \{\mu _{m1}, \mu _{m2}\}}. \end{aligned}$$

*And* $\mathbb {D}=\left\{ x\in \mathbb {R}_{+}^{34} : N_{f}\le \frac{A_{f}}{\min \{\mu _{f1},\ \mu _{f2}\}}\ \text {and}\ N_{m}\le \frac{A_{m}}{\min \{\mu _{m1}, \mu _{m2}\}} \right\}$ *is the positively invariant set for system* (1)–(4).

The proof of Theorem 1 can be found in the Appendix 1.

Let $I_{fi}^{g}=0$, $L_{fi}^{g}=0$, $H_{fi}^{g}=0$, $C_{fi}^{g}=0$ and $I_{mi}^{g}=0$, where $g\in \{u,v\}$ and $i\in \{1,2\}$, and let all the right sides of equations (1–4) be equal to 0. Then, by calculation, it is easy to see that the disease-free equilibrium of system (1)–(4) is

$$\begin{aligned} \bar{E}=(\bar{S}^{u}_{f1}, 0, 0, 0, 0, \bar{S}^{v}_{f1}, 0, 0, 0, 0, 0, 0, \bar{S}^{u}_{f2}, 0, 0, 0, 0, \bar{S}^{v}_{f2}, 0, 0, 0, 0, 0, 0, \bar{S}^{u}_{m1}, 0, \bar{S}^{v}_{m1}, 0, 0, \bar{S}^{u}_{m2}, 0, \bar{S}^{v}_{m2}, 0, 0), \end{aligned}$$

where

$$\begin{aligned} \bar{S}^{u}_{f1}&=\frac{A_{f}[(1-\phi _{f})(\mu _{f1}+\alpha _{f})+\delta ]}{(k_{f}+\delta +\mu _{f1}+\alpha _{f})(\mu _{f1}+\alpha _{f})},\\ \bar{S}^{v}_{f1}&=\frac{A_{f}[\phi _{f}(\mu _{f1}+\alpha _{f})+k_{f}]}{(k_{f}+\delta +\mu _{f1}+\alpha _{f})(\mu _{f1}+\alpha _{f})},\\ \bar{S}^{u}_{f2}&=\frac{\alpha _{f}A_{f}[\delta (\delta +\mu _{f2}+k_{f}+\mu _{f1}+\alpha _{f})+\mu _{f2}(1-\phi _{f})(\mu _{f1}+\alpha _{f})]}{\mu _{f2}(k_{f}+\delta +\mu _{f1}+\alpha _{f})(\mu _{f1}+\alpha _{f})(\delta +\mu _{f2})},\\ \bar{S}^{v}_{f2}&=\frac{\alpha _{f}A_{f}[\phi _{f}(\mu _{f1}+\alpha _{f})+k_{f}]}{(k_{f}+\delta +\mu _{f1}+\alpha _{f})(\mu _{f1}+\alpha _{f})(\delta +\mu _{f2})},\\ \bar{S}^{u}_{m1}&=\frac{A_{m}[(1-\phi _{m})(\mu _{m1}+\alpha _{m})+\delta ]}{(k_{m}+\delta +\mu _{m1}+\alpha _{m})(\mu _{m1}+\alpha _{m})},\\ \bar{S}^{v}_{m1}&=\frac{A_{m}[\phi _{m}(\mu _{m1}+\alpha _{m})+k_{m}]}{(k_{m}+\delta +\mu _{m1}+\alpha _{m})(\mu _{m1}+\alpha _{m})}, \end{aligned}$$

$$\begin{aligned} \bar{S}^{u}_{m2}&=\frac{\alpha _{m}A_{m}[\delta (\delta +\mu _{m2}+k_{m}+\mu _{m1}+\alpha _{m})+\mu _{m2}(1-\phi _{m})(\mu _{m1}+\alpha _{m})]}{\mu _{m2}(k_{m}+\delta +\mu _{m1}+\alpha _{m})(\mu _{m1}+\alpha _{m})(\delta +\mu _{m2})},\\ \bar{S}^{v}_{m2}&=\frac{\alpha _{m}A_{m}[\phi _{m}(\mu _{m1}+\alpha _{m})+k_{m}]}{(k_{m}+\delta +\mu _{m1}+\alpha _{m})(\mu _{m1}+\alpha _{m})(\delta +\mu _{m2})}. \end{aligned}$$

Further, it follows that

$$\begin{aligned}&\bar{S}^{u}_{f1}+\bar{S}^{v}_{f1}=\frac{A_{f}}{\mu _{f1}+\alpha _{f}}:=\bar{N}_{f1},\qquad \,\, \bar{S}^{u}_{f2}+\bar{S}^{v}_{f2}=\frac{\alpha _{f}A_{f}}{\mu _{f2}(\mu _{f1}+\alpha _{f})}:=\bar{N}_{f2},\\&\bar{S}^{u}_{m1}+\bar{S}^{v}_{m1}=\frac{A_{m}}{\mu _{m1}+\alpha _{m}}:=\bar{N}_{m1},\quad \bar{S}^{u}_{m2}+\bar{S}^{v}_{m2}=\frac{\alpha _{m}A_{m}}{\mu _{m2}(\mu _{m1}+\alpha _{m})}:=\bar{N}_{m2}. \end{aligned}$$

Next, we calculate the basic reproduction number according to the next-generation matrix method^28^. We need only to consider the following compartments that can transmit HPV

$$\begin{aligned} {\mathbf {y}}=[I^{u}_{f1}, L^{u}_{f1}, H^{u}_{f1}, C^{u}_{f1}, I^{v}_{f1}, L^{v}_{f1}, H^{v}_{f1}, C^{v}_{f1}, I^{u}_{f2}, L^{u}_{f2}, H^{u}_{f2}, C^{u}_{f2}, I^{v}_{f2}, L^{v}_{f2}, H^{v}_{f2}, C^{v}_{f2}, I^{u}_{m1}, I^{v}_{m1}, I^{u}_{m2}, I^{v}_{m2}]. \end{aligned}$$

We denote

$$\begin{aligned} s^{u}_{f1}&=\frac{\bar{S}^{u}_{f1}}{\bar{N}_{f1}}=\frac{(1-\phi _{f})(\mu _{f1}+\alpha _{f})+\delta }{k_{f}+\delta +\mu _{f1}+\alpha _{f}},\\ s^{v}_{f1}&=\frac{\bar{S}^{v}_{f1}}{\bar{N}_{f1}}=\frac{\phi _{f}(\mu _{f1}+\alpha _{f})+k_{f}}{k_{f}+\delta +\mu _{f1}+\alpha _{f}},\\ s^{u}_{f2}&=\frac{\bar{S}^{u}_{f2}}{\bar{N}_{f2}}=\frac{\delta (\delta +\mu _{f2}+k_{f}+\mu _{f1}+\alpha _{f})+\mu _{f2}(1-\phi _{f})(\mu _{f1}+\alpha _{f})}{(k_{f}+\delta +\mu _{f1}+\alpha _{f})(\delta +\mu _{f2})},\\ s^{v}_{f2}&=\frac{\bar{S}^{v}_{f2}}{\bar{N}_{f2}}=\frac{\mu _{f2}[\phi _{f}(\mu _{f1}+\alpha _{f})+k_{f}]}{(k_{f}+\delta +\mu _{f1}+\alpha _{f})(\delta +\mu _{f2})},\\ s^{u}_{m1}&=\frac{\bar{S}^{u}_{m1}}{\bar{N}_{m1}}=\frac{(1-\phi _{m})(\mu _{m1}+\alpha _{m})+\delta }{k_{m}+\delta +\mu _{m1}+\alpha _{m}},\\ s^{v}_{m1}&=\frac{\bar{S}^{v}_{m1}}{\bar{N}_{m1}}=\frac{\phi _{m}(\mu _{m1}+\alpha _{m})+k_{m}}{k_{m}+\delta +\mu _{m1}+\alpha _{m}},\\ s^{u}_{m2}&=\frac{\bar{S}^{u}_{m2}}{\bar{N}_{m2}}=\frac{\delta (\delta +\mu _{m2}+k_{m}+\mu _{m1}+\alpha _{m})+\mu _{m2}(1-\phi _{m})(\mu _{m1}+\alpha _{m})}{(k_{m}+\delta +\mu _{m1}+\alpha _{m})(\delta +\mu _{m2})},\\ s^{v}_{m2}&=\frac{\bar{S}^{v}_{m2}}{\bar{N}_{m2}}=\frac{\mu _{m2}[\phi _{m}(\mu _{m1}+\alpha _{m})+k_{m}]}{(k_{m}+\delta +\mu _{m1}+\alpha _{m})(\delta +\mu _{m2})}, \end{aligned}$$

where $s^{u}_{fi}$ ($s^{v}_{fi}$) represents the proportion of susceptible individuals (vaccinated individuals) in the *i-*th age group for female; $s^{u}_{mi}$ ($s^{v}_{mi}$) represents the proportion of susceptible individuals (vaccinated individuals) in the *i-*th age group for male.

Calculating the partial derivatives of the new infection terms and transition terms of **y** at the disease-free equilibrium $\bar{E}$, we derive that

$$\begin{aligned}F=\left[ \begin{matrix} {{\varvec{O}}}_{8\times 8} & {{\varvec{O}}}_{8\times 8} & {{\varvec{P}}}_{8\times 4} \\ {{\varvec{O}}}_{8\times 8} & {{\varvec{O}}}_{8\times 8} & {{\varvec{Q}}}_{8\times 4} \\ {{\varvec{W}}}_{4\times 8} & {{\varvec{Z}}}_{4\times 8} & {{\varvec{O}}}_{4\times 4} \\ \end{matrix} \right] \ \text {and} \ V=\left[ \begin{matrix} {{\varvec{X}}}_{8\times 8} & {{\varvec{O}}}_{8\times 8} & {{\varvec{O}}}_{8\times 4} \\ {{\varvec{Y}}}_{8\times 8} & {{\varvec{M}}}_{8\times 8} & {{\varvec{O}}}_{8\times 4} \\ {{\varvec{O}}}_{4\times 8} & {{\varvec{O}}}_{4\times 8} & {{\varvec{G}}}_{4\times 4} \\ \end{matrix} \right] , \end{aligned}$$

where

$$\begin{aligned} & {{\varvec{P}}}=\left[ \begin{matrix} f_{11} & f_{11} & f_{21} & f_{21}\\ 0 & 0 & 0 & 0 \\ 0 & 0 & 0 & 0 \\ 0 & 0 & 0 & 0 \\ f_{12} & f_{12} & f_{22} & f_{22}\\ 0 & 0 & 0 & 0 \\ 0 & 0 & 0 & 0 \\ 0 & 0 & 0 & 0 \\ \end{matrix} \right] , {{\varvec{Q}}}=\left[ \begin{matrix} f_{31} & f_{31} & f_{41} & f_{41}\\ 0 & 0 & 0 & 0 \\ 0 & 0 & 0 & 0 \\ 0 & 0 & 0 & 0 \\ f_{32} & f_{32} & f_{42} & f_{42}\\ 0 & 0 & 0 & 0 \\ 0 & 0 & 0 & 0 \\ 0 & 0 & 0 & 0 \\ \end{matrix} \right] , \end{aligned}$$

$$\begin{aligned} & {{\varvec{W}}}=\left[ \begin{matrix} f_{51} & f_{51} & f_{51} & f_{51} & f_{51} & f_{51} & f_{51} & f_{51} \\ f_{52} & f_{52} & f_{52} & f_{52} & f_{52} & f_{52} & f_{52} & f_{52} \\ f_{71} & f_{71} & f_{71} & f_{71} & f_{71} & f_{71} & f_{71} & f_{71} \\ f_{72} & f_{72} & f_{72} & f_{72} & f_{72} & f_{72} & f_{72} & f_{72} \end{matrix} \right] , {{\varvec{Z}}}=\left[ \begin{matrix} f_{61} & f_{61} & f_{61} & f_{61} & f_{61} & f_{61} & f_{61} & f_{61} \\ f_{62} & f_{62} & f_{62} & f_{62} & f_{62} & f_{62} & f_{62} & f_{62} \\ f_{81} & f_{81} & f_{81} & f_{81} & f_{81} & f_{81} & f_{81} & f_{81} \\ f_{82} & f_{82} & f_{82} & f_{82} & f_{82} & f_{82} & f_{82} & f_{82} \end{matrix} \right] , \\ & \quad {\mathbf {X}}=\left[ \begin{matrix} X_{1} & 0 & 0 & 0 & 0 & 0 & 0 & 0\\ -(1-r_{f1}^{u})\rho & X_{2} & 0 & 0 & 0 & 0 & 0 & 0 \\ 0 & -(1-e_{f1}^{u})\eta & X_{3}& 0 & 0 & 0 & 0 & 0 \\ 0 & 0 & -\xi ^{u}_{f1} & X_{4} & 0 & 0 & 0 & 0 \\ 0 & 0 & 0 & 0 & X_{5}& 0 & 0 & 0 \\ 0 & 0 & 0 & 0 & -(1-r_{f1}^{v})\rho & X_{6}& 0 & 0 \\ 0 & 0 & 0 & 0 & 0 & -(1-e_{f1}^{v})\eta & X_{7}& 0 \\ 0 & 0 & 0 & 0 & 0 & 0 & -\xi ^{v}_{f1} & X_{8}\\ \end{matrix} \right] , \\ & \quad {{\varvec{Y}}}=\text {diag}\{-\alpha _{f}, -\alpha _{f}, -\alpha _{f}, -\alpha _{f}, -\alpha _{f}, -\alpha _{f}, -\alpha _{f}, -\alpha _{f}\}, \\ & \quad {{\varvec{M}}}=\left[ \begin{matrix} M_{1}& 0 & 0 & 0 & 0 & 0& 0 & 0 \\ -(1-r_{f2}^{u})\rho & M_{2}& 0 & 0 & 0 & 0& 0 & 0 \\ 0 & -(1-e_{f2}^{u})\eta & M_{3}& 0 & 0 & 0& 0 & 0 \\ 0 & 0 & -\xi ^{u}_{f2} & M_{4}& 0 & 0& 0 & 0 \\ 0 & 0 & 0 & 0 & M_{5} & 0& 0 & 0\\ 0 & 0 & 0 & 0 & -(1-r_{f2}^{v})\rho & M_{6}& 0 & 0 \\ 0 & 0 & 0 & 0 & 0 & -(1-e_{f2}^{v})\eta & M_{7}& 0 \\ 0 & 0 & 0 & 0 & 0 & 0 & -\xi ^{v}_{f2} & M_{8}\\ \end{matrix} \right] , \\ & \quad {{\varvec{G}}}=\left[ \begin{matrix} G_{1}& 0 & 0 & 0 \\ 0 & G_{2}& 0 & 0 \\ -\alpha _{m}& 0 & G_{3}& 0 \\ 0 & -\alpha _{m} & 0 & G_{4} \end{matrix} \right] , \end{aligned}$$

in which

$$\begin{aligned} f_{11}&=\beta _{m}c_{m11}s^{u}_{f1},&\quad f_{12}&=(1-\varepsilon _{f1})\beta _{m}c_{m11}s^{v}_{f1},&\quad f_{21}&=\beta _{m}c_{m21}s^{u}_{f1},\\ f_{22}&=(1-\varepsilon _{f1})\beta _{m}c_{m21}s^{v}_{f1},&f_{31}&=\beta _{m}c_{m12}s^{u}_{f2},&f_{32}&=(1-\varepsilon _{f2})\beta _{m}c_{m12}s^{v}_{f2},\\ f_{41}&=\beta _{m}c_{m22}s^{u}_{f2},&f_{42}&=(1-\varepsilon _{f2})\beta _{m}c_{m22}s^{v}_{f2},&f_{51}&=\beta _{f}c_{f11}s^{u}_{m1},\\ f_{52}&=(1-\varepsilon _{m1})\beta _{f}c_{f11}s^{v}_{m1},&f_{61}&=\beta _{f}c_{f21}s^{u}_{m1},&f_{62}&=(1-\varepsilon _{m1})\beta _{f}c_{f21}s^{v}_{m1},\\ f_{71}&=\beta _{f}c_{f12}s^{u}_{m2},&f_{72}&=(1-\varepsilon _{m2})\beta _{f}c_{f12}s^{v}_{m2},&f_{81}&=\beta _{f}c_{f22}s^{u}_{m2},\\ f_{82}&=(1-\varepsilon _{m2})\beta _{f}c_{f22}s^{v}_{m2},&X_{1}&=\rho +\mu _{f1}+\alpha _{f},&X_{2}&=\eta +\sigma _{f1}^{u}+\mu _{f1}+\alpha _{f},\\ X_{3}&=\xi ^{u}_{f1}+\sigma ^{u}_{f1}+\mu _{f1}+\alpha _{f},&X_{4}&=\Lambda +(1-\Lambda )\sigma ^{u}_{f1}+\mu _{f1}+\alpha _{f},&X_{5}&=\rho +\mu _{f1}+\alpha _{f},\\ X_{6}&=\eta +\sigma _{f1}^{v}+\mu _{f1}+\alpha _{f},&X_{7}&=\xi ^{v}_{f1}+\sigma ^{v}_{f1}+\mu _{f1}+\alpha _{f},&X_{8}&=\Lambda +(1-\Lambda )\sigma ^{v}_{f1}+\mu _{f1}+\alpha _{f},\\ M_{1}&=\rho +\mu _{f2},&M_{2}&=\eta +\sigma _{f2}^{u}+\mu _{f2},&M_{3}&=\xi ^{u}_{f2}+\sigma ^{u}_{f2}+\mu _{f2},\\ M_{4}&=\Lambda +(1-\Lambda )\sigma ^{u}_{f2}+\mu _{f2},&M_{5}&=\rho +\mu _{f2},&M_{6}&=\eta +\sigma _{f2}^{v}+\mu _{f2},\\ M_{7}&=\xi ^{v}_{f2}+\sigma ^{v}_{f2}+\mu _{f2},&M_{8}&=\Lambda +(1-\Lambda )\sigma ^{v}_{f2}+\mu _{f2},&G_{1}&=\omega ^{u}_{m1}+\mu _{m1}+\alpha _{m},\\ G_{2}&=\omega ^{v}_{m1}+\mu _{m1}+\alpha _{m},&G_{3}&=\omega ^{u}_{m2}+\mu _{m2},&G_{4}&=\omega ^{v}_{m2}+\mu _{m2}. \end{aligned}$$

Then, the next generation matrix is of the form

$$\begin{aligned}FV^{-1}=\left[ \begin{matrix} {{\varvec{O}}}_{8\times 8} & {{\varvec{O}}}_{8\times 8} & {{\varvec{P}}}{{\varvec{G}}}^{-1}\\ {{\varvec{O}}}_{8\times 8} & {{\varvec{O}}}_{8\times 8} & {{\varvec{Q}}}{{\varvec{G}}}^{-1}\\ {{\varvec{W}}}{{\varvec{X}}}^{-1}-{{\varvec{Z}}}{{\varvec{M}}}^{-1}{{\varvec{Y}}}{{\varvec{X}}}^{-1} & {{\varvec{Z}}}{{\varvec{M}}}^{-1} & {{\varvec{O}}}_{4\times 4} \end{matrix} \right] . \end{aligned}$$

The characteristic equation of $FV^{-1}$ is $|\Lambda {{\varvec{I}}}-FV^{-1}|=\Lambda ^{12}|{{\varvec{U}}}|=0$, where

$$\begin{aligned}|{{\varvec{U}}}|= \left| \begin{matrix} \Lambda & 0 & 0 & 0 & -u_{15} & -u_{16} & -u_{17} & -u_{18}\\ 0 & \Lambda & 0 & 0 & -u_{25} & -u_{26} & -u_{27}& -u_{28}\\ 0 & 0 & \Lambda & 0 & -u_{35} & -u_{36} & -u_{37} & -u_{38} \\ 0 & 0 & 0 & \Lambda & -u_{45} & -u_{46} & -u_{47} & -u_{48} \\ -u_{51} & -u_{52} & -u_{53} & -u_{54} & \Lambda & 0 & 0 & 0\\ -u_{61} & -u_{62} & -u_{63} & -u_{64} & 0 & \Lambda & 0 & 0\\ -u_{71} & -u_{72} & -u_{73} & -u_{74} & 0 & 0 & \Lambda & 0\\ -u_{81} & -u_{82} & -u_{83} & -u_{84} & 0 & 0 & 0 & \Lambda \\ \end{matrix} \right| , \end{aligned}$$

in which

$$\begin{aligned} u_{15}&=\frac{f_{11}}{G_{1}}+\frac{f_{21}\alpha _{m}}{G_{1}G_{3}},&\quad u_{16}&=\frac{f_{11}}{G_{2}}+\frac{f_{21}\alpha _{m}}{G_{2}G_{4}},&\quad u_{17}&=\frac{f_{21}}{G_{3}},&\quad u_{18}&=\frac{f_{21}}{G_{4}},\\ u_{25}&=\frac{f_{12}}{G_{1}}+\frac{f_{22}\alpha _{m}}{G_{1}G_{3}},&u_{26}&=\frac{f_{12}}{G_{2}}+\frac{f_{22}\alpha _{m}}{G_{2}G_{4}},&u_{27}&=\frac{f_{22}}{G_{3}},&u_{28}&=\frac{f_{22}}{G_{4}},\\ u_{35}&=\frac{f_{31}}{G_{1}}+\frac{f_{41}\alpha _{m}}{G_{1}G_{3}},&u_{36}&=\frac{f_{31}}{G_{2}}+\frac{f_{41}\alpha _{m}}{G_{2}G_{4}},&u_{37}&=\frac{f_{41}}{G_{3}},&u_{38}&=\frac{f_{41}}{G_{4}},\\ u_{45}&=\frac{f_{32}}{G_{1}}+\frac{f_{42}\alpha _{m}}{G_{1}G_{3}},&u_{46}&=\frac{f_{32}}{G_{2}}+\frac{f_{42}\alpha _{m}}{G_{2}G_{4}},&u_{47}&=\frac{f_{42}}{G_{3}},&u_{48}&=\frac{f_{42}}{G_{4}},\\ u_{51}&=f_{51}a_{1}+f_{61}a_{5},&u_{52}&=f_{51}a_{2}+f_{61}a_{6},&u_{53}&=f_{61}a_{3},&u_{54}&=f_{61}a_{4},\\ u_{61}&=f_{52}a_{1}+f_{62}a_{5},&u_{62}&=f_{52}a_{2}+f_{62}a_{6},&u_{63}&=f_{62}a_{3},&u_{64}&=f_{62}a_{4},\\ u_{71}&=f_{71}a_{1}+f_{81}a_{5},&u_{72}&=f_{71}a_{2}+f_{81}a_{6},&u_{73}&=f_{81}a_{3},&u_{74}&=f_{81}a_{4},\\ u_{81}&=f_{72}a_{1}+f_{82}a_{5},&u_{82}&=f_{72}a_{2}+f_{82}a_{6},&u_{83}&=f_{82}a_{3},&u_{84}&=f_{82}a_{4}, \end{aligned}$$

here

$$\begin{aligned} a_{1}&=\frac{1}{X_{1}}+\frac{(1-r_{f1}^{u})\rho }{X_{1}X_{2}}+\frac{(1-r_{f1}^{u})\rho (1-e_{f1}^{u})\eta }{X_{1}X_{2}X_{3}}+\frac{(1-r_{f1}^{u})\rho (1-e_{f1}^{u})\eta \xi ^{u}_{f1}}{X_{1}X_{2}X_{3}X_{4}},\\ a_{2}&=\frac{1}{X_{5}}+\frac{(1-r_{f1}^{v})\rho }{X_{5}X_{6}}+\frac{(1-r_{f1}^{v})\rho (1-e_{f1}^{v})\eta }{X_{5}X_{6}X_{7}}+\frac{(1-r_{f1}^{v})\rho (1-e_{f1}^{v})\eta \xi ^{v}_{f1}}{X_{5}X_{6}X_{7}X_{8}},\\ a_{3}&=\frac{1}{M_{1}}+\frac{(1-r_{f2}^{u})\rho }{M_{1}M_{2}}+\frac{(1-r_{f2}^{u})\rho (1-e_{f2}^{u})\eta }{M_{1}M_{2}M_{3}}+\frac{(1-r_{f2}^{u})\rho (1-e_{f2}^{u})\eta \xi ^{u}_{f2}}{M_{1}M_{2}M_{3}M_{4}},\\ a_{4}&=\frac{1}{M_{5}}+\frac{(1-r_{f2}^{v})\rho }{M_{5}M_{6}}+\frac{(1-r_{f2}^{v})\rho (1-e_{f2}^{v})\eta }{M_{5}M_{6}M_{7}}+\frac{(1-r_{f2}^{v})\rho (1-e_{f2}^{v})\eta \xi ^{v}_{f2}}{M_{5}M_{6}M_{7}M_{8}},\\ a_{5}&=\frac{\alpha _{f}}{X_{1}}a_{3},\quad a_{6}=\frac{\alpha _{f}}{X_{5}}a_{4}. \end{aligned}$$

Further, it is easy to get the basic reproduction number^28^

$$\begin{aligned} \mathcal {R}_{0}=\rho (FV^{-1})=\max \{|\Lambda |\mid |{{\varvec{U}}}|=0\}. \end{aligned}$$

The meaning of the basic reproduction number is the number of people that an infected person can infect during its infectious period when all people are susceptible. In order to better understand the meaning of the basic reproduction number, we explain the meaning of the elements in the matrix ${{\varvec{U}}}$. $1/G_{1}$, $1/G_{2}$, $1/G_{3}$ and $1/G_{4}$ are the average illness period of infected individuals in compartments $I^{u}_{m1}$, $I^{v}_{m1}$, $I^{u}_{m2}$ and $I^{v}_{m2}$, respectively. $\alpha _{m}/G_{1}$ ($\alpha _{m}/G_{2}$) is the probability that individuals in compartment $I^{u}_{m1}$ $\left( I^{v}_{m1}\right)$ transfer to compartment $I^{u}_{m2}$ ($I^{v}_{m2}$) due to the increase of age. $u_{15}$, $u_{16}$, $u_{17}$ and $u_{18}$ represent the number of females in compartment $S^{u}_{f1}$ infected by a male belonging to compartments $I^{u}_{m1}$, $I^{v}_{m1}$, $I^{u}_{m2}$ and $I^{v}_{m2}$ during his illness period, at the beginning of the epidemic, respectively. $u_{25}$, $u_{26}$, $u_{27}$ and $u_{28}$ represent the number of females in compartment $S^{v}_{f1}$ infected by a male belonging to compartments $I^{u}_{m1}$, $I^{v}_{m1}$, $I^{u}_{m2}$ and $I^{v}_{m2}$ during his illness period, at the beginning of the epidemic, respectively. $u_{35}$, $u_{36}$, $u_{37}$ and $u_{38}$ represent the number of females in compartment $S^{u}_{f2}$ infected by a male belonging to compartments $I^{u}_{m1}$, $I^{v}_{m1}$, $I^{u}_{m2}$ and $I^{v}_{m2}$ during his illness period, at the beginning of the epidemic, respectively. $u_{45}$, $u_{46}$, $u_{47}$ and $u_{48}$ represent the number of females in compartment $S^{v}_{f2}$ infected by a male belonging to compartments $I^{u}_{m1}$, $I^{v}_{m1}$, $I^{u}_{m2}$ and $I^{v}_{m2}$ during his illness period, at the beginning of the epidemic, respectively. $1/X_{1}$, $1/X_{2}$, $1/X_{3}$, $1/X_{4}$, $1/X_{5}$, $1/X_{6}$, $1/X_{7}$ and $1/X_{8}$ represent the average duration in compartments $I^{u}_{f1}$, $L^{u}_{f1}$, $H^{u}_{f1}$, $C^{u}_{f1}$, $I^{v}_{f1}$, $L^{v}_{f1}$, $H^{v}_{f1}$ and $C^{v}_{f1}$, respectively. $1/M_{1}$, $1/M_{2}$, $1/M_{3}$, $1/M_{4}$, $1/M_{5}$, $1/M_{6}$, $1/M_{7}$ and $1/M_{8}$ represent the average duration in compartments $I^{u}_{f2}$, $L^{u}_{f2}$, $H^{u}_{f2}$, $C^{u}_{f2}$, $I^{v}_{f2}$, $L^{v}_{f2}$, $H^{v}_{f2}$ and $C^{v}_{f2}$, respectively. $(1-r_{f1}^{u})\rho /X_{1}$, $(1-r_{f1}^{v})\rho /X_{5}$, $(1-r_{f2}^{u})\rho /M_{1}$ and $(1-r_{f2}^{v})\rho /M_{5}$ are the probability that individuals in compartments $I^{u}_{f1}$, $I^{v}_{f1}$, $I^{u}_{f2}$ and $I^{v}_{f2}$ transfer to compartments $L^{u}_{f1}$, $L^{v}_{f1}$, $L^{u}_{f2}$ and $L^{v}_{f2}$, respectively. $(1-e_{f1}^{u})\eta /X_{2}$, $(1-e_{f1}^{v})\eta /X_{6}$, $(1-e_{f2}^{u})\eta /M_{2}$ and $(1-e_{f2}^{v})\eta /M_{6}$ are the probability that individuals in compartments $L^{u}_{f1}$, $L^{v}_{f1}$, $L^{u}_{f2}$ and $L^{v}_{f2}$ transfer to compartments $H^{u}_{f1}$, $H^{v}_{f1}$, $H^{u}_{f2}$ and $H^{v}_{f2}$, respectively. $\xi ^{u}_{f1}/X_{3}$, $\xi ^{v}_{f1}/X_{7}$, $\xi ^{u}_{f2}/M_{3}$ and $\xi ^{v}_{f2}/M_{7}$ are the probability that individuals in compartments $H^{u}_{f1}$, $H^{v}_{f1}$, $H^{u}_{f2}$ and $H^{v}_{f2}$ transfer to compartments $C^{u}_{f1}$, $C^{v}_{f1}$, $C^{u}_{f2}$ and $C^{v}_{f2}$, respectively. $\alpha _{f}/X_{1}$ and $\alpha _{f}/X_{5}$ are the probability that individuals in compartments $I^{u}_{f1}$ and $I^{v}_{f1}$ transfer to compartments $I^{u}_{f2}$ and $I^{v}_{f2}$, respectively. $u_{51}$, $u_{52}$, $u_{53}$ and $u_{54}$ represent the number of males in compartment $S^{u}_{m1}$ infected by a female belonging to compartments $I^{u}_{f1}$, $I^{v}_{f1}$, $I^{u}_{f2}$ and $I^{v}_{f2}$ during her illness period, at the beginning of the epidemic, respectively. $u_{61}$, $u_{62}$, $u_{63}$ and $u_{64}$ represent the number of males in compartment $S^{v}_{m1}$ infected by a female belonging to compartments $I^{u}_{f1}$, $I^{v}_{f1}$, $I^{u}_{f2}$ and $I^{v}_{f2}$ during her illness period, at the beginning of the epidemic, respectively. $u_{71}$, $u_{72}$, $u_{73}$ and $u_{74}$ represent the number of males in compartment $S^{u}_{m2}$ infected by a female belonging to compartments $I^{u}_{f1}$, $I^{v}_{f1}$, $I^{u}_{f2}$ and $I^{v}_{f2}$ during her illness period, at the beginning of the epidemic, respectively. $u_{81}$, $u_{82}$, $u_{83}$ and $u_{84}$ represent the number of males in compartment $S^{v}_{m2}$ infected by a female belonging to compartments $I^{u}_{f1}$, $I^{v}_{f1}$, $I^{u}_{f2}$ and $I^{v}_{f2}$ during her illness period, at the beginning of the epidemic, respectively.

The following results illustrate that the basic reproduction number is the key threshold for judging whether the disease persists.

#### Theorem 2

*When* $\mathcal {R}_{0}<1$, *the disease-free equilibrium* $\bar{E}$ *is globally asymptotically stable. When* $\mathcal {R}_{0}>1$, $\bar{E}$ *is unstable*.

Define $\mathbb {D}_{1}=\left\{ x\in \mathbb {D} : \sum _{\tilde{y}\in \textrm{Y}}\tilde{y}>0 \right\}$ and $\mathbb {D}_{2}=\left\{ x\in \mathbb {D} : \tilde{y}=0, \forall \tilde{y}\in \textrm{Y} \right\}$, where

$$\begin{aligned} \textrm{Y}=\{I^{u}_{f1}, L^{u}_{f1}, H^{u}_{f1}, C^{u}_{f1}, I^{v}_{f1}, L^{v}_{f1}, H^{v}_{f1}, C^{v}_{f1}, I^{u}_{f2}, L^{u}_{f2}, H^{u}_{f2}, C^{u}_{f2}, I^{v}_{f2}, L^{v}_{f2}, H^{v}_{f2}, C^{v}_{f2}, I^{u}_{m1}, I^{v}_{m1}, I^{u}_{m2}, I^{v}_{m2}\}. \end{aligned}$$

#### Theorem 3

*If* $\mathcal {R}_{0}>1$, *system* (1)–(4) *is uniformly persistent, that is, for any solution of system* (1)–(4) *with initial value* $x(0)\in \mathbb {D}_{1}$, *there is a positive constant* *l*, *such that*

$$\begin{aligned} \liminf \limits _{t\rightarrow +\infty }I^{g}_{fi}(t)\ge l,\ \liminf \limits _{t\rightarrow +\infty }L^{g}_{fi}(t)\ge l,\ \liminf \limits _{t\rightarrow +\infty }H^{g}_{fi}(t)\ge l,\ \liminf \limits _{t\rightarrow +\infty }C^{g}_{fi}(t)\ge l,\ \liminf \limits _{t\rightarrow +\infty }I^{g}_{mi}(t)\ge l, \end{aligned}$$

*where* $$g\in \{u,v\}$$ *and* $$i\in \{1,2\}$$.

Theorems 2 and 3 show that if $\mathcal {R}_{0}$ is less than 1, the disease will be controlled. And, the proofs of these two theorems are given in Appendixes 2 and 3.

**Table 3:** Values and sources of known parameters.

Parameter	Baseline value	Range	Reference
$A_f(A_m)$	See Appendix 5	–	–
$\mu _{f1}, \mu _{m1}, \mu _{f2}, \mu _{m2}$	See Appendix 5	–	–
$\alpha _f(\alpha _m)$	See Appendix 5	–	–
$\sigma _{f1}^{u}, \sigma _{f2}^{u}$	See Appendix 5	–	–
$\delta$	0	–	HPV vaccine show good immune persistence and protective effect^27,29^.
$\varepsilon _{f1}, \varepsilon _{f2}, \varepsilon _{m1}, \varepsilon _{m2}$	0.8416	0.76–0.955	^27^
$\omega _{m1}^{u}$	1.4268	1.1852–1.7526	^30^
$\omega _{m2}^{u}$	1.5957	1.3937–1.7647	^30^
$\omega _{m1}^{v}$	1.7526	1.469–2	^31^
$\omega _{m2}^{v}$	1.7647	1.5792–2	^31^
$\rho$	0.5917	0.4927–0.7081	^11,32–34^
$r_{f1}^{u}$	0.8788	0.8417–0.893	^11,32–34^
$r_{f2}^{u}$	0.698	0.5833–0.8382	^11,32–34^
$r_{f1}^{v}$	1	0.95251–1	^31^
$r_{f2}^{v}$	1	0.87499–1	^31^
$\eta$	0.2405	0.2323–0.2488	^27,32,34^
$e_{f1}^{u}$	0.70	0.6776–0.7258	^27,32,34^
$e_{f2}^{u}$	0.455	0.4066–0.5064	^27,32,34^
$e_{f1}^{v}$	1	0.8675–1	^31^
$e_{f2}^{v}$	1	0.7479–1	^31^
$\xi _{f1}^{u}$	0.066	0.0539–0.07815	^32–35^
$\xi _{f2}^{u}$	0.085	0.0712–0.09885	^32–35^
$\xi _{f1}^{v},\ \xi _{f2}^{v}$	0	–	Precancerous lesions associated with HPV vaccine-immune virus types have not been found in HPV-vaccinated study subjects^27,36^.
$\Lambda$	0.3	0.225–0.375	^33^
$\gamma _{f1}, \gamma _{f2}$	0.9526	–	^17,27^
$\zeta _{f1}, \zeta _{f2}$	0.0395	0.0306–0.0529	^37,38^
$\zeta _{m1}, \zeta _{m2}$	0.0743	0.0403–0.1192	^37,38^

## Results

### Data fitting and uncertainty analysis

In this section, the cumulative cases and cumulative deaths of CC related to HPV16 and HPV18 from 2006 to 2016 (see Appendix 4) are fitted to system (1)–(4), and the unknown parameters and initial values are estimated at the same time. Since vaccines were launched in China at the end of 2017^14^, we did not consider compartments containing vaccinated individuals, that is, the variable superscripted as *v*, in the process of fitting the data. The unit of time is year. The values of the known parameters can be seen in Table 3. In order to reduce the estimated parameters, we rewrite

$$\begin{aligned} \beta _{mij}=\beta _{m}c_{mij},\ \beta _{fij}=\beta _{f}c_{fij},\ i,j\in \{1,2\} \end{aligned}$$

and then estimate transmission coefficients $\beta _{mij}$ and $\beta _{fij}$ ($i,j\in \{1,2\}$). As can be seen from Fig. S2 in Appendix 5, the CC screening rate gradually increased from 2006 to 2013, and remained between 0.18 and 0.2 after 2014. In addition, with the increase in screening rate, individuals in $D_{f1}$ and $D_{f2}$ have received timely treatment, and mortality rates have decreased accordingly. Therefore, suppose that mortality coefficients $d_{f1}$ and $d_{f2}$ change over time:

$$\begin{aligned} d_{f1}=d_{k1}\textrm{e}^{-d_{b1}t},\ d_{f2}=d_{k2}\textrm{e}^{-d_{b2}t}, \end{aligned}$$

where $d_{k1}$, $d_{b1}$, $d_{k2}$ and $d_{b2}$ are all greater than 0 and are also parameters to be estimated. For initial values, only $C^{u}_{f1}(0)$ and $C^{u}_{f2}(0)$ need to be estimated (see Appendix 6).

**Fig. 4:**
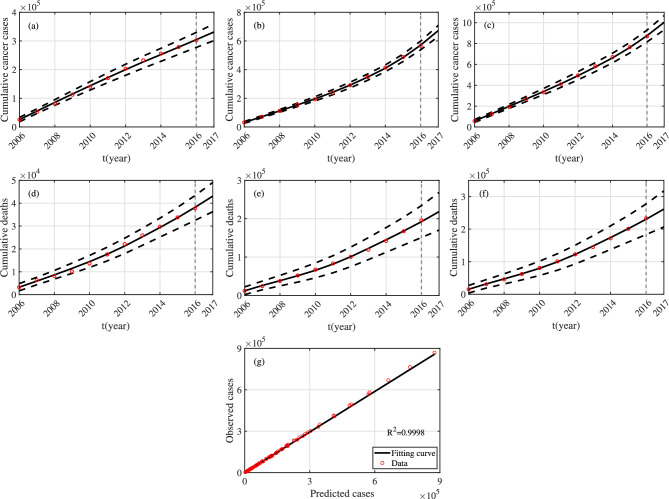
Fitting results regarding the number of HPV16 and HPV18 related CC cases and deaths from 2006 to 2016. The red circles represent the actual cumulative number of CC cases or deaths. The black solid line represents the fitting result, and the black dashed line represents the 95% confidence interval. (**a**) Cumulative cases in the first age group. (**b**) Cumulative cases in the second age group. (**c**) The sum of cumulative cases in the two age groups. (**d**) Cumulative deaths in the first age group. (**e**) Cumulative deaths in the second age group. (**f**) The sum of cumulative deaths in the two age groups. (**g**) Relation between observed and predicted cases.

**Fig. 5:**
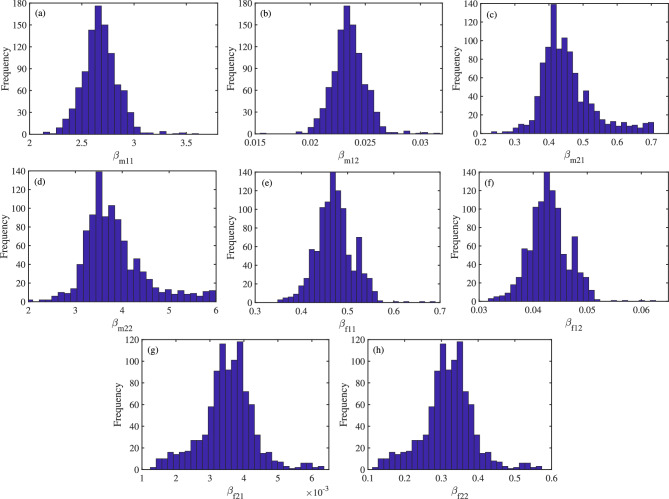
For HPV16 and HPV18, histogram of 1000 values for parameters $\beta _{m11}$, $\beta _{m12}$, $\beta _{m21}$, $\beta _{m22}$, $\beta _{f11}$, $\beta _{f12}$, $\beta _{f21}$ and $\beta _{f22}$.

**Fig. 6:**
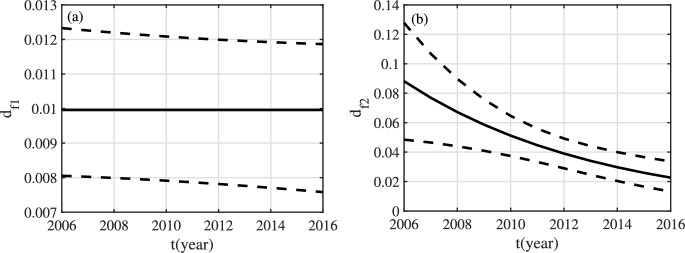
For HPV16 and HPV18, estimation results about $d_{f1}$ and $d_{f2}$. The black solid line represents the fitting result, and the black dashed line represents the $95\%$ confidence interval.

**Fig. 7:**
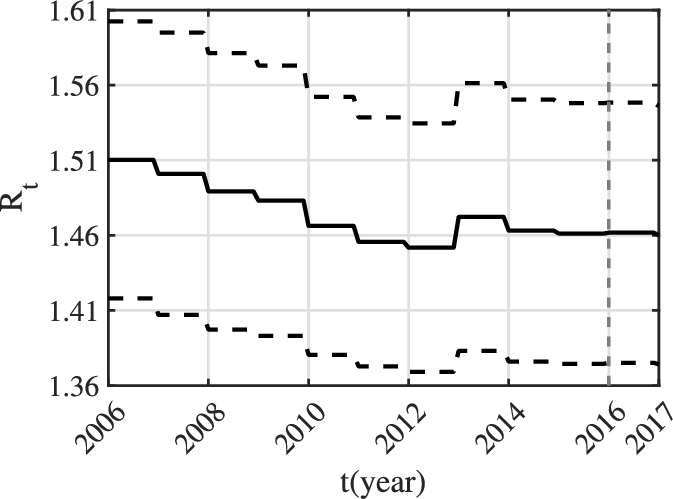
For HPV16 and HPV18, time-varying reproduction number. The black solid line represents the fitting result, and the black dashed line represents the $95\%$ confidence interval.

**Table 4:** For HPV16 and HPV18, means and $95\%$ confidence interval of the estimated parameters and initial values.

Parameter	Value	$95\%$ CI	Parameter	Value	$95\%$ CI
$\beta _{m11}$	2.6929	[2.3428, 3.0083]	$\beta _{m12}$	0.0236	[0.0206, 0.0264]
$\beta _{m21}$	0.4942	[0.3019, 0.6052]	$\beta _{m22}$	4.1855	[2.5573, 5.1258]
$\beta _{f11}$	0.4709	[0.3939, 0.5509]	$\beta _{f12}$	0.0428	[0.0358, 0.0501]
$\beta _{f21}$	0.003	[0.002, 0.005]	$\beta _{f22}$	0.2749	[0.181, 0.4547]
$d_{k1}$	0.01	[0.0081, 0.0123]	$d_{b1}$	$2.8786\times 10^{-8}$	$[3.1687\times 10^{-14},0.01]$
$d_{k2}$	0.0882	[0.05, 0.12]	$d_{b2}$	0.1359	[0.0559, 0.2086]
$C^{u}_{f1}(0)$	$7.9321\times 10^{4}$	$[6.8089\times 10^{4}, 8.5087\times 10^{4}]$	$C^{u}_{f2}(0)$	$1.0618\times 10^{5}$	$[9.4087\times 10^{4}, 1.1154\times 10^{5}]$

The data is fitted using the least square method. We first give the following definitions: (1) $\tilde{Y}_{ci}(t)$ ($Y_{ci}(t)$) denotes predicted (observed) cumulative confirmed cases of CC in the *i-*th age group in year *t* ($i\in \{1,2\}$); (2) $\tilde{Y}_{di}(t)$ ($Y_{di}(t)$) denotes predicted (observed) cumulative deaths of CC in the *i-*th age group in year *t* ($i\in \{1,2\}$); (3) $\tilde{Y}_{c3}(t)$ ($Y_{c3}(t)$) denotes predicted (observed) value of the total cumulative confirmed cases of CC in the two age groups in year *t*; (4) $\tilde{Y}_{d3}(t)$ ($Y_{d3}(t)$) denotes predicted (observed) value of the total cumulative deaths of CC in the two age groups in year *t*. From the content of Section 2, the following equations hold:

$$\begin{aligned} \frac{\textrm{d}\tilde{Y}_{c1}}{dt}&=[\Lambda +(1-\Lambda )\sigma ^{u}_{f1}]C^{u}_{f1},&\quad \frac{\textrm{d}\tilde{Y}_{d1}}{dt}&=d_{f1}D_{f1}\\ \frac{\textrm{d}\tilde{Y}_{c2}}{dt}&=[\Lambda +(1-\Lambda )\sigma ^{u}_{f2}]C^{u}_{f2},&\frac{\textrm{d}\tilde{Y}_{d2}}{dt}&=d_{f2}D_{f2},\\ \frac{\textrm{d}\tilde{Y}_{c3}}{dt}&=[\Lambda +(1-\Lambda )\sigma ^{u}_{f1}]C^{u}_{f1} +[\Lambda +(1-\Lambda )\sigma ^{u}_{f2}]C^{u}_{f2},&\frac{\textrm{d}\tilde{Y}_{d3}}{dt}&=d_{f1}D_{f1}+d_{f2}D_{f2},\\ \end{aligned}$$

The least squares method is to find the appropriate parameters and initial values that need to be estimated, so that

$$\begin{aligned} F=\sum _{i=1}^{3}\sum _{t=2006}^{2016} [(\tilde{Y}_{ci}(t)-Y_{ci}(t))^2+(\tilde{Y}_{di}(t)-Y_{di}(t))^2] \end{aligned}$$

reaches the minimum. In order to evaluate the quality of the fitting results, we also need to perform linear regression between the predicted cases and the observed cases, and calculate the corresponding determinate coefficient $R^2$. The larger $R^2$ is, the more interpretable the fitted values are to the actual values. In the fitting process, the parameters $A_f$, $A_m$, $\mu _{fi}$, $\mu _{mi}$, $\alpha _f$, $\alpha _m$, $\sigma _{fi}^{u}$ and $d_{fi}$ ($i\in \{1,2\}$) are time varying. After estimating all unknown parameters, we can calculate the time-varying reproduction number

$$\begin{aligned} \mathcal {R}_{t}=\mathcal {R}_{0}(A_f(t), A_m(t), \mu _{f1}(t), \mu _{f2}(t), \mu _{m1}(t), \mu _{m2}(t), \alpha _f(t), \alpha _m(t), \sigma _{f1}^{u}(t), \sigma _{f2}^{u}(t), d_{f1}(t), d_{f2}(t)). \end{aligned}$$

We fit the following three sets of data: (1) the number of CC cases and deaths related to HPV16 and HPV18; (2) the data related to HPV16 only; (3) the data related to HPV18 only. The fitting results corresponding to the first set of data can be seen in Figs. 4, 6 and 7 and Table 4; the fitting results corresponding to the second set of data can be seen in can be seen in Figs. S3, S5 and S6 and Table S2 (see Appendix 7); the fitting results corresponding to the third set of data can be seen in Figs. S7, S9 and S10 and Table S3 (see Appendix 7). From Figs. 4g, S3g and S7g, it can be seen that the $R^2$ corresponding to the three cases is equal to 0.9998 and close to 1. Hence, the fitting results are strongly interpretable to the observed values. It can be seen from Figs. 6, S5 and S9 that the mortality rate of the first age group remains basically unchanged, while the mortality rate of the second age group decreases year by year. From Figs. 7, S6 and S10, it can be known that for HPV16 and HPV18, range of $\mathcal {R}_{t}$ from 2006 to 2016 is [1.452, 1.51]; for HPV16, range of $\mathcal {R}_{t}$ from is [1.415, 1.472]; for HPV18, range of $\mathcal {R}_{t}$ is [1.255, 1.306]. Clearly, all $\mathcal {R}_{t}$ are greater than 1 and the disease will persist if control measures are not taken. And, $\mathcal {R}_{t}$ for HPV16 is larger than $\mathcal {R}_{t}$ for HPV18.

The number of CC cases and deaths across the country is predicted by data from multiple local cancer registries^39^, but the cancer registries do not cover all populations. Hence, there are certain errors in the final data. We quantify the uncertainty of the parameters and construct confidence intervals by assuming an error structure^40^. The specific process is as follows:

(1) The first step is to generate 1000 groups of samples about the number of cases and deaths. We take the number of cases in the first age group as an example to introduce how the samples are generated. The predicted number of new cases in year *t* is $\bar{Y}_{c1}(t)=\tilde{Y}_{c1}(t)-\tilde{Y}_{c1}(t-1)$, where $t=2007,2008,\cdots ,2016$, and $\bar{Y}_{c1}(2006)=\tilde{Y}_{c1}(2006)$. The observed value of new cases in year *t* is $\hat{Y}_{c1}(t)=Y_{c1}(t)-Y_{c1}(t-1)$, where $t=2007,2008,\cdots ,2016$, and $\hat{Y}_{c1}(2006)=Y_{c1}(2006)$. The ratio between the square of the residual $(\hat{Y}_{c1}(t)-\bar{Y}_{c1}(t))$ and $\bar{Y}_{c1}(t)$ is $n_t=(\hat{Y}_{c1}(t)-\bar{Y}_{c1}(t))^2/\bar{Y}_{c1}(t)$, $t=2006,2007,\cdots ,2016$. Hence, we assume that observations of new cases at time *t* obeys a negative binomial distribution with mean $\bar{Y}_{c1}(t)$ and variance $n\bar{Y}_{c1}(t)$, where

$$\begin{aligned} n=\frac{\max \left\{ n_t : t=2006,2007,\ldots ,2016\right\} +\sum _{t=2006}^{2016}n_t\big /11}{2}. \end{aligned}$$

Then, we randomly take 1000 values from the negative binomial distribution to obtain 1000 samples of new cases in year *t*, denoted $\hat{Y}_{c1}(t,j)$, where $j = 1,2,\ldots ,1000$. Finally, through simple summation calculation, 1000 samples of accumulated cases in year *t* can be directly obtained, which are denoted as $Y_{c1}(t,j)$, where $j = 1,2,\ldots ,1000$. Therefore, we get 1000 groups of time series samples.

(2) Each group of samples is fitted using the least square method, and 1000 fitting results and 1000 estimated parameter values and initial values are obtained. The histograms of 1000 values for parameters $\beta _{mij}$ and $\beta _{fij}$ ($i,j\in \{1,2\}$) can be seen in Figs. 5, S4 and S8 (see Appendix 7).

(3) According to 1000 sets of parameter values, 1000 sets of $\mathcal {R}_{t}$ can be obtained. Assume that the fitting results, the estimated parameter values and initial values, and $\mathcal {R}_{t}$ obey the normal distribution. And, the corresponding $95\%$ confidence interval is obtained, which can be seen in Figs. 4, 6 and 7, and Table 4, as well as Figs. S3, S5, S6, S7, S9 and S10 and Tables S2 and S3 (see Appendix 7).

(4) The HPV vaccine was first introduced in China in late 2017^14^. In the following, we will take the prevalence of HPV in 2017 as the initial value to assess the vaccination strategy. But, due to the lag of data from the National Cancer Center in China, the incidence and mortality of CC in 2017 have not yet been released. Hence, based on the fitting results of the number of CC cases and deaths related to HPV16 and HPV18 from 2006 to 2016, we predict the cumulative number of cases and deaths in 2017, and the results can be seen in Fig. 4.

**Fig. 8:**
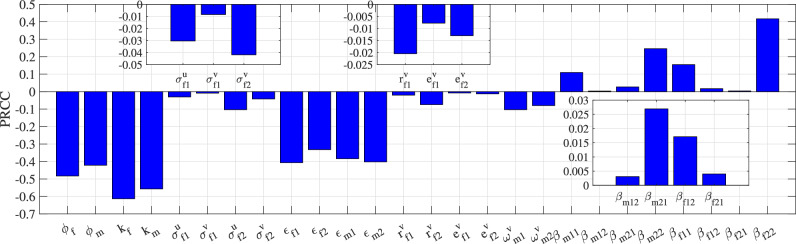
Partial rank correlation coefficient of parameters with respect to $\mathcal {R}_{0}$.

### Optimal control problem

The main measures to control CC are vaccination and CC screening. The WHO is calling for $90\%$ of girls to be vaccinated by the age of 15^41^. In 2002, China included hepatitis B vaccine in Expanded Program on Immunization, and the coverage rate of hepatitis B vaccine was eventually maintained at around 95%^42^. Therefore, assuming that $0\le \phi _{se}\le 0.95$ and $0\le k_{se}\le 0.96$, where $se\in \{f,m\}$. WHO’s recommendations for CC screening are regular screening every five to ten years starting at age 30^41^. Based on the alternative control strategies for CC in the reference^19^, we assume that women over the age of 30 are screened for CC at most every three years. Based on the study of CC screening rate in China in the references^43^ and^44^, we assume that the maximum CC screening rate for women aged 20–29 is 16% per year. From 2018 to 2100, the proportion of the population aged 30–44 in the first female age group is about 54.74%^45^, and the proportion of the population aged 20–29 in the first female age group is about 30.98%. According to the above actual situation, we get

$$\begin{aligned} 0\le \sigma ^g_{f2}(t)\le 1/3\ \text {and} \ 0\le \sigma ^g_{f1}(t)\le \frac{0.5474}{3}+0.3098\times 0.16=0.232,\ \ \text {where} \ g\in \{u,v\}. \end{aligned}$$

We subsequently employed Partial Rank Correlation Coefficient (PRCC) analysis^46^ to quantify the sensitivity of key epidemiological parameters influencing the basic reproduction number ($\mathcal {R}_{0}$). The PRCC metric, bounded between $-1$ and 1, provides a robust measure of parameter influence. PRCC interpretation: $\rightarrow +1$ (strong positive), $\rightarrow -1$ (strong negative), $\approx 0$ (no significant correlation). It can be seen from Fig. 8 that the parameters $k_f$, $k_m$, $\phi _f$ and $\phi _m$ are negatively correlated with $\mathcal {R}_{0}$, and the correlation is the strongest. Therefore, vaccination can effectively control the spread of HPV. The protective efficacy $\varepsilon$ of vaccine also has a relatively large influence on $\mathcal {R}_{0}$. For the parameters $\beta _{mij}$ and $\beta _{fij}$ ($i,j\in \{1,2\}$), $\beta _{f22}$ has the strongest correlation with $\mathcal {R}_{0}$.

**Fig. 9:**
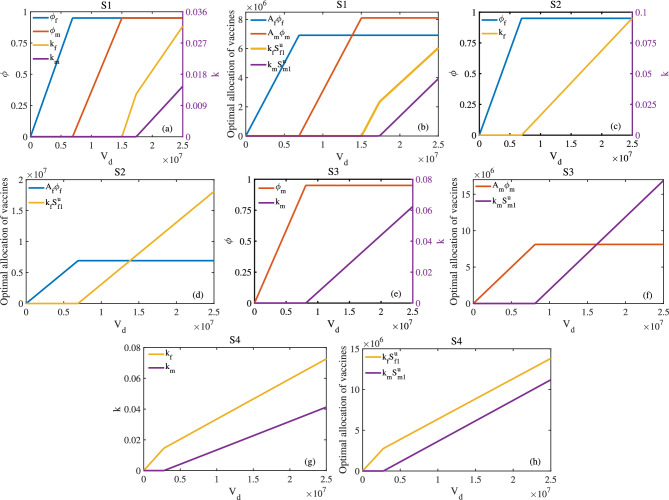
In the case of limited vaccine resources, the optimal vaccine allocation corresponding to the four different strategies and the optimal values of the corresponding parameters $\phi _f$, $\phi _m$, $k_f$ and $k_m$.

**Fig. 10:**
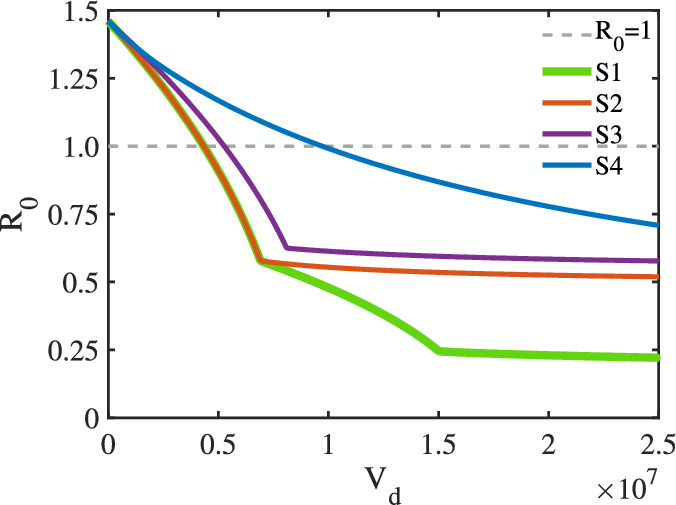
Basic reproduction number for four different strategies in the case of limited vaccine resources.

#### Limited vaccine production

China currently faces a substantial gap between HPV vaccine supply and population demand. With approximately 320 million females aged 9–45 years nationwide, the cumulative vaccination coverage reached merely 3.17% during the 2018–2020 period^14^. This severe supply constraint necessitates optimal vaccine allocation strategies to maximize epidemiological impact. We therefore formulate the following resource-constrained optimization problem:

5 $$\begin{aligned} \text {minimize}\quad \quad&\mathcal {R}_{0} \\ \text {subject to}\quad \quad&\phi _{f},\phi _{m}\in [0, 0.95]\quad \text {and} \quad k_{f},k_{m}\in [0, 0.96] \nonumber \\&\phi _{f}A_f+\phi _{m}A_m+k_{f}S^{u}_{f1}+k_{m}S^{u}_{m1}=V_d,\nonumber \end{aligned}$$

where $V_d$ represents the fixed vaccine amount.

Assume that $\sigma ^{u}_{f1}=\sigma ^{v}_{f1}$ and $\sigma ^{u}_{f2}=\sigma ^{v}_{f2}$. The values of other known parameters included in $\mathcal {R}_{0}$ are shown in Tables 3 and 4. $S^{u}_{f1}$ ($S^{u}_{m1}$) represents the number of unvaccinated susceptible individuals in the first age group of females (males) at the end of 2017. Using the established parameter values, we evaluate four distinct vaccine allocation strategies:

- Strategy S1 (Gender- and age-inclusive vaccination):
  - Target population: Both males and females across all eligible ages
  - Age restriction: Unrestricted by age cohort
- Strategy S2 (Female-only vaccination):
  - Target population: Females across all eligible ages
  - Implementation: Unrestricted by age cohort
- Strategy S3 (Male-only vaccination):
  - Target population: Males across all eligible ages
  - Implementation: Unrestricted by age cohort
- Strategy S4 (Gender-inclusive young adult vaccination):
  - Target population: Both males and females
  - Age restriction: Limited to the first age cohort (15–44 years)

We numerically solved the optimization problem using MATLAB’s *fmincon* function. The simulation results (Figs. 9, 10) reveal several key findings: (1) S1 (gender- and age-inclusive vaccination) achieved the minimal $\mathcal {R}_{0}$, representing the optimal strategy, and S4 (gender-inclusive young adult vaccination) yielded the maximal $\mathcal {R}_{0}$, demonstrating the least effective approach. (2) Primary vaccine allocation should prioritize 14-year-old girls and boys in S1–S3 strategies. For S1, subsequent vaccine allocation should be extended to females (15–44 years) when $V_d>1.5017\times 10^7$, and further to males (15–44 years) when $V_d>1.74\times 10^7$. (3) Female-only vaccination (S2) outperformed male-only vaccination (S3) in transmission reduction. (4) Annual vaccination of all 14-year-old girls reduces $\mathcal {R}_{0}$ to 0.58, and including all 14-year-old boys further reduces it to 0.244.

#### Control cost minimization

To determine optimal control strategies that balance economic costs with disease control effectiveness, we incorporate both vaccination and CC screening interventions into our dynamic model. The vaccination parameters are extended to time-dependent functions, where $\phi _f(t)$ and $\phi _m(t)$ represent the time-varying vaccination coverage rates for 14-year-old girls and boys respectively, while $k_f(t)$ and $k_m(t)$ denote the time-varying vaccination rates for females and males aged 15–44 years respectively. Similarly, the CC screening rates are generalized to time-varying functions $\sigma ^u_{f1}(t)$, $\sigma ^v_{f1}(t)$ for unvaccinated and vaccinated females aged 15–44, and $\sigma ^u_{f2}(t)$, $\sigma ^v_{f2}(t)$ for the corresponding groups aged 45 years and above, allowing for dynamic adjustment of screening strategies over time. Denote

$$\begin{aligned} {\mathbf {u}}=[{\phi _f(t),k_f(t),\sigma ^u_{f1}(t),\sigma ^v_{f1}(t),\sigma ^u_{f2}(t), \sigma ^v_{f2}(t),\phi _m(t),k_m(t)}]. \end{aligned}$$

Its feasible decision space is

$$\begin{aligned} U=\{&{\mathbf {u}}|0\le \phi _{se}(t)\le 0.95, 0\le k_{se}(t)\le 0.96, 0\le \sigma ^g_{f1}(t)\le 0.232, 0\le \sigma ^g_{f2}(t)\le 1/3 \ \text {for}\ 0\le t\le T,\\ &\phi _{se}(t), k_{se}(t) \ \text {and}\ \sigma ^g_{fi}(t)\ \text {are Lebesgue measurable}, \ \text {where}\ se\in \{f,m\}, g\in \{u,v\} \ \text {and}\ i\in \{1,2\}\}. \end{aligned}$$

In the time period [0, *T*], the cost of vaccination, the cost of CC screening and the cost of treatment are

$$\begin{aligned} Cost_{va}&=\int _{0}^{T}B_1(A_f\phi _{f}(t)+A_m\phi _{m}(t))+B_2(k_f(t)S^{u}_{f1}(t)+k_m(t)S^{u}_{m1}(t))\textrm{d}t,\\ Cost_{sc}&=\int _{0}^{T}\sum _{\begin{array}{c} g\in \{u,v\} \\ i\in \{1,2\} \end{array}}E_1\{\sigma ^{g}_{fi}(t)(L^g_{fi}(t)+H^{g}_{fi}(t))+[\Lambda +(1-\Lambda )\sigma ^{g}_{fi}(t)]C^{g}_{fi}(t)\}\\&\quad +\sum _{i\in \{1,2\}}E_2[\sigma ^{u}_{fi}(t)(S^u_{fi}(t)+I^{u}_{fi}(t)+R_{fi})+\sigma ^{v}_{fi}(t)(S^v_{fi}(t)+I^{v}_{fi}(t))]\textrm{d}t,\\ Cost_{tr}&=\int _{0}^{T}\sum _{\begin{array}{c} g\in \{u,v\}\\ i\in \{1,2\} \end{array}}\{T_{L}\sigma ^{g}_{fi}(t)L^g_{fi}(t)+T_{H}\sigma ^{g}_{fi}(t)H^{g}_{fi}(t)+T_{C}[\Lambda +(1-\Lambda )\sigma ^{g}_{fi}(t)]C^{g}_{fi}(t)\}\textrm{d}t, \end{aligned}$$

respectively. Here $A_f\phi _{f}(t)+A_m\phi _{m}(t)$ represents the newly vaccinated number of 14-year-old girls and boys at time *t*. $k_f(t)S^{u}_{f1}(t)+k_m(t)S^{u}_{m1}(t)$ represents the newly vaccinated number of males and females aged 15–45 at time *t*. $B_1$ and $B_2$ denote the average vaccination cost for an individual aged 14 and 15–45, respectively. $B_1$ is the sum of the price and service cost of two doses of the bivalent vaccine. $B_2$ is the sum of the price and service cost of three doses of the bivalent vaccine. Vaccine service cost includes storage and transportation of vaccine, publicity materials and advocacy work, medical staff training and salaries, etc^47^. $\sigma ^{g}_{fi}(t)(L^g_{fi}(t)+H^{g}_{fi}(t))+[\Lambda +(1-\Lambda )\sigma ^{g}_{fi}(t)]C^{g}_{fi}(t)$ represents the number of patients with LSIL, HSIL and CC who are screened at time *t*. $\sigma ^{u}_{fi}(t)(S^u_{fi}(t)+I^{u}_{fi}(t)+R_{fi})+\sigma ^{v}_{fi}(t)(S^v_{fi}(t)+I^{v}_{fi}(t))$ is the number of individuals in compartments $S^u_{fi}$, $I^{u}_{fi}$, $R_{fi}$, $S^v_{fi}$ and $I^{v}_{fi}$($i\in \{1,2\}$) undergoing screening at time *t*. $E_1$ represents the cost of screening a patient with LSIL, HSIL or CC. Specifically, $E_1$ includes the costs of gynecological examination, HPV testing, liquid-based cytology examination, colposcopy examination, and histopathological examination^33^. $E_2$ represents the cost of screening an uninfected individual or an individual belonging to the *I* compartment. And $E_2$ only includes the costs of gynecological examination, HPV testing, liquid-based cytology examination^33^. $T_{L}$, $T_{H}$ and $T_{C}$ represent the treatment costs of patients with LSIL, HSIL and CC, respectively. The treatment costs include direct treatment costs (such as hospitalization, medication treatments, physical therapies, chemotherapy, and surgeries, etc.) as well as indirect treatment costs (such as nutrition expenses, round-trip transportation fees, wage losses, etc.)^48^. In addition, we also need to control the number of CC patients at time *T* to near the target value $\mathbb {I}$, that is,

$$\begin{aligned} C_T=\left| C_{f1}^{u}(T)+C_{f1}^{v}(T)+C_{f2}^{u}(T)+C_{f2}^{v}(T)-\mathbb {I}\right| \end{aligned}$$

should be close to 0.

**Fig. 11:**
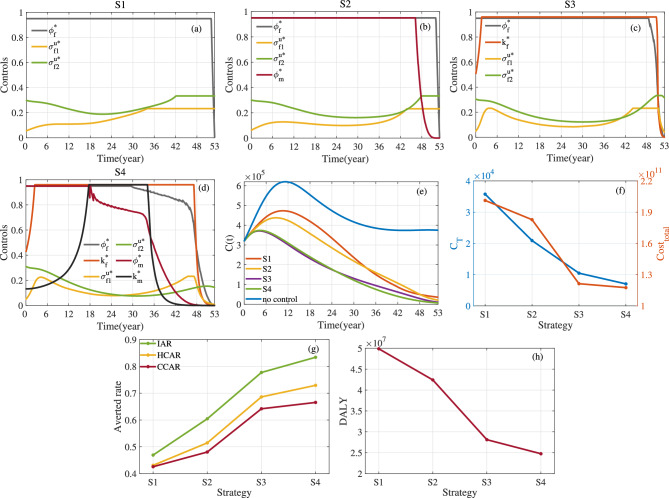
Optimal control and evaluation indicators for four control strategies. Here $a_1=1$, $a_2=10^{-10}$, $\kappa _1=\kappa _2=\kappa _3=1/3$ and $T=53$. (**a**) Optimal control for S1. (**b**) Optimal control for S2. (**c**) Optimal control for S3. (**d**) Optimal control for S4. (**e**) *C*(*t*). (**f**) $C_{T}$ and $Cost_{total}$. (**g**) Averted Rate. (**h**) *DALY*.

**Fig. 12:**
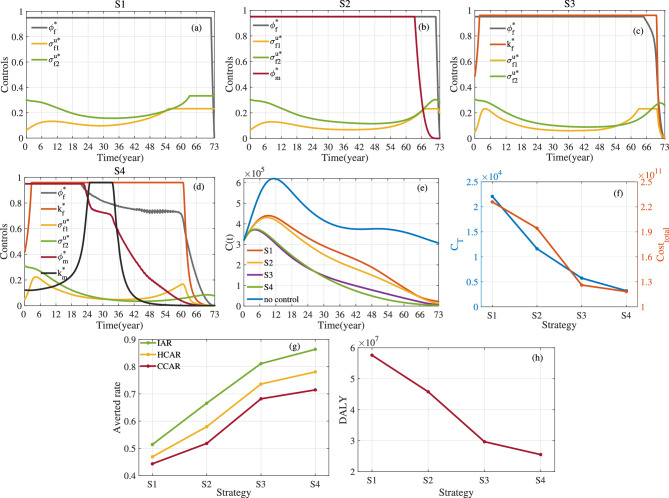
Optimal control and evaluation indicators for four control strategies. Here $a_1=1$, $a_2=10^{-10}$, $\kappa _1=\kappa _2=\kappa _3=1/3$ and $T=73$. (**a**) Optimal control for S1. (**b**) Optimal control for S2. (**c**) Optimal control for S3. (**d**) Optimal control for S4. (**e**) *C*(*t*). (**f**) $C_{T}$ and $Cost_{total}$. (**g**) Averted Rate. (**h**) *DALY*.

**Fig. 13:**
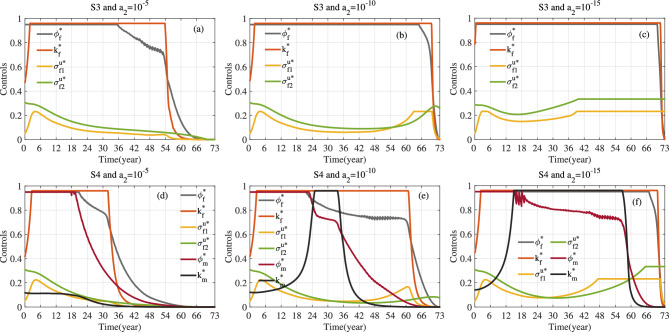
For S3 and S4, optimal control corresponding to different values of weight parameter $a_2$. Here $a_1=1$, $\kappa _1=\kappa _2=\kappa _3=1/3$ and $T=73$.

**Fig. 14:**
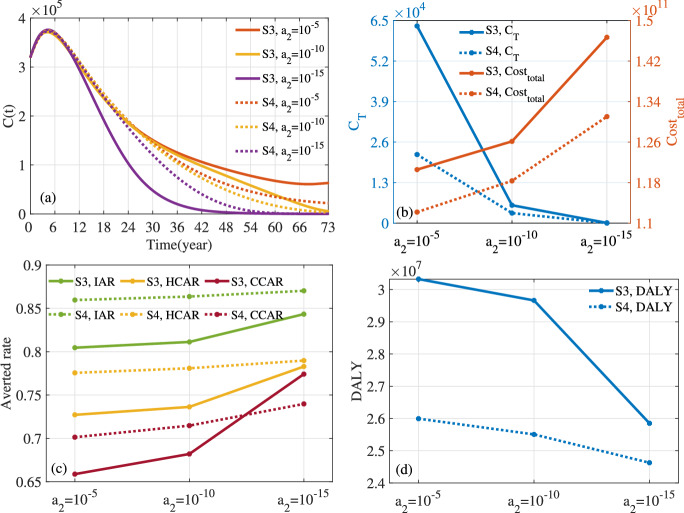
For S3 and S4, evaluation indicators of optimal control corresponding to different values of weight parameter $a_2$. (**a**) *C*(*t*). (**b**) $C_{T}$ and $Cost_{total}$. (**c**) Averted Rate. (**d**) *DALY*.

**Fig. 15:**
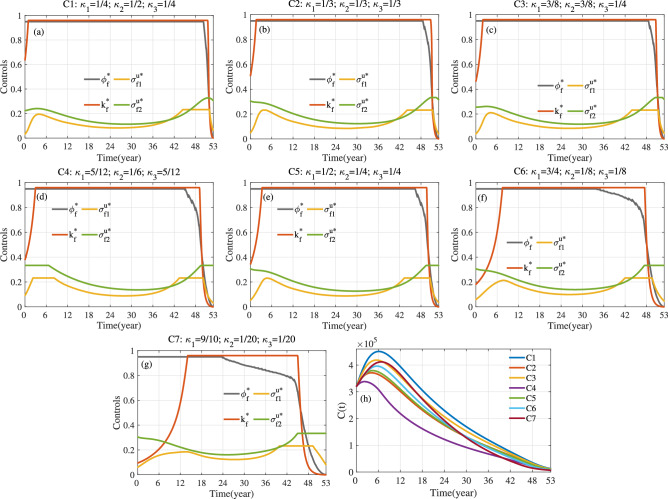
For S3, optimal control and *C*(*t*) corresponding to different values of weight parameters $\kappa _1$, $\kappa _2$ and $\kappa _3$. Here $a_1=1$, $a_2=10^{-10}$ and $T=53.$.

**Fig. 16:**
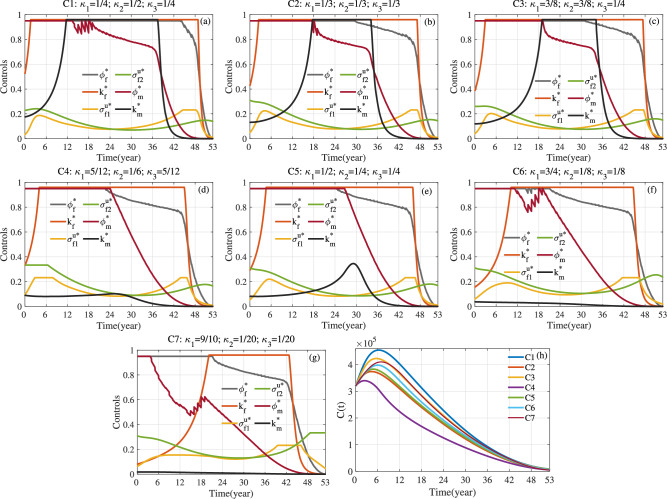
For S4, optimal control and *C*(*t*) corresponding to different values of weight parameters $\kappa _1$, $\kappa _2$ and $\kappa _3$. Here $a_1=1$, $a_2=10^{-10}$ and $T=53$.

**Fig. 17:**
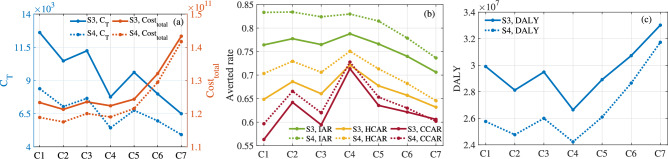
For S3 and S4, evaluation indicators of optimal control corresponding to different values of weight parameters $\kappa _1$, $\kappa _2$ and $\kappa _3$. (**a**) $C_{T}$ and $Cost_{total}$. (**b**) Averted Rate. (**c**) *DALY*.

**Fig. 18:**
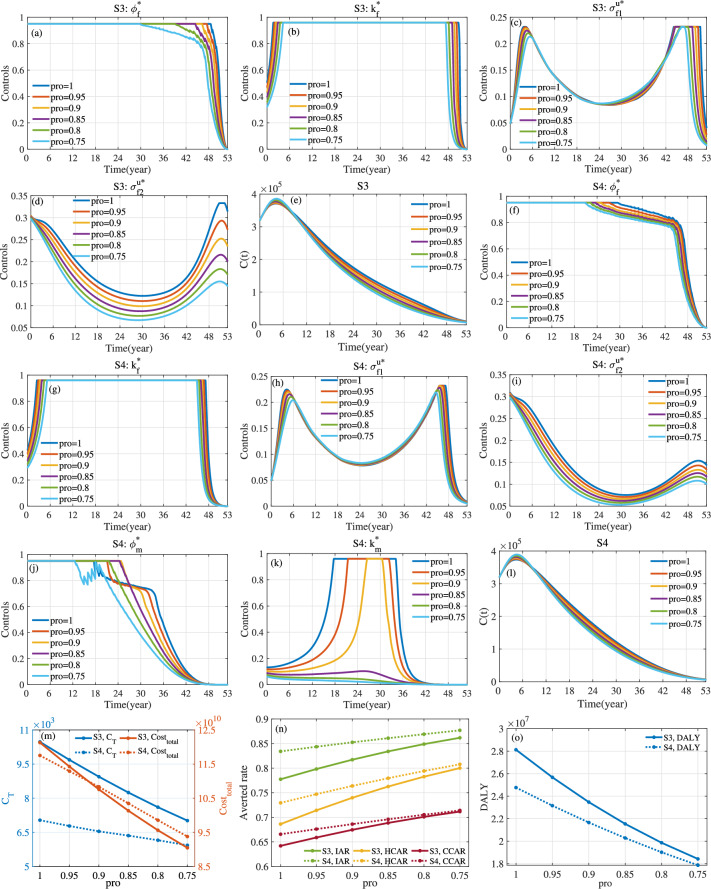
For S3 and S4, optimal control and evaluation indicators with different values of parameter *pro*. Here $a_1=1$, $a_2=10^{-10}$, $\kappa _1=\kappa _2=\kappa _3=1/3$ and $T=53$.

**Fig. 19:**
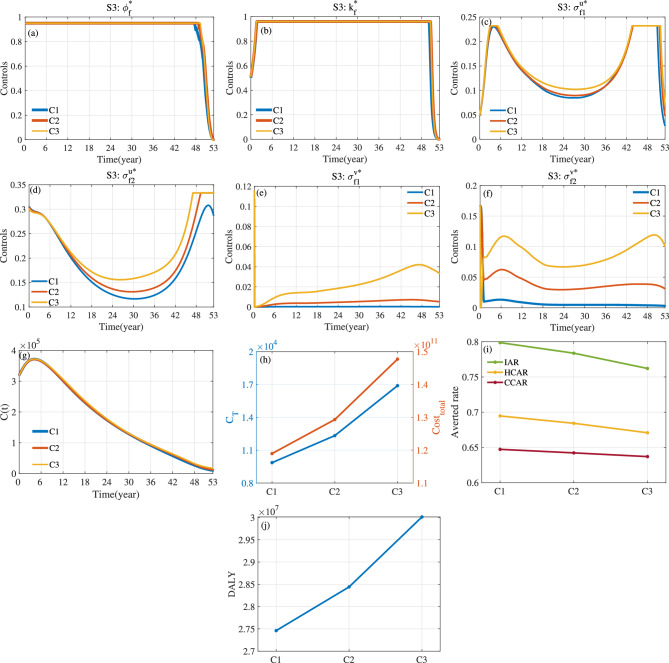
For S3, optimal control and evaluation indicators with different values of parameters $\varepsilon _{fi}$, $\varepsilon _{mi}$, $r^v_{fi}, e^v_{fi}$ and $\xi ^v_{fi}$ ($i\in \{1,2\}$). Here $a_1=1$, $a_2=10^{-10}$, $\kappa _1=\kappa _2=\kappa _3=1/3$ and $T=53$.

Now, we study the following multi-objective optimization problem

$$\begin{aligned} \min _{{\mathbf {u}}\in U}Cost_{va},\ \min _{{\mathbf {u}}\in U}Cost_{sc}, \ \min _{{\mathbf {u}}\in U}Cost_{tr}\ \text {and}\ \min _{{\mathbf {u}}\in U}C_T \end{aligned}$$

subject to system (S8.1)–(S8.5) (see Appendix 8). Then, by the weighted global criterion method^49^, we integrate the multi-objective function as a single-objective scalar composite function and further transform the above multi-objective optimization problem into the following optimization problem:

6 $$\begin{aligned} \min _{{\mathbf {u}}\in U} J({\mathbf {u}}):= \phi (e)+\int _{0}^{T}\Phi (t,x,{\mathbf {u}})\textrm{d}t, \end{aligned}$$

subject to system (S8.1)–(S8.5) (see Appendix 8) with

$$\begin{aligned} e=(0,T,x(0),x(T))\in \mathcal {S}\subset \mathbb {R}^{2+2\times 34}, \end{aligned}$$

where

$$\begin{aligned} \phi (e)=&\frac{a_1}{2}C_{T}^2,\\ \Phi (t,x,{\mathbf {u}})=&\frac{a_2}{2}\kappa _{1}\sum _{se\in \{f,m\}}\left[ (B_1A_{se}\phi _{se}(t))^2 +(B_2k_{se}(t)S^{u}_{se1}(t))^2\right] \\&+\frac{a_2}{2}\kappa _{2}\sum _{\begin{array}{c} g\in \{u,v\} \\ i\in \{1,2\} \end{array}} \left\{ \left( E_1\sigma ^{g}_{fi}(t)L^g_{fi}(t)\right) ^2+\left( E_1\sigma ^{g}_{fi}(t)H^g_{fi}(t)\right) ^2 +\left[ E_1\left( \Lambda +(1-\Lambda )\sigma ^{g}_{fi}(t)\right) C^{g}_{fi}(t)\right] ^2\right\} \\&+\frac{a_2}{2}\kappa _{2}\sum _{i\in \{1,2\}}\left\{ [E_2\sigma ^{u}_{fi}(t)(S^u_{fi}(t)+I^{u}_{fi}(t)+R_{fi})]^2 +[E_2\sigma ^{v}_{fi}(t)(S^v_{fi}(t)+I^{v}_{fi}(t))]^2\right\} \\&+\frac{a_2}{2}\kappa _{3}\sum _{\begin{array}{c} g\in \{u,v\} \\ i\in \{1,2\} \end{array}} \left\{ \left( T_{L}\sigma ^{g}_{fi}(t)L^g_{fi}(t)\right) ^2 +\left( T_{H}\sigma ^{g}_{fi}(t)H^{g}_{fi}(t)\right) ^2+\left[ T_{C}\left( \Lambda +(1-\Lambda ) \sigma ^{g}_{fi}(t)\right) C^{g}_{fi}(t)\right] ^2\right\} . \end{aligned}$$

Here, $\sum _{i=1}^{3}\kappa _{i}=1$ and $\kappa _{i}\in [0,1]$, $i\in \{1,2,3\}$. $\kappa _{1}$, $\kappa _{2}$ and $\kappa _{3}$ represent the relative cost weights for vaccination, surveillance, and treatment interventions, respectively. $a_{1}$ and $a_{2}$ are non-negative scaling factors that balance the magnitude between $\phi (e)$ and $\Phi (t,x,{\mathbf {u}})$ in optimal control problem (6), ensuring effective achievement of the terminal control target $\mathbb {I}$.

##### Theorem 4

*There exists an optimal pair* $({\mathbf {u}}^{*},x^*)$ *such that*

$$\begin{aligned} J({\mathbf {u}}^{*})=\min _{{\mathbf {u}}\in U} J({\mathbf {u}}) \end{aligned}$$

*subject to system (S8.1)–(S8.5), where*

$$\begin{aligned} {\mathbf {u}}^{*}=[{\phi _f^{*},k_f^{*},\sigma _{f1}^{u*},\sigma _{f1}^{v*},\sigma _{f2}^{u*}, \sigma _{f2}^{v*},\phi _m^{*},k_m^{*}}] \end{aligned}$$

*is an optimal control, and*

$$\begin{aligned} x^*=(&S^{u*}_{f1}, I^{u*}_{f1}, L^{u*}_{f1}, H^{u*}_{f1}, C^{u*}_{f1}, S^{v*}_{f1}, I^{v*}_{f1}, L^{v*}_{f1}, H^{v*}_{f1}, C^{v*}_{f1},D_{f1}^{*}, R_{f1}^{*}, S^{u*}_{f2}, I^{u*}_{f2}, L^{u*}_{f2}, H^{u*}_{f2}, C^{u*}_{f2}, S^{v*}_{f2}, I^{v*}_{f2},\\&L^{v*}_{f2}, H^{v*}_{f2}, C^{v*}_{f2}, D_{f2}^{*}, R_{f2}^{*},S^{u*}_{m1}, I^{u*}_{m1}, S^{v*}_{m1}, I^{v*}_{m1}, R_{m1}^{*}, S^{u*}_{m2}, I^{u*}_{m2}, S^{v*}_{m2}, I^{v*}_{m2}, R_{m2}^{*}). \end{aligned}$$

*is the state solution corresponding to the optimal control* ${\mathbf {u}}^{*}$.

The proof of Theorem 4 is given in Appendix 9.

Next, we derive necessary conditions of optimal control problem (6) according to Proposition 3 in^22^. The Hamiltonian of optimal control problem (6) is defined as

$$\begin{aligned} \mathcal {H}(t,{\mathbf {u}},x,p)=\Phi (t,x,{\mathbf {u}})+\sum _{i=1}^{34}p_if_i, \end{aligned}$$

where $p(t)=(p_1(t),p_2(t),\cdots ,p_{34}(t))$ and $p_i$ ($i=1,2,\ldots ,34$) are adjoint variables. If $({\mathbf {u}}^{*},x^*)$ is the solution of optimal control problem (6), then there exists *p*(*t*) satisfying the adjoint equations (S10.1-S10.35) (see Appendix 10) such that

$$\begin{aligned} \mathcal {H}(t,{\mathbf {u}}^{*},x^{*},p)=\min _{{\mathbf {u}}\in U}\mathcal {H}(t,{\mathbf {u}},x^{*},p). \end{aligned}$$

Let $u_i$ be the ith element in ${\mathbf {u}}$. By the optimality conditions

$$\begin{aligned} \left. \frac{\partial \mathcal {H}}{\partial u_i}\right| _{(\tilde{\phi }_f,\tilde{k}_f,\tilde{\sigma }^u_{f1},\tilde{\sigma }^v_{f1}, \tilde{\sigma }^u_{f2},\tilde{\sigma }^v_{f2},\tilde{\phi }_m,\tilde{k}_m)}=0,\ i=1,2,\cdots ,8, \end{aligned}$$

we get

$$\begin{aligned}&\tilde{\phi }_f(t,x,p)=\frac{p_{1}-p_{6}}{a_2\kappa _{1}B_1^2A_{f}},\quad \tilde{k}_f(t,x,p)=\frac{p_{1}-p_{6}}{a_2\kappa _{1}B_2^2S^{u}_{f1}},\quad \tilde{\sigma }^u_{f1}(t,x,p)=\frac{\hat{Z}_1}{\hat{M}_1},\quad \tilde{\sigma }^v_{f1}(t,x,p)=\frac{\hat{Z}_2}{\hat{M}_2},\\&\tilde{\sigma }^u_{f2}(t,x,p)=\frac{\hat{Z}_3}{\hat{M}_3},\quad \tilde{\sigma }^v_{f2}(t,x,p)=\frac{\hat{Z}_4}{\hat{M}_4},\quad \tilde{\phi }_m(t,x,p)=\frac{p_{25}-p_{27}}{a_2\kappa _{1}B_1^2A_{m}},\quad \tilde{k}_m(t,x,p)=\frac{p_{25}-p_{27}}{a_2\kappa _{1}B_2^2S^{u}_{m1}}, \end{aligned}$$

where

$$\begin{aligned} \hat{Z}_1&=p_{3}L^u_{f1}+p_{4}H^{u}_{f1}+p_{5}(1-\Lambda )C^{u}_{f1} -p_{11}[L^u_{f1}+H^{u}_{f1}+(1-\Lambda )C^{u}_{f1}]\\&\quad -a_2\kappa _{2}\left( E_1C^{u}_{f1}\right) ^2(1-\Lambda )\Lambda -a_2\kappa _{3}\left( T_{C}C^{u}_{f1}\right) ^2(1-\Lambda )\Lambda ,\\ \hat{M}_1&=a_2\left\{ \kappa _{2}\left( E_1L^u_{f1}\right) ^2+\kappa _{2} \left( E_1H^{u}_{f1}\right) ^2+\kappa _{2}\left( E_1C^{u}_{f1}(t)\right) ^2(1-\Lambda )^2 +\kappa _{3}\left( T_{L}L^u_{f1}\right) ^2\right. \\&\quad \left. +\kappa _{3}\left( T_{H}H^{u}_{f1}\right) ^2 +\kappa _{3}\left( T_{C}C^{u}_{f1}\right) ^2(1-\Lambda )^2 +\kappa _{2}\left[ E_2\left( S^u_{f1}+I^{u}_{f1}+R_{f1}\right) \right] ^2\right\} ,\\ \hat{Z}_2&=p_{8}L^v_{f1}+p_{9}H^{v}_{f1}+p_{10}(1-\Lambda )C^{v}_{f1} -p_{11}[L^v_{f1}+H^{v}_{f1}+(1-\Lambda )C^{v}_{f1}]\\&\quad -a_2\kappa _{2}(E_1C^{v}_{f1})^2(1-\Lambda )\Lambda -a_2\kappa _{3} (T_{C}C^{v}_{f1})^2(1-\Lambda )\Lambda ,\\ \hat{M}_2&=a_2\left\{ \kappa _{2}\left( E_1L^v_{f1}\right) ^2+\kappa _{2} \left( E_1H^{v}_{f1}\right) ^2+\kappa _{2}\left( E_1C^{v}_{f1}\right) ^2(1-\Lambda )^2 +\kappa _{2}\left[ E_2\left( S^v_{f1}+I^{v}_{f1}\right) \right] ^2\right. \\&\quad +\left. \kappa _{3}\left( T_{L}L^v_{f1}\right) ^2+\kappa _{3} \left( T_{H}H^{v}_{f1}\right) ^2+\kappa _{3}(T_{C}C^{v}_{f1})^2(1-\Lambda )^2\right\} ,\\ \hat{Z}_3&=p_{15}L^u_{f2}+p_{16}H^{u}_{f2}+p_{17}(1-\Lambda )C^{u}_{f2} -p_{23}(L^u_{f2}+H^{u}_{f2}+(1-\Lambda )C^{u}_{f2})\\&\quad -a_2\kappa _{2}\left( E_1C^{u}_{f2}\right) ^2(1-\Lambda )\Lambda -a_2\kappa _{3} \left( T_{C}C^{u}_{f2}\right) ^2(1-\Lambda )\Lambda ,\\ \hat{M}_3&=a_2\left\{ \kappa _{2}\left( E_1L^u_{f2}\right) ^2+\kappa _{2}\left( E_1H^{u}_{f2}\right) ^2 +\kappa _{2}\left( E_1C^{u}_{f2}\right) ^2(1-\Lambda )^2+\kappa _{3}\left( T_{L}L^u_{f2}\right) ^2\right. \\&\quad \left. +\kappa _{3}\left( T_{H}H^{u}_{f2}\right) ^2 +\kappa _{3}\left( T_{C}C^{u}_{f2}\right) ^2(1-\Lambda )^2 +\kappa _{2}\left[ E_2\left( S^u_{f2}+I^{u}_{f2}+R_{f2}\right) \right] ^2\right\} ,\\ \hat{Z}_4&=p_{20}L^v_{f2}+p_{21}H^{v}_{f2}+p_{22}(1-\Lambda )C^{v}_{f2} -p_{23}(L^v_{f2}+H^{v}_{f2}+(1-\Lambda )C^{v}_{f2})\\&\quad -a_2\kappa _{2}(E_1C^{v}_{f2})^2(1-\Lambda )\Lambda -a_2\kappa _{3}(T_{C}C^{v}_{f2})^2(1-\Lambda )\Lambda ,\\ \hat{M}_4&=a_2\left\{ \kappa _{2}\left( E_1L^v_{f2}\right) ^2+\kappa _{2}\left( E_1H^{v}_{f2}\right) ^2 +\kappa _{2}\left( E_1C^{v}_{f2}\right) ^2(1-\Lambda )^2+\kappa _{2}\left[ E_2\left( S^v_{f2}+I^{v}_{f2}\right) \right] ^2\right. \\&\quad \left. +\kappa _{3}\left( T_{L}L^v_{f2}\right) ^2+\kappa _{3}\left( T_{H}H^{v}_{f2}\right) ^2 +\kappa _{3}\left( T_{C}C^{v}_{f2}\right) ^2(1-\Lambda )^2\right\} . \end{aligned}$$

Since ${\mathbf {u}}^{*}\in U$, by Remark 4 in^22^, the optimal control of (6) is characterized by

$$\begin{aligned} \phi _f^{*}(t)=&\min \left\{ 0.95,\max \left\{ 0, \tilde{\phi }_f(t,x^*,p)\right\} \right\} ,&\quad k_f^{*}(t)=&\min \left\{ 0.96,\max \left\{ 0, \tilde{k}_f(t,x^*,p)\right\} \right\} ,\\ \sigma ^{u*}_{f1}(t)=&\min \left\{ 0.232,\max \left\{ 0, \tilde{\sigma }^u_{f1}(t,x^*,p)\right\} \right\} ,&\quad \sigma ^{v*}_{f1}(t)=&\min \left\{ 0.232,\max \left\{ 0, \tilde{\sigma }^v_{f1}(t,x^*,p)\right\} \right\} ,\\ \sigma ^{u*}_{f2}(t)=&\min \left\{ 1/3,\max \left\{ 0, \tilde{\sigma }^u_{f2}(t,x^*,p)\right\} \right\} ,&\quad \sigma ^{v*}_{f2}(t)=&\min \left\{ 1/3,\max \left\{ 0, \tilde{\sigma }^v_{f2}(t,x^*,p)\right\} \right\} ,\\ \phi _m^{*}(t)=&\min \left\{ 0.95,\max \left\{ 0, \tilde{\phi }_m(t,x^*,p)\right\} \right\} ,&\quad k_m^{*}(t)=&\min \left\{ 0.96,\max \left\{ 0, \tilde{k}_m(t,x^*,p)\right\} \right\} . \end{aligned}$$

The following indicators are used to measure the advantages and disadvantages of control strategies:

(1) Number of undiagnosed CC patients at time *t*:

$$\begin{aligned} C(t)=C^{u}_{f1}(t)+C^{v}_{f1}(t)+C^{u}_{f2}(t)+C^{v}_{f2}(t). \end{aligned}$$

(2) The total cost of vaccination, screening and treatment is defined as

$$\begin{aligned} Cost_{total}=Cost_{va}+Cost_{sc}+Cost_{tr}. \end{aligned}$$

(3) Within the time interval [0, *T*], the numbers of newly added infected persons ($I_{total}$), CC cases ($C_{total}$) and HSIL individuals ($H_{total}$) are shown as follows:

$$\begin{aligned} I_{total}&=\int _0^T\left[ \sum _{i\in \{1,2\}}\left( \lambda _{mi}S^u_{fi} +(1-\varepsilon _{fi})\lambda _{mi}S^v_{fi}+\lambda _{fi}S^u_{mi} +(1-\varepsilon _{mi})\lambda _{fi}S^v_{mi}\right) \right] \textrm{d}t,\\ H_{total}&=\int _0^T\left[ \sum _{i\in \{1,2\}}\left( (1-e^{u}_{fi})\eta L^{u}_{fi}+(1-e^{v}_{fi})\eta L^{v}_{fi}\right) \right] \textrm{d}t,\\ C_{total}&=\int _0^T\left( \xi ^{u}_{f1}H^{u}_{f1}+\xi ^{v}_{f1}H^{v}_{f1} +\xi ^{u}_{f2}H^{u}_{f2}+\xi ^{v}_{f2}H^{v}_{f2}\right) \textrm{d}t. \end{aligned}$$

(3.1) Further, Infection Averted Rate (IAR)^50^ is defined as

$$\begin{aligned} IAR=\frac{I_{total,c}-I_{total,c_*}}{I_{total,c}}, \end{aligned}$$

where $I_{total,c_*}$ and $I_{total,c}$ represent the numbers of newly infected individuals under and without control measures over the time period [0, *T*], respectively.

(3.2) Cervical Cancer Averted Rate (CCAR) is defined as

$$\begin{aligned} CCAR=\frac{C_{total,c}-C_{total,c_*}}{C_{total,c}}, \end{aligned}$$

where $C_{total,c_*}$ and $C_{total,c}$ represent the numbers of newly added CC cases under and without control strategies over the time period [0, *T*], respectively.

(3.3) HSIL Averted Rate (HCAR) is defined as

$$\begin{aligned} HCAR=\frac{H_{total,c}-H_{total,c_*}}{H_{total,c}}, \end{aligned}$$

where $H_{total,c_*}$ and $H_{total,c}$ denote the numbers of newly added HSIL individuals with and without control strategies over the time period [0, *T*], respectively.

(4) Disability-Adjusted Life Years (DALY)^51,52^ refer to years of healthy life lost due to LSIL, HSIL and CC, and is composed of Years of Life Lost (YLL) and Years Lost due to Disability (YLD). Then,

$$\begin{aligned} DALY&=YLL+YLD\\&=\int _0^T(a_{f1}*d_{f1}*D_{f1}(t)+a_{f2}*d_{f2}*D_{f2}(t))\textrm{d}t\\&\quad +\int _0^T\sum _{g\in \{u,v\}}\left\{ \frac{(1-r^{g}_{f1})\rho I^{g}_{f1}DW_{L}}{\eta +\sigma ^{g}_{f1}+\mu _{f1}+\alpha _{f}} +\frac{(1-e^{g}_{f1})\eta L^{g}_{f1}DW_{H}}{\xi ^{g}_{f1}+\sigma ^{g}_{f1}+\mu _{f1}+\alpha _{f}} +\frac{\xi ^{g}_{f1}H^{g}_{f1}DW_{C}}{\Lambda +(1-\Lambda )\sigma ^{g}_{f1}+\mu _{f1}+\alpha _{f}} +\frac{[\Lambda +(1-\Lambda )\sigma ^{g}_{f1}]C^{g}_{f1}DW_{CD}}{\gamma _{f1}+d_{f1}+\mu _{f1}+\alpha _{f}} \right. \\&\quad \left. +\frac{\sigma ^{g}_{f1}L^{g}_{f1}DW_{LD}+\sigma ^{g}_{f1}H^{g}_{f1}DW_{HD}}{\gamma _{f1}+d_{f1}+\mu _{f1}+\alpha _{f}} \right\} \textrm{d}t +\int _0^T\sum _{g\in \{u,v\}}\left( \frac{[\alpha _{f}L^{g}_{f1}+(1-r^{g}_{f2})\rho I^{g}_{f2}]DW_{L}}{\eta +\sigma ^{g}_{f2}+\mu _{f2}} +\frac{[\alpha _{f}H^{g}_{f1}+(1-e^{g}_{f2})\eta L^{g}_{f2}]DW_{H}}{\xi ^{g}_{f2}+\sigma ^{g}_{f2}+\mu _{f2}} \right. \\&\quad \left. +\frac{[\alpha _{f}C^{g}_{f1}+\xi ^{g}_{f2}H^{g}_{f2}]DW_{C}}{\Lambda +(1-\Lambda )\sigma ^{g}_{f2}+\mu _{f2}} .+\frac{\sigma ^{g}_{f2}L^{g}_{f2}DW_{LD}}{\gamma _{f2}+d_{f2}+\mu _{f2}} +\frac{[\Lambda +(1-\Lambda )\sigma ^{g}_{f2}]C^{g}_{f2}DW_{CD}+\sigma ^{g}_{f2}H^{g}_{f2}DW_{HD}}{\gamma _{f2}+d_{f2}+\mu _{f2}} \right) \textrm{d}t +\int _0^T\frac{\alpha _{f}D_{f1}DW_{D}}{\gamma _{f2}+d_{f2}+\mu _{f2}} \textrm{d}t, \end{aligned}$$

where $a_{f1}$ and $a_{f2}$ represent the average remaining life expectancy at the age of death for CC patients in the first and second age groups, respectively; $DW_{L}$, $DW_{H}$ and $DW_{C}$ represent the pre-treatment disability weights (DW) for LSIL, HSIL and CC, respectively; $DW_{LD}$, $DW_{HD}$ and $DW_{CD}$ represent the post-treatment disability weights for LSIL, HSIL and CC, respectively; $DW_{D}$ represents the average post-treatment disability weights of the individual after transferring from $D_{f1}$ to $D_{f2}$ due to the increase of age. Since individuals in compartment $D_{f1}$ originate from compartments $L^{u}_{f1}$, $H^{u}_{f1}$, $C^{u}_{f1}$, $L^{v}_{f1}$, $H^{v}_{f1}$ and $C^{v}_{f1}$, we define

$$\begin{aligned} DW_{D}=\frac{\sum _{g\in \{u,v\}}L^{g}_{f1}DW_{LD}+\sum _{g\in \{u,v\}}H^{g}_{f1}DW_{HD} +\sum _{g\in \{u,v\}}C^{g}_{f1}DW_{CD}}{\sum _{g\in \{u,v\}}(L^{g}_{f1}+H^{g}_{f1}+C^{g}_{f1})}. \end{aligned}$$

In addition, $1/(\eta +\sigma ^{g}_{f1}+\mu _{f1}+\alpha _{f})$, $1/(\xi ^{g}_{f1}+\sigma ^{g}_{f1}+\mu _{f1}+\alpha _{f})$, $1/(\Lambda +(1-\Lambda )\sigma ^{g}_{f1}+\mu _{f1}+\alpha _{f})$ and $1/(\gamma _{f1}+d_{f1}+\mu _{f1}+\alpha _{f})$ represent the average duration in compartments $L^{g}_{f1}$, $H^{g}_{f1}$, $C^{g}_{f1}$ and $D_{f1}$, respectively; $1/(\eta +\sigma ^{g}_{f2}+\mu _{f2})$, $1/(\xi ^{g}_{f2}+\sigma ^{g}_{f2}+\mu _{f2})$, $1/(\Lambda +(1-\Lambda )\sigma ^{g}_{f2}+\mu _{f2})$ and $1/(\gamma _{f2}+d_{f2}+\mu _{f2})$ represent the average duration in compartments $L^{g}_{f2}$, $H^{g}_{f2}$, $C^{g}_{f2}$ and $D_{f2}$, respectively; $(1-r^{g}_{fi})\rho I^{g}_{fi}$, $(1-e^{g}_{fi})\eta L^{g}_{fi}$ and $\xi ^{g}_{fi}H^{g}_{fi}$ represent the number of individuals increasing at time *t* in $L^{g}_{fi}$, $H^{g}_{fi}$, and $C^{g}_{fi}$ ($i\in \{1,2\}$), respectively; $\sigma ^{g}_{fi}L^{g}_{fi}$, $\sigma ^{g}_{fi}H^{g}_{fi}$, and $[\Lambda +(1-\Lambda )\sigma ^{g}_{fi}]C^{g}_{fi}$ represent individuals transferred from $L^{g}_{fi}$, $H^{g}_{fi}$, and $C^{g}_{fi}$ to $D_{fi}$ at time *t*, respectively; $\alpha _{f}L^{g}_{f1}$, $\alpha _{f}H^{g}_{f1}$, $\alpha _{f}C^{g}_{f1}$ and $\alpha _{f}D_{f1}$ represent the number of individuals at time *t* who move from the first age group to the second age group due to increasing age.

The initial value of the variable whose superscript is not *v* is the value corresponding to each variable in 2017, and the initial value of the variable whose superscript is *v* is equal to 0. Assume that $d_{f1}=0.01$ and $d_{f2}=0.0226$. The values of other parameters can be seen in Tables 3 and 4. In addition, when $r^v_{fi}=1$, $e^v_{fi}=1$ and $\xi ^v_{fi}=0$ ($i\in \{1,2\}$), $L^v_{fi}(t)$, $H^v_{fi}(t)$ and $C^v_{fi}(t)$ are equal to 0. Hence $\sigma ^v_{f1}$ and $\sigma ^v_{f2}$ are equal to 0. The target value $\mathbb {I}$ is equal to 0. Based on the content of Appendix 11, we choose a value greater than 50 as the value of *T*. The values of the parameters involved in cost and DALY are shown in Table S4 (see Appendix 12). In the following, no control means not vaccinating but screening, that is, $\phi _f(t)=0$, $k_f(t)=0$, $\sigma ^u_{f1}(t)=0.1916$, $\sigma ^v_{f1}(t)=0$, $\sigma ^u_{f2}(t)=0.1916$, $\sigma ^v_{f2}(t)=0$, $\phi _m(t)=0$ and $k_m(t)=0$, where 0.1916 refers to mean value of the screening rate in 2018–2020. We mainly compare the following four control strategies:

S1: Only 14-year-old females are vaccinated.

S2: Only 14-year-old females and males are vaccinated.

S3: Females aged 14 and in the first age group are vaccinated.

S4: Females and males aged 14 and in the first age group are vaccinated.

First, we made the following selection for the weight parameters in (6): $a_1=1$, $a_2=10^{-10}$ and $\kappa _1=\kappa _2=\kappa _3=1/3$. When $T=53$, the numerical results of the optimal control problem (6) are shown in Fig. 11. (1) The optimal control corresponding to S3 is as follows (see Fig. 11c). When $t<48.5$, $\phi ^*_f$ is the maximum value. When $t>48.5$, $\phi ^*_f$ gradually decreases to 0. When $t<1.65$, $k^*_f$ gradually increases from 0.5047 to 0.96. When $1.65<t<50.7$, $k^*_f$ is always equal to 0.96. When $t>50.7$, $k^*_f$ gradually decreases to 0. (2) The optimal control corresponding to S4 is as follows (see Fig. 11d). $\phi ^*_f$ and $\phi ^*_m$ first maintain the maximum value, then decrease at a slower rate, and finally decrease at a faster rate. $k^*_f$ first increases to the maximum value in a short period of time, and then decreases rapidly with $\phi ^*_f$. When $t<18$, $k^*_m$ increases from 0.13 to 0.96. When $\phi ^*_m$ decreases slowly, $k^*_m$ is always the maximum value. Finally, $\phi ^*_m$ and $k^*_m$ decrease rapidly together. $\phi ^*_f\ge \phi ^*_m$ and $k^*_f\ge k^*_m$. (3) The optimal screening rates corresponding to the four strategies are not always the maximum. (4) It can be seen from Fig. 11f and g that if $T=53$,

$$\begin{aligned}&IAR=0.47,~HCAR=0.43,~CCAR=0.42,~Cost_{total}=2.013\times 10^{11},~\text {for S1};\\&IAR=0.60,~HCAR=0.51,~CCAR=0.48,~Cost_{total}=1.828\times 10^{11},~\text {for S2};\\&IAR=0.78,~HCAR=0.69,~CCAR=0.64,~Cost_{total}=1.213\times 10^{11},~\text {for S3};\\&IAR=0.83,~HCAR=0.73,~CCAR=0.66,~Cost_{total}=1.175\times 10^{11},~\text {for S4}. \end{aligned}$$

*C*(*t*), $C_T$, $Cost_{total}$ and *DALY* of S4 are the smallest, and *IAR*, *HCAR* and *CCAR* of S4 are the largest (see Fig. 11e–h).

For the same weight parameter value, when $T=73$, the numerical results of the optimal control problem (6) are shown in Fig. 12. The optimal control after increasing the control time *T* has the following similarities and differences: (1) As shown in Figs. 11a–c, 12a–c, under the two control times, the increase and decrease trends of the optimal control corresponding to S1, S2 and S3 are basically the same. Figures 11b and 12b demonstrate that for S2, the high vaccination rate for 14-year-old girls remains sustained for a longer duration compared with that for 14-year-old boys. Figures 11c, d, 12c and d show that for S3 and S4, we need to focus on catch-up vaccination rates for females in the first age group in the late stages of control. (2) Figures 11d and 12d demonstrate that the trends of curves $\phi ^*_f$ $\phi ^*_m$ and $k^*_m$ diverge when *T* varies. For S4, $\phi ^*_f$ starts to decline earlier when $T=73$ compared to when $T=53$. When $T=73$, the trend of parameter $\phi ^*_m$ corresponding to S4 consists of four segments, first maintains the maximum value, then decreases at a faster rate, then decreases at a slower rate, and finally decreases at a faster rate. $k^*_m$ corresponding to S4 is always the maximum value when $\phi ^*_m$ slowly decreases, and the time to maintain the maximum value is shorter than that when $T=53$. (3) $\sigma _{f1}^{u*}$ and $\sigma _{f2}^{u*}$ when $T=73$ is less than or equal to that when $T=53$. (4) It can be seen from Fig. 12f and g that if $T=73$,

$$\begin{aligned}&IAR=0.51,~HCAR=0.47,~CCAR=0.44,~Cost_{total}=2.258\times 10^{11},~\text {for S1};\\&IAR=0.67,~HCAR=0.58,~CCAR=0.52,~Cost_{total}=1.943\times 10^{11},~\text {for S2};\\&IAR=0.81,~HCAR=0.74,~CCAR=0.68,~Cost_{total}=1.261\times 10^{11},~\text {for S3};\\&IAR=0.86,~HCAR=0.78,~CCAR=0.71,~Cost_{total}=1.183\times 10^{11},~\text {for S4}. \end{aligned}$$

When $T=73$, $C_T$ is closer to 0; *IAR*, *HCAR* and *CCAR* are larger. Therefore, the longer control time *T*, the better disease control effect of the corresponding optimal control ${\mathbf {u}}^{*}$. (5) As is the case with $T=53$, when $T=73$, the optimal control about S4 achieves the best control effect with the minimum cost.

Next, we investigate the impact of the weight parameter values on the optimal control corresponding to S3 and S4. Firstly, we investigate the impact of the weight parameter $a_2$ on the optimal control. The numerical results of the optimal control under different values of weight parameter $a_2$ are shown in Figs. 13 and 14. For S3 and S4, the smaller the value of $a_2$, the larger the value of all control variables, and the later the time when variables $\phi ^*_f$, $\phi ^*_m$, $k^*_f$ and $k^*_m$ start to decrease. When $a_2=10^{-5}$, $a_2=10^{-10}$ and $t>30$, *C*(*t*) corresponding to S3 is larger than *C*(*t*) corresponding to S4. However, when $a_2=10^{-15}$ and $t>30$, *C*(*t*) corresponding to S3 is smaller than *C*(*t*) corresponding to S4. The smaller $a_2$, the closer $C_T$ is to 0, the smaller *DALY*, and the larger $Cost_{total}$, *IAR*, *HCAR* and *CCAR*. In addition, for indicators $C_T$, $Cost_{total}$, *IAR*, *HCAR* and *CCAR*, the change rate of S3 is greater than that of S4. In short, reducing the weight parameter $a_2$ will make the control effect better, but at the same time the cost will be higher.

Secondly, we investigate the impact of the weight parameters $\kappa _1$, $\kappa _2$ and $\kappa _3$ on the optimal control. We mainly conduct numerical simulations on the control problems corresponding to the following seven weight values:

$$\begin{aligned}&C1: \kappa _1=1/4,\quad \kappa _2=1/2,\quad \kappa _3=1/4;&\quad&C2: \kappa _1=1/3,\quad \kappa _2=1/3,\quad \kappa _3=1/3; \\&C3: \kappa _1=3/8,\quad \kappa _2=3/8,\quad \kappa _3=1/4;&\quad&C4: \kappa _1=5/12,\quad \kappa _2=1/6,\quad \kappa _3=5/12; \\&C5: \kappa _1=1/2,\quad \kappa _2=1/4,\quad \kappa _3=1/4;&\quad&C6:\kappa _1=3/4, \quad \kappa _2=1/8,\quad \kappa _3=1/8; \\&C7: \kappa _1=9/10,\quad \kappa _2=1/20,\quad \kappa _3=1/20. \end{aligned}$$

According to numerical results of the optimal control under different values of weight parameters $\kappa _1$, $\kappa _2$ and $\kappa _3$ (see Figs. 15, 16 and 17), we get the following conclusions: (1) From C1, C2 and C4, when $\kappa _1=\kappa _3$ and $\kappa _2$ decrease, duration of the maximum value of parameters $\phi ^*_f$, $\phi ^*_m$, $k^*_f$ and $k^*_m$ decreases; but duration of the maximum value of parameters $\sigma ^{u*}_{f1}$ and $\sigma ^{u*}_{f2}$ increases; parameter $k^*_m$ decreases obviously. From the changes of indicators *C*(*t*), $C_{T}$, *IAR*, *HCAR*, *CCAR* and *DALY*, control effect is getting better and better; but $Cost_{total}$ is not always increasing, and $Cost_{total}$ about C2 is the smallest. (2) From C7, C6, C5 and C2, when $\kappa _2=\kappa _3$ and $\kappa _1$ decrease, duration of the maximum value of parameters $\phi ^*_f$, $\phi ^*_m$, $k^*_f$ and $k^*_m$ increases; but duration of the maximum value of parameters $\sigma ^{u*}_{f1}$ and $\sigma ^{u*}_{f2}$ decreases; parameter $k^*_m$ increases obviously. Although $C_T$ increases, it can be seen from the values of *C*(*t*), *IAR*, *HCAR*, *CCAR* and *DALY* that control effect is getting better and better, and $Cost_{total}$ is getting smaller and smaller. (3) From C2 and C3, when $\kappa _1=\kappa _2$ and $\kappa _3$ decrease, $Cost_{total}$ becomes higher, but control effect becomes worse. (4) From C1, C3 and C5, when $\kappa _3=1/4$, $\kappa _1$ increases and $\kappa _2$ decreases, inoculation rates in optimal control decrease, and screening rates increase. *C*(*t*) and $C_{T}$ are smaller, $Cost_{total}$ is larger, and change rates of *IAR*, *HCAR* and *DALY* is very small. (5) From C7, C6, C5, C2 and C4, when sum of $\kappa _1$ and $\kappa _2$ decreases and $\kappa _3$ increases, $Cost_{total}$ of optimal control is lower and lower, and the control effect is better.

To sum up, we can reduce the weight parameter $a_2$ to make $C_T$ smaller and reach target $\mathbb {I}$ earlier. In addition, we can reduce the cost of optimal control by increasing $\kappa _3$ and decreasing $\kappa _1$.

Media coverage is also a control measure. Media coverage can enhance people’s understanding of HPV, thereby raising awareness of prevention and further reducing transmission coefficients $\beta _{mij}$ and $\beta _{fij}$ ($i,j\in \{1,2\}$). Transmission rate decreases from $\beta _{mij}$ and $\beta _{fij}$ to $pro\beta _{mij}$ and $pro\beta _{fij}$ through media coverage, where *pro* is proportion of reduced transmission rate to original transmission rate. For S3 and S4, numerical results of the optimal control problem (6) with different values of parameter *pro* are shown in Fig. 18. As *pro* decreases, intensity of vaccination and screening decreases. And with reduction of *pro*, control effect is better, and cost $Cost_{total}$ is lower. $C_T$, $Cost_{total}$, averted rate and *DALY* all have a linear relationship with *pro*. Among the optimal control corresponding to different *pro*, change of $k^*_m$ is the most obvious.

The above numerical simulations are all carried out on the basis that protective efficacy of the vaccine to LSIL, HSIL and CC is $100\%$. Next, we study the optimal control problem for situations where the protective efficacy is less than $100\%$. According to range of parameter values (see Table 3), the following three situations are selected:

$$\begin{aligned}&C1:\quad \varepsilon _{f1}=\varepsilon _{f2}=\varepsilon _{m1}=\varepsilon _{m2}=0.94, \quad r^v_{f1}=0.99, \quad r^v_{f2}=0.96,\\ &\quad \quad \quad e^v_{f1}=0.96,\quad e^v_{f2}=0.9,\quad \xi ^v_{f1}=0.03,\quad \xi ^v_{f2}=0.045;\\&C2:\quad \varepsilon _{f1}=\varepsilon _{f2}=\varepsilon _{m1}=\varepsilon _{m2}=0.8416, \quad r^v_{f1}=0.975, \quad r^v_{f2}=0.91, \\ &\quad \quad \quad e^v_{f1}=0.91, \quad e^v_{f2}=0.82,\quad \xi ^v_{f1}=0.04,\quad \xi ^v_{f2}=0.055;\\&C3:\quad \varepsilon _{f1}=\varepsilon _{f2}=\varepsilon _{m1}=\varepsilon _{m2}=0.74, \quad r^v_{f1}=0.95,\quad r^v_{f2}=0.86,\\ &\quad \quad \quad e^v_{f1}=0.86,\quad e^v_{f2}=0.74,\quad \xi ^v_{f1}=0.05,\quad \xi ^v_{f2}=0.07. \end{aligned}$$

The corresponding numerical results are shown in Fig. 19. When the protective effect of the vaccine is reduced, screening rates $\sigma ^{u*}_{f1}$, $\sigma ^{v*}_{f1}$, $\sigma ^{u*}_{f2}$, and $\sigma ^{v*}_{f2}$ in optimal control are larger, $C_T$, $Cost_{total}$ and *DALY* are larger, and *IAR*, *HCAR* and *CCAR* are smaller.

## Conclusion and discussion

This study develops a two-sex age-structured transmission model to investigate optimal CC control strategies in China under resource constraints and cost minimization objectives. The model captures HPV transmission dynamics, CC progression pathways and current intervention strategies. Theoretical analysis establishes the basic reproduction number ($\mathcal {R}_{0}$) as the critical epidemic threshold, where values exceeding 1 indicate disease persistence while values below 1 correspond to a locally asymptotically stable disease-free equilibrium. Parameter estimation is performed through model fitting to China’s HPV16/18-associated CC incidence and mortality data. This calibration process generates estimates for unknown parameters and time-varying reproduction numbers ($\mathcal {R}_{t}$). To account for surveillance data uncertainty, we implement multiple fittings under the assumption of negative binomial distributed reporting errors, yielding 95% confidence intervals for all estimated parameters and $\mathcal {R}_{t}$ values. During 2006–2016, the 95% confidence intervals for the reproduction number were as follows: [1.369,  1.602] for HPV16/18, [1.338,  1.556] for HPV16, and [1.178,  1.39] for HPV18.

Previous studies on HPV’s basic reproduction number ($\mathcal {R}_{0}$) in China have reported varying estimates under different methodological approaches. Rifhat et al. derived $\mathcal {R}_{0}=1.1490$ $(95\%CI: 0.6778{-}1.9084)$ through fitting a deterministic HPV model to annual CC cases in Xinjiang (2003–2019)^53^, while their subsequent stochastic modeling of monthly cases from 2003 to 2020 yielded $\mathcal {R}_{0}=1.3496$ $(95\%CI:1.3458{-}1.3716)$^54^. Alternative estimates include Gao et al.’s $\mathcal {R}_{0}=1.0333$ for HPV16/18 from a cohort study in Liuzhou^20^, and Xu et al.’s hospital-based estimates of $\mathcal {R}_{0}=3.2$ $(95\%CI:0.00{-}6.46)$ for HPV18 and $\mathcal {R}_{0}=2.03$ $(95\%CI:0.5{-}2.87)$ for HPV16 from Xiamen data^55^. These discrepancies likely stem from variations in model assumptions, geographical coverage, and HPV-type specificity. Our nationwide analysis, based on fitting model (1)–(4) to comprehensive case data, produces estimates most aligned with Rifhat et al.’s Xinjiang studies. While cohort and single-hospital studies provide valuable localized insights, our population-level approach offers stronger epidemiological representativeness. Given the paucity of $\mathcal {R}_{0}$ estimates for HPV in China, these findings significantly advance understanding of national transmission dynamics and establish a crucial evidence base for developing vaccine allocation strategies.

Through PRCC-based sensitivity analysis, we identified vaccination rate parameters ($k_f$, $k_m$, $\phi _f$, $\phi _m$ ) as key determinants of disease control efficacy. Building upon the reproduction number framework, we formulated the optimization problem (5) to address vaccine allocation challenges during initial market introduction. Numerical simulations revealed an optimal prioritization sequence: (1) 14-year-old girls as highest priority, (2) 14-year-old boys as secondary priority, and (3) remaining vaccines allocated preferentially to females over males aged 15–44. This strategy minimized the basic reproduction number to $\mathcal {R}_{0}\le 0.244$ when covering both 14-year-old girls and boys annually. While previous studies advocated prioritizing adolescent girls exclusively^20,56^, our inclusion of catch-up vaccination for 15–44 year-olds established adolescent boys as the second priority group—a finding aligned with the evidence presented by Elbasha et al. for male inclusion in vaccination programs^16–18^, yet advancing the field through comprehensive prioritization analysis.

We further developed the optimal control problem (6), incorporating epidemiological measures, cost parameters, and control targets. After proving the existence of optimal control and deriving necessary conditions, our numerical simulations demonstrated that including both adolescent boys and adult males minimized both control costs and DALYs compared to female-only strategies. These results highlight the critical importance of male inclusion, particularly 14-year-old boys, in vaccination programs. Our China-specific analysis of HPV16/18 transmission, grounded in actual vaccine and screening costs, provides particularly relevant policy insights for CC control in China.

Our findings reveal distinct patterns in optimal vaccine deployment compared to existing literature. Previous studies have proposed different approaches: Reference^24^ recommended maintaining peak vaccination rates during the initial implementation phase before gradual reduction, while reference^57^ suggested initiating all vaccination rates at maximum levels followed by continuous decline throughout the intervention period. Notably, our optimal vaccination deployment strategy differs from previous work in three key aspects: (1) 14-year-old girls and boys maintain maximum initial vaccination rates before gradual reduction, (2) females aged $\ge 15$ years follow a bell-shaped vaccination curve, and (3) adult male vaccination depends critically on weight parameters in the objective function. These parameters ($a_1$, $a_2$, $\kappa _1$-$\kappa _3$) must be carefully calibrated according to control duration *T*, targets $\mathbb {I}$ and budget constraints. Additional findings emphasize that media-driven awareness campaigns can enhance cost-effectiveness, that vaccinated women still require screening (especially $\ge 45$ years), and that transmission rate reduction remains crucial for HPV control.

While applicable to all high-risk HPV types, our model requires adaptation for low-risk HPV. The current allocation strategy is designed to address vaccine deployment challenges during the initial launch phase; future work will explore time-varying allocation approaches under limited supply. Provincial-level data could further enable spatially optimized control strategies across China’s different regions.

## Supplementary Information

Supplementary Information.

## Data Availability

Data availability All data generated or analysed during this study are included in this article and its supplementary information files.
