# Supplementary material for: HPV transmission and optimal control of cervical cancer in China
Source: Sci Rep. 2025 Jul 1;15:21354. doi: 10.1038/s41598-025-05514-y (PMC12218410; doi:10.1038/s41598-025-05514-y)
Supplement: Supplementary file 1 — Supplementary Information. [file 41598_2025_5514_MOESM1_ESM.pdf]

HPV transmission and optimal control of cervical cancer in China

---

Appendix 1. Proof of Theorem 3.1

Proof. We rewrite system (2.1)-(2.4) in the following form

$$\frac{\mathrm{d}x}{\mathrm{d}t} = F(t,x), \tag{S1.1}$$

where $F(t,x) = (F_1(t,x), \cdots, F_{34}(t,x))$. Obviously, $F(t,x)$ has the first-order continuous partial derivatives with respect to x, thus $F(t,x)$ is locally Lipschitz. In addition, it is easily checked that $F_i(t,x) \geq 0$, when $x \in \mathbb{R}^{34}_+$ and $x_i = 0$. By Theorem A.4 in [1], for any $x(0) \in \mathbb{R}^{34}_+$, system (S1.1) has a unique nonnegative solution defined on some interval $[0,b)$, and if $b < \infty$, then $\limsup_{t\to b_-} N_f(t) = \infty$ and $\limsup_{t\to b_-} N_m(t) = \infty$. By calculation,

$$\begin{aligned}
\frac{dN_f}{dt} &= A_f - \mu_{f1}N_{f1} - \mu_{f2}N_{f2} - d_{f1}D_{f1} - d_{f2}D_{f2} \leq A_f - \min\{\mu_{f1}, \mu_{f2}\}N_f,\\
\frac{dN_m}{dt} &= A_m - \mu_{m1}N_{m1} - \mu_{m2}N_{m2} \leq A_m - \min\{\mu_{m1}, \mu_{m2}\}N_m.
\end{aligned}$$

Thus,

$$\begin{aligned}
N_f(t) &\leq \left(N_f(0) - \frac{A_f}{\min\{\mu_{f1}, \mu_{f2}\}}\right) e^{-\min\{\mu_{f1},\mu_{f2}\}t} + \frac{A_f}{\min\{\mu_{f1}, \mu_{f2}\}},\\
N_m(t) &\leq \left(N_m(0) - \frac{A_m}{\min\{\mu_{m1}, \mu_{m2}\}}\right) e^{-\min\{\mu_{m1},\mu_{m2}\}t} + \frac{A_m}{\min\{\mu_{m1}, \mu_{m2}\}}.
\end{aligned}$$

Obviously, $N_f \leq \max\left\{N_f(0), \frac{A_f}{\min\{\mu_{f1},\, \mu_{f2}\}}\right\}$ and $N_m \leq \max\left\{N_m(0), \frac{A_m}{\min\{\mu_{m1},\, \mu_{m2}\}}\right\}$. This result contradicts $\limsup_{t\to b_-} N_f(t) = \infty$. Hence, $b = \infty$. Then, by the standard comparison theorem, we have $\limsup_{t\to+\infty} N_f(t) \leq \frac{A_f}{\min\{\mu_{f1},\, \mu_{f2}\}}$ and $\limsup_{t\to+\infty} N_m(t) \leq \frac{A_m}{\min\{\mu_{m1},\, \mu_{m2}\}}$. In particular, when $N_f(0) \leq \frac{A_f}{\min\{\mu_{f1},\, \mu_{f2}\}}$ and $N_m(0) \leq \frac{A_m}{\min\{\mu_{m1},\, \mu_{m2}\}}$, $N_f(t) \leq \frac{A_f}{\min\{\mu_{f1},\, \mu_{f2}\}}$ and $N_m(t) \leq \frac{A_m}{\min\{\mu_{m1},\, \mu_{m2}\}}$. Hence, the region $\mathbb{D}$ is the positively invariant set for system (2.1)-(2.4).

Appendix 2. Proof of Theorem 3.2

Proof. The Jacobian matrix of system (2.1)-(2.4) at $\bar{E}$ is

$$J|_{\bar{E}} = \begin{bmatrix} F - V & \boldsymbol{O} \\ J_1 & J_2 \end{bmatrix},$$

where

$$J_1 = \begin{bmatrix} J_{11} & J_{12} \\ J_{13} & J_{14} \end{bmatrix},$$

in which

$$J_{11} = \begin{bmatrix} 0 & 0 & 0 & 0 & 0 & 0 & 0 & 0 & 0 & 0 & 0 & 0 & 0 & 0 & 0 & 0 \\ 0 & 0 & 0 & 0 & 0 & 0 & 0 & 0 & 0 & 0 & 0 & 0 & 0 & 0 & 0 & 0 \\ 0 & \sigma_{f1}^u & \sigma_{f1}^u & J_{11,1} & 0 & \sigma_{f1}^v & \sigma_{f1}^v & J_{11,2} & 0 & 0 & 0 & 0 & 0 & 0 & 0 & 0 \\ r_{f1}^u\rho & e_{f1}^u\eta & 0 & 0 & r_{f1}^v\rho & e_{f1}^v\eta & 0 & 0 & 0 & 0 & 0 & 0 & 0 & 0 & 0 & 0 \\ 0 & 0 & 0 & 0 & 0 & 0 & 0 & 0 & 0 & 0 & 0 & 0 & 0 & 0 & 0 & 0 \\ 0 & 0 & 0 & 0 & 0 & 0 & 0 & 0 & 0 & 0 & 0 & 0 & 0 & 0 & 0 & 0 \\ 0 & 0 & 0 & 0 & 0 & 0 & 0 & 0 & 0 & \sigma_{f2}^u & \sigma_{f2}^u & J_{11,3} & 0 & \sigma_{f2}^v & \sigma_{f2}^v & J_{11,4} \\ 0 & 0 & 0 & 0 & 0 & 0 & 0 & 0 & r_{f2}^u\rho & e_{f2}^u\eta & 0 & 0 & r_{f2}^v\rho & e_{f2}^v\eta & 0 & 0 \end{bmatrix},$$

$$J_{13} = \begin{bmatrix} -f_{51} & -f_{51} & -f_{51} & -f_{51} & -f_{51} & -f_{51} & -f_{51} & -f_{51} & -f_{61} & -f_{61} & -f_{61} & -f_{61} & -f_{61} & -f_{61} & -f_{61} & -f_{61} \\ -f_{52} & -f_{52} & -f_{52} & -f_{52} & -f_{52} & -f_{52} & -f_{52} & -f_{52} & -f_{62} & -f_{62} & -f_{62} & -f_{62} & -f_{62} & -f_{62} & -f_{62} & -f_{62} \\ 0 & 0 & 0 & 0 & 0 & 0 & 0 & 0 & 0 & 0 & 0 & 0 & 0 & 0 & 0 & 0 \\ -f_{71} & -f_{71} & -f_{71} & -f_{71} & -f_{71} & -f_{71} & -f_{71} & -f_{71} & -f_{81} & -f_{81} & -f_{81} & -f_{81} & -f_{81} & -f_{81} & -f_{81} & -f_{81} \\ -f_{72} & -f_{72} & -f_{72} & -f_{72} & -f_{72} & -f_{72} & -f_{72} & -f_{72} & -f_{82} & -f_{82} & -f_{82} & -f_{82} & -f_{82} & -f_{82} & -f_{82} & -f_{82} \\ 0 & 0 & 0 & 0 & 0 & 0 & 0 & 0 & 0 & 0 & 0 & 0 & 0 & 0 & 0 & 0 \end{bmatrix},$$

$$J_{12} = \begin{bmatrix} -f_{11} & -f_{11} & -f_{21} & -f_{21} \\ -f_{12} & -f_{12} & -f_{22} & -f_{22} \\ 0 & 0 & 0 & 0 \\ 0 & 0 & 0 & 0 \\ -f_{31} & -f_{31} & -f_{41} & -f_{41} \\ -f_{32} & -f_{32} & -f_{42} & -f_{42} \\ 0 & 0 & 0 & 0 \\ 0 & 0 & 0 & 0 \end{bmatrix}, \; J_{14} = \begin{bmatrix} 0 & 0 & 0 & 0 \\ 0 & 0 & 0 & 0 \\ \omega_{m1}^u & \omega_{m1}^v & 0 & 0 \\ 0 & 0 & 0 & 0 \\ 0 & 0 & 0 & 0 \\ 0 & 0 & \omega_{m2}^u & \omega_{m2}^v \end{bmatrix},$$

$$J_2=\begin{bmatrix}
J_{2,1} & \delta & 0 & \zeta_{f1} & 0 & 0 & 0 & 0 & 0 & 0 & 0 & 0 & 0 & 0\\
k_f & J_{2,2} & 0 & 0 & 0 & 0 & 0 & 0 & 0 & 0 & 0 & 0 & 0 & 0\\
0 & 0 & J_{2,3} & 0 & 0 & 0 & 0 & 0 & 0 & 0 & 0 & 0 & 0 & 0\\
0 & 0 & \gamma_{f1} & J_{2,4} & 0 & 0 & 0 & 0 & 0 & 0 & 0 & 0 & 0 & 0\\
\alpha_f & 0 & 0 & 0 & J_{2,5} & \delta & 0 & \zeta_{f2} & 0 & 0 & 0 & 0 & 0 & 0\\
0 & \alpha_f & 0 & 0 & 0 & J_{2,6} & 0 & 0 & 0 & 0 & 0 & 0 & 0 & 0\\
0 & 0 & \alpha_f & 0 & 0 & 0 & J_{2,7} & 0 & 0 & 0 & 0 & 0 & 0 & 0\\
0 & 0 & 0 & \alpha_f & 0 & 0 & \gamma_{f2} & J_{2,8} & 0 & 0 & 0 & 0 & 0 & 0\\
0 & 0 & 0 & 0 & 0 & 0 & 0 & 0 & J_{2,9} & \delta & \zeta_{m1} & 0 & 0 & 0\\
0 & 0 & 0 & 0 & 0 & 0 & 0 & 0 & k_m & J_{2,10} & 0 & 0 & 0 & 0\\
0 & 0 & 0 & 0 & 0 & 0 & 0 & 0 & 0 & 0 & J_{2,11} & 0 & 0 & 0\\
0 & 0 & 0 & 0 & 0 & 0 & 0 & 0 & \alpha_m & 0 & 0 & J_{2,12} & \delta & \zeta_{m2}\\
0 & 0 & 0 & 0 & 0 & 0 & 0 & 0 & 0 & \alpha_m & 0 & 0 & J_{2,13} & 0\\
0 & 0 & 0 & 0 & 0 & 0 & 0 & 0 & 0 & 0 & \alpha_m & 0 & 0 & J_{2,14}
\end{bmatrix},$$

with

$$\begin{aligned}
&J_{11,1}=\Lambda+(1-\Lambda)\sigma^u_{f1}, && J_{11,2}=\Lambda+(1-\Lambda)\sigma^v_{f1}, && J_{11,3}=\Lambda+(1-\Lambda)\sigma^u_{f2},\\
&J_{11,4}=\Lambda+(1-\Lambda)\sigma^v_{f2}, && J_{2,1}=-(k_f+\mu_{f1}+\alpha_f), && J_{2,2}=-(\delta+\mu_{f1}+\alpha_f),\\
&J_{2,3}=-(\gamma_{f1}+d_{f1}+\mu_{f1}+\alpha_f), && J_{2,4}=-(\mu_{f1}+\alpha_f+\zeta_{f1}), && J_{2,5}=-\mu_{f2},\\
&J_{2,6}=-(\delta+\mu_{f2}), && J_{2,7}=-(\gamma_{f2}+d_{f2}+\mu_{f2}), && J_{2,8}=-(\mu_{f2}+\zeta_{f2}),\\
&J_{2,9}=-(k_m+\mu_{m1}+\alpha_m), && J_{2,10}=-(\delta+\mu_{m1}+\alpha_m), && J_{2,11}=-(\mu_{m1}+\alpha_m+\zeta_{m1}),\\
&J_{2,12}=-\mu_{m2}, && J_{2,13}=-(\delta+\mu_{m2}), && J_{2,14}=-(\mu_{m2}+\zeta_{m2}).
\end{aligned}$$

The characteristic equation of $J|_{\bar{E}}$ is

$$\begin{aligned}
&(\lambda+\mu_{f1}+\alpha_f)(\lambda+k_f+\delta+\mu_{f1}+\alpha_f)(\lambda-J_{2,3})(\lambda-J_{2,4})(\lambda-J_{2,5})(\lambda-J_{2,6})\\
&(\lambda-J_{2,7})(\lambda-J_{2,8})(\lambda-J_{2,11})(\lambda-J_{2,12})(\lambda-J_{2,13})(\lambda-J_{2,14})(\lambda+\mu_{m1}+\alpha_m)\\
&(\lambda+k_m+\delta+\mu_{m1}+\alpha_m)|\lambda\boldsymbol{I}-(F-V)|=0
\end{aligned}$$

Obviously, the eigenvalues of the matrix $J|_{\bar{E}}$ include $-(\mu_{f1}+\alpha_f)$, $-(k_f+\delta+\mu_{f1}+\alpha_f)$, $-(\mu_{m1}+\alpha_m)$, $-(k_m+\delta+\mu_{m1}+\alpha_m)$, $J_{2,3}$, $J_{2,4}$, $J_{2,5}$, $J_{2,6}$, $J_{2,7}$, $J_{2,8}$, $J_{2,11}$, $J_{2,12}$, $J_{2,13}$, $J_{2,14}$ and the roots of equation $|\lambda\boldsymbol{I}-(F-V)|=0$. According to Theorem 2 in the reference [2], $s(F-V)<0$ if and only if $\mathcal{R}_0<1$, and $s(F-V)>0$ if and only if $\mathcal{R}_0>1$, where $s(F-V)>0$ is spectral bound of $F-V$. In addition, except for the roots of equation $|\lambda\boldsymbol{I}-(F-V)|=0$, other eigenvalues of matrix $J|_{\bar{E}}$ are less than 0. Hence, if $\mathcal{R}_0<1$, $\bar{E}$ is globally asymptotically stable. If $\mathcal{R}_0>1$, $\bar{E}$ is unstable.

Appendix 3. Proof of Theorem 3.3

Proof. Let $x(t,x(0))$ be a solution of system (2.1)-(2.4) with initial value $x(0)$.

First, we prove that there is a $\bar{l} > 0$ such that

$$\limsup_{t\to+\infty} \mathrm{d}(x(t,x(0)),\bar{E}) \geq \bar{l}, x(0) \in \mathbb{D}_1,$$

where $\mathrm{d}(x,\bar{E})$ denotes the distance between x and $\bar{E}$. Suppose that

$$\limsup_{t\to+\infty} \mathrm{d}(x(t,x(0)),\bar{E}) < \bar{l}, x(0) \in \mathbb{D}_1$$

for some solutions $x(t,x(0))$ of system (2.1)-(2.4) with initial value $x(0) \in \mathbb{D}_1$. Then, there exists a $t_0 > 0$ such that for $t > t_0$

$$\begin{aligned} &\bar{S}^g_{fi} - \bar{l} < S^g_{fi}(t) < \bar{S}^g_{fi} + \bar{l}, \quad \bar{S}^g_{mi} - \bar{l} < S^g_{mi}(t) < \bar{S}^g_{mi} + \bar{l}, \\ &0 < N_{fi}(t) < \bar{N}_{fi} + 12\bar{l}, \qquad 0 < N_{mi}(t) < \bar{N}_{mi} + 5\bar{l}, \end{aligned}$$

where $g \in \{u, v\}$ and $i \in \{1, 2\}$. Therefore, when $t > t_0$, the following expressions hold:

$$\begin{aligned} &\bar{s}^u_{f1} = \frac{\bar{S}^u_{f1} - \bar{l}}{\bar{N}_{f1} + 12\bar{l}} \leq s^u_{f1}, \quad \bar{s}^v_{f1} = \frac{\bar{S}^v_{f1} - \bar{l}}{\bar{N}_{f1} + 12\bar{l}} \leq s^v_{f1}, \quad \bar{s}^u_{f2} = \frac{\bar{S}^u_{f2} - \bar{l}}{\bar{N}_{f2} + 12\bar{l}} \leq s^u_{f2}, \\ &\bar{s}^v_{f2} = \frac{\bar{S}^v_{f2} - \bar{l}}{\bar{N}_{f2} + 12\bar{l}} \leq s^v_{f2}, \quad \bar{s}^u_{m1} = \frac{\bar{S}^u_{m1} - \bar{l}}{\bar{N}_{m1} + 5\bar{l}} \leq s^u_{m1}, \quad \bar{s}^v_{m1} = \frac{\bar{S}^v_{m1} - \bar{l}}{\bar{N}_{m1} + 5\bar{l}} \leq s^v_{m1}, \\ &\bar{s}^u_{m2} = \frac{\bar{S}^v_{m2} - \bar{l}}{\bar{N}_{m2} + 5\bar{l}} \leq s^u_{m2}, \quad \bar{s}^v_{m2} = \frac{\bar{S}^v_{m2} - \bar{l}}{\bar{N}_{m2} + 5\bar{l}} \leq s^v_{m2}. \end{aligned}$$

Let

$$F_{\bar{l}} = \begin{bmatrix} \boldsymbol{O} & \boldsymbol{O} & \boldsymbol{P}_{\bar{l}} \\ \boldsymbol{O} & \boldsymbol{O} & \boldsymbol{Q}_{\bar{l}} \\ \boldsymbol{W}_{\bar{l}} & \boldsymbol{Z}_{\bar{l}} & \boldsymbol{O} \end{bmatrix},$$

where

$$\boldsymbol{P}_{\bar{l}} = \begin{bmatrix} \bar{f}_{11} & \bar{f}_{11} & \bar{f}_{21} & \bar{f}_{21} \\ 0&0&0&0 \\ 0&0&0&0 \\ 0&0&0&0 \\ \bar{f}_{12} & \bar{f}_{12} & \bar{f}_{22} & \bar{f}_{22} \\ 0&0&0&0 \\ 0&0&0&0 \\ 0&0&0&0 \end{bmatrix}, \quad \boldsymbol{Q}_{\bar{l}} = \begin{bmatrix} \bar{f}_{31} & \bar{f}_{31} & \bar{f}_{41} & \bar{f}_{41} \\ 0&0&0&0 \\ 0&0&0&0 \\ 0&0&0&0 \\ \bar{f}_{32} & \bar{f}_{32} & \bar{f}_{42} & \bar{f}_{42} \\ 0&0&0&0 \\ 0&0&0&0 \\ 0&0&0&0 \end{bmatrix},$$

$$\boldsymbol{W}_{\bar{l}} = \begin{bmatrix} \bar{f}_{51} & \bar{f}_{51} & \bar{f}_{51} & \bar{f}_{51} & \bar{f}_{51} & \bar{f}_{51} & \bar{f}_{51} & \bar{f}_{51} \\ \bar{f}_{52} & \bar{f}_{52} & \bar{f}_{52} & \bar{f}_{52} & \bar{f}_{52} & \bar{f}_{52} & \bar{f}_{52} & \bar{f}_{52} \\ \bar{f}_{71} & \bar{f}_{71} & \bar{f}_{71} & \bar{f}_{71} & \bar{f}_{71} & \bar{f}_{71} & \bar{f}_{71} & \bar{f}_{71} \\ \bar{f}_{72} & \bar{f}_{72} & \bar{f}_{72} & \bar{f}_{72} & \bar{f}_{72} & \bar{f}_{72} & \bar{f}_{72} & \bar{f}_{72} \end{bmatrix}, \quad \boldsymbol{Z}_{\bar{l}} = \begin{bmatrix} \bar{f}_{61} & \bar{f}_{61} & \bar{f}_{61} & \bar{f}_{61} & \bar{f}_{61} & \bar{f}_{61} & \bar{f}_{61} & \bar{f}_{61} \\ \bar{f}_{62} & \bar{f}_{62} & \bar{f}_{62} & \bar{f}_{62} & \bar{f}_{62} & \bar{f}_{62} & \bar{f}_{62} & \bar{f}_{62} \\ \bar{f}_{81} & \bar{f}_{81} & \bar{f}_{81} & \bar{f}_{81} & \bar{f}_{81} & \bar{f}_{81} & \bar{f}_{81} & \bar{f}_{81} \\ \bar{f}_{82} & \bar{f}_{82} & \bar{f}_{82} & \bar{f}_{82} & \bar{f}_{82} & \bar{f}_{82} & \bar{f}_{82} & \bar{f}_{82} \end{bmatrix},$$

in which

$$\bar{f}_{11} = \beta_m c_{m11} \bar{s}^u_{f1}, \qquad \bar{f}_{12} = (1 - \varepsilon_{f1})\beta_m c_{m11} \bar{s}^v_{f1}, \quad \bar{f}_{21} = \beta_m c_{m21} \bar{s}^u_{f1},$$

$\bar{f}_{22} = (1-\varepsilon_{f1})\beta_m c_{m21}\bar{s}^v_{f1}$, $\bar{f}_{31} = \beta_m c_{m12}\bar{s}^u_{f2}$, $\bar{f}_{32} = (1-\varepsilon_{f2})\beta_m c_{m12}\bar{s}^v_{f2}$, $\bar{f}_{41} = \beta_m c_{m22}\bar{s}^u_{f2}$, $\bar{f}_{42} = (1-\varepsilon_{f2})\beta_m c_{m22}\bar{s}^v_{f2}$, $\bar{f}_{51} = \beta_f c_{f11}\bar{s}^u_{m1}$, $\bar{f}_{52} = (1-\varepsilon_{m1})\beta_f c_{f11}\bar{s}^v_{m1}$, $\bar{f}_{61} = \beta_f c_{f21}\bar{s}^u_{m1}$, $\bar{f}_{62} = (1-\varepsilon_{m1})\beta_f c_{f21}\bar{s}^v_{m1}$, $\bar{f}_{71} = \beta_f c_{f12}\bar{s}^u_{m2}$, $\bar{f}_{72} = (1-\varepsilon_{m2})\beta_f c_{f12}\bar{s}^v_{m2}$, $\bar{f}_{81} = \beta_f c_{f22}\bar{s}^u_{m2}$, $\bar{f}_{82} = (1-\varepsilon_{m2})\beta_f c_{f22}\bar{s}^v_{m2}$.

Then, from system (2.1)-(2.4), we get

$$\frac{d\mathbf{y}^T}{dt} \geq (F_{\bar{l}} - V)\mathbf{y}^T \tag{S3.1}$$

for $t > t_0$, where $\mathbf{y}^T$ is transpose of $\mathbf{y}$. Consider the following linear system

$$\frac{d\bar{\mathbf{y}}^T}{dt} = (F_{\bar{l}} - V)\bar{\mathbf{y}}^T. \tag{S3.2}$$

Let $\bar{s}(\bar{l})$ be spectral bound of $F_{\bar{l}} - V$. Since the non-diagonal elements for the matrix $F_{\bar{l}} - V$ are non-negative and its corresponding directed graph is strongly connected, system (S3.2) is quasi-monotone and irreducible. Further, by Theorem 5.5.1 in the reference [3], $\bar{s}(\bar{l})$ is a root of

$$|\Lambda \boldsymbol{I} - F_{\bar{l}}V^{-1}| = 0.$$

Let $\bar{\mathcal{R}}_0(\bar{l}) = \rho(F_{\bar{l}}V^{-1}) = \max\{|\Lambda| \mid |\Lambda \boldsymbol{I} - F_{\bar{l}}V^{-1}| = 0\}$. Obviously, $\bar{\mathcal{R}}_0(0) = \mathcal{R}_0 > 1$. By Theorem 2 in the reference [2], when $\mathcal{R}_0 > 1$, $\bar{s}(0) > 0$. Then, due to the continuity of $\bar{s}$, $\bar{s}(\bar{l}) > 0$ for $\bar{l} > 0$ sufficiently small. Further, by Theorem 5.5.1 in the reference [3], system (S3.2) has a solution $\bar{\mathbf{y}}^T = e^{\bar{s}(\bar{l})t}\hat{\mathbf{y}}$, where $\hat{\mathbf{y}}$ is the positive eigenvector for $\bar{s}(\bar{l})$. Further, from (S3.1), comparison theorem implies that there exists a $l_1 > 0$ such that $\mathbf{y}^T \geq l_1 e^{\bar{s}(\bar{l})t}\hat{\mathbf{y}}$ for $t > t_0$. Hence,

$$\lim_{t\to+\infty} \tilde{y}(t) = \infty, \forall \tilde{y} \in \mathrm{Y}.$$

This is a contradiction. Therefore, $W^s(\bar{E}) \bigcap \mathbb{D}_1 = \varnothing$, where $W^s(\bar{E})$ is the stable manifold of $\bar{E}$.

Let $\Omega = \bigcup_{x(0)\in\mathbb{D}_2} \omega(x(0))$, where $\omega(x(0))$ is the omega limit set of orbit $x(t, x(0))$. If $x(0) \in \mathbb{D}_2$, $\tilde{y}(t) \equiv 0$ for all $\tilde{y} \in \mathrm{Y}$. Then, $\lim_{t\to\infty} x(t) = \bar{E}$. Hence, $\Omega = \bar{E}$ and there is no cycles in $\mathbb{D}_2$ from $\bar{E}$ to $\bar{E}$. Finally, by Theorem 3 in the reference [4], system (2.1)-(2.4) is uniformly persistent when $\mathcal{R}_0 > 1$.

Appendix 4. Annual number of cervical cancer cases and deaths

Annual number of cervical cancer cases and deaths from 2006 to 2016 can be seen in Table S1. The data are directly obtained or estimated based on the data in the references [5, 6, 7, 8, 9, 10, 11, 12, 13, 14, 15, 16]. In China, the proportion of HPV16 infection among cervical cancer patients is about 75.55%; the proportion of HPV18 infection is about 10.835% [17, 18]. Therefore, when fitting the data of HPV16, we multiply the data in Table S1 by 0.7555; similarly, when fitting the data of HPV18, we multiply by 0.10835; when fitting the total data of HPV16 and HPV18, we multiply by 0.86385. In addition, the cumulative cases and cumulative deaths of cervical cancer related to HPV16 and HPV18 can be obtained by simple summation calculation, as shown in Fig. 4 (see text) and Figs. S3 and S7.

Table S1: Annual number of cervical cancer cases and deaths

Year	New cases			New deaths		
	$15 \sim 44$ years old	≥ 45 years old	≥ 15 years old	$15 \sim 44$ years old	≥ 45 years old	≥ 15 years old
2006	29880	36658	66538	3777	13932	17709
2007	31601	44839	75756	3565	14275	17840
2008	32199	48346	80545	2156	15243	17399
2009	36550	48504	85054	2247	16791	19038
2010	32376	44508	76884	4043	17583	21626
2011	34542	53440	87982	4559	18816	23375
2012	38094	60906	99000	5210	19790	25000
2013	33913	67087	101000	4459	21541	26000
2014	26800	75200	102000	4400	26600	31000
2015	26646	84354	111000	4695	29105	33800
2016	26980	92320	119300	4630	32570	37200

Appendix 5. Some known parameters

(1) The values of parameters A_f, A_m, μ_{f1}, μ_{m1}, μ_{f2}, μ_{m2}, α_f and α_m over time can be obtained from the population estimation data of United Nations Department of Economic and Social Affairs Population Division [19], see Fig. S1.

(2) Assume that $\sigma^u_{f1} = \sigma^u_{f2}$. The screening rate of cervical cancer from 2006 to 2020 can be seen from the *YEAR BOOK OF HEALTH IN THE PEOPLE'S REPUBLIC OF CHINA* on the official website of the National Health Commission of the People's Republic of China (http://www.nhc.gov.cn/), as shown in Fig. S2.

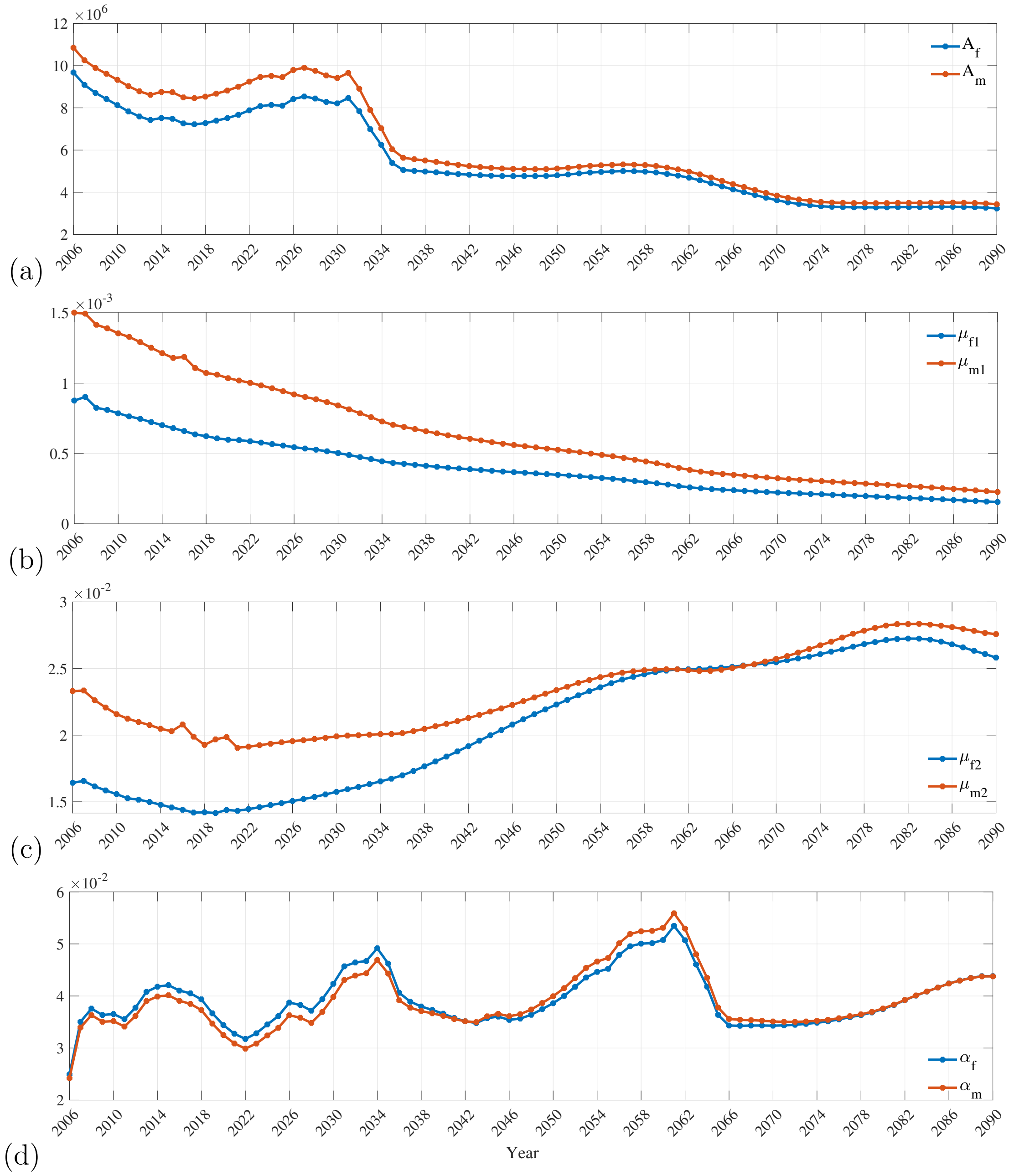

Fig. S1: Variation of parameters A_f, A_m, μ_{f1}, μ_{m1}, μ_{f2}, μ_{m2}, α_f and α_m over time.

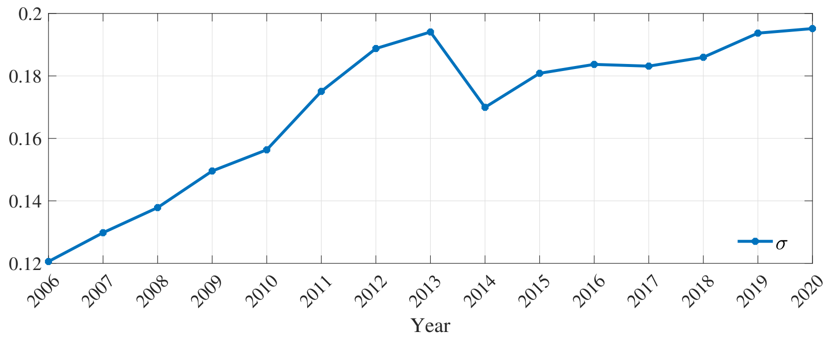

Fig. S2: Variation of parameters σ^u_{f1} and σ^u_{f2} over time.

Appendix 6. Initial value

(1) We take the population of the two age groups in 2006 as the initial values of N_{f1}, N_{f2}, N_{m1}, and N_{m2} [19].

$$N_{f1}(0) = 331723224, \quad N_{f2}(0) = 190437869,$$
$$N_{m1}(0) = 350801169, \quad N_{m2}(0) = 183501173.$$

(2) In addition to fitting the unknown parameters, we also need to fit the initial values $C^u_{f1}(0)$ and $C^u_{f2}(0)$. The initial values of other variables are obtained by assuming that the system is in equilibrium at the initial moment (that is, these values can make the right-hand sides of system (2.1)-(2.4) equal to 0). Since vaccinated compartments are not considered when fitting the data from 2006 to 2016, only the initial values of unvaccinated compartments need to be considered here. In 2016, the ratio of male and female tumor incidences attributed to HPV infection was 7654 : 117118 [20]. We therefore assume that the proportion of infected men among men is equal to the proportion of all infected women among women multiplied by 7654/117118. In summary, for the female variables, the relationship between the initial values is as follows:

$$\begin{aligned}
H^u_{f1}(0) &= \frac{[\Lambda + (1-\Lambda)\sigma^u_{f1}(0) + \mu_{f1}(0) + \alpha_f(0)]C^u_{f1}(0)}{\xi^u_{f1}},\\
L^u_{f1}(0) &= \frac{(\xi^u_{f1} + \sigma^u_{f1}(0) + \mu_{f1}(0) + \alpha_f(0))H^u_{f1}(0)}{(1-e^u_{f1})\eta},\\
I^u_{f1}(0) &= \frac{(\eta + \sigma^u_{f1}(0) + \mu_{f1}(0) + \alpha_f(0))L^u_{f1}(0)}{(1-r^u_{f1})\rho},\\
D_{f1}(0) &= \frac{\sigma^u_{f1}(0)(L^u_{f1}(0) + H^u_{f1}(0)) + [\Lambda + (1-\Lambda)\sigma^u_{f1}(0)]C^u_{f1}(0)}{\gamma_{f1} + d_{f1} + \mu_{f1}(0) + \alpha_f(0)},\\
R_{f1}(0) &= \frac{\gamma_{f1}D_{f1}(0) + r^u_{f1}\rho I^u_{f1}(0) + e^u_{f1}\eta L^u_{f1}(0)}{\mu_{f1}(0) + \alpha_f(0) + \zeta_{f1}},\\
S^u_{f1}(0) &= N_{f1}(0) - I^u_{f1}(0) - L^u_{f1}(0) - H^u_{f1}(0) - C^u_{f1}(0) - D_{f1}(0) - R_{f1}(0),\\
H^u_{f2}(0) &= \frac{(\Lambda + (1-\Lambda)\sigma^u_{f2}(0) + \mu_{f2}(0))C^u_{f2}(0) - \alpha_f(0)C^u_{f1}(0)}{\xi^u_{f2}},\\
L^u_{f2}(0) &= \frac{(\xi^u_{f2} + \sigma^u_{f2}(0) + \mu_{f2}(0))H^u_{f2}(0) - \alpha_f(0)H^u_{f1}(0)}{(1-e^u_{f2})\eta},\\
I^u_{f2}(0) &= \frac{(\eta + \sigma^u_{f2}(0) + \mu_{f2}(0))L^u_{f2}(0) - \alpha_f(0)L^u_{f1}(0)}{(1-r^u_{f2})\rho},
\end{aligned}$$

$$
\begin{aligned}
D_{f2}(0) &= \frac{\sigma^u_{f2}(0)(L^u_{f2}(0)+H^u_{f2}(0))+[\Lambda+(1-\Lambda)\sigma^u_{f2}(0)]C^u_{f2}(0)+\alpha_f(0)D_{f1}(0)}{\gamma_{f2}+d_{f2}+\mu_{f2}(0)},\\
R_{f2}(0) &= \frac{\alpha_f(0)R_{f1}(0)+\gamma_{f2}D_{f2}(0)+r^u_{f2}\rho I^u_{f2}(0)+e^u_{f2}\eta L^u_{f2}(0)}{\mu_{f2}(0)+\zeta_{f2}},\\
S^u_{f2}(0) &= N_{f2}(0)-I^u_{f2}(0)-L^u_{f2}(0)-H^u_{f2}(0)-C^u_{f2}(0)-D_{f2}(0)-R_{f2}(0),\\
I^u_{m1}(0) &= N_{m1}(0)\frac{I^u_{f1}(0)+L^u_{f1}(0)+H^u_{f1}(0)+C^u_{f1}(0)+D_{f1}(0)}{N_{f1}(0)}\frac{7654}{117118},\\
I^u_{m2}(0) &= N_{m2}(0)\frac{I^u_{f2}(0)+L^u_{f2}(0)+H^u_{f2}(0)+C^u_{f2}(0)+D_{f2}(0)}{N_{f2}(0)}\frac{7654}{117118},\\
R_{m1}(0) &= \frac{\omega^u_{m1}I^u_{m1}(0)}{\mu_{m1}(0)+\alpha_m(0)+\zeta_{m1}},\\
R_{m2}(0) &= \frac{\alpha_m(0)R_{m1}(0)+\omega^u_{m2}I^u_{m2}(0)}{\mu_{m2}(0)+\zeta_{m2}},\\
S^u_{m1}(0) &= N_{m1}(0)-I^u_{m1}(0)-R_{m1}(0),\\
S^u_{m2}(0) &= N_{m2}(0)-I^u_{m2}(0)-R_{m2}(0),
\end{aligned}
$$

where $\mu_{f1}(0)$, $\mu_{m1}(0)$, $\mu_{f2}(0)$, $\mu_{m2}(0)$, $\alpha_f(0)$, $\alpha_m(0)$, $\sigma^u_{f1}(0)$ and $\sigma^u_{f2}(0)$ correspond to values in 2006.

(3) The initial values of the variables $\tilde{Y}_{c1}$, $\tilde{Y}_{c2}$ and $\tilde{Y}_{c3}$ are the corresponding number of cases in 2006, and the initial values of $\tilde{Y}_{d1}$, $\tilde{Y}_{d2}$ and $\tilde{Y}_{d3}$ are the corresponding deaths in 2006.

Appendix 7. Data fitting results and parameter uncertainty analysis

7.1 Data fitting results of HPV16 and parameter uncertainty analysis

(1) For HPV16 [21, 22],

$$\zeta_{f1} = \zeta_{f2} = 0.041 \ (95\%CI \ [0.032, 0.054]),$$

$$\zeta_{m1} = \zeta_{m2} = 0.076 \ (95\%CI \ [0.0429, 0.1195]).$$

(2) All fitting results:

- Fig. S3: Fitting results regarding the number of HPV16-related cervical cancer cases and deaths from 2006 to 2016.
- Fig. S4: For HPV16, histogram of 1000 values for parameters β_{m11}, β_{m12}, β_{m21}, β_{m22}, β_{f11}, β_{f12}, β_{f21} and β_{f22}.
- Fig. S5: For HPV16, estimation results about d_{f1} and d_{f2}.
- Fig. S6: For HPV16, time-varying reproduction number.
- Table S2: For HPV16, means and 95% confidence interval of the estimated parameters and initial values.

7.2 Data fitting results of HPV18 and parameter uncertainty analysis

(1) For HPV18 [21, 22],

$$\zeta_{f1} = \zeta_{f2} = 0.029 \ (95\%CI \ [0.021, 0.045]);$$

$$\zeta_{m1} = \zeta_{m2} = 0.0621 \ (95\%CI \ [0.0225, 0.1171]).$$

(2) All fitting results:

- Fig. S7: Fitting results regarding the number of HPV18-related cervical cancer cases and deaths from 2006 to 2016.
- Fig. S8: For HPV18, histogram of 1000 values for parameters β_{m11}, β_{m12}, β_{m21}, β_{m22}, β_{f11}, β_{f12}, β_{f21} and β_{f22}.
- Fig. S9: For HPV18, estimation results about d_{f1} and d_{f2}.
- Fig. S10: For HPV18, time-varying reproduction number.
- Table S3: For HPV18, means and 95% confidence interval of the estimated parameters and initial values.

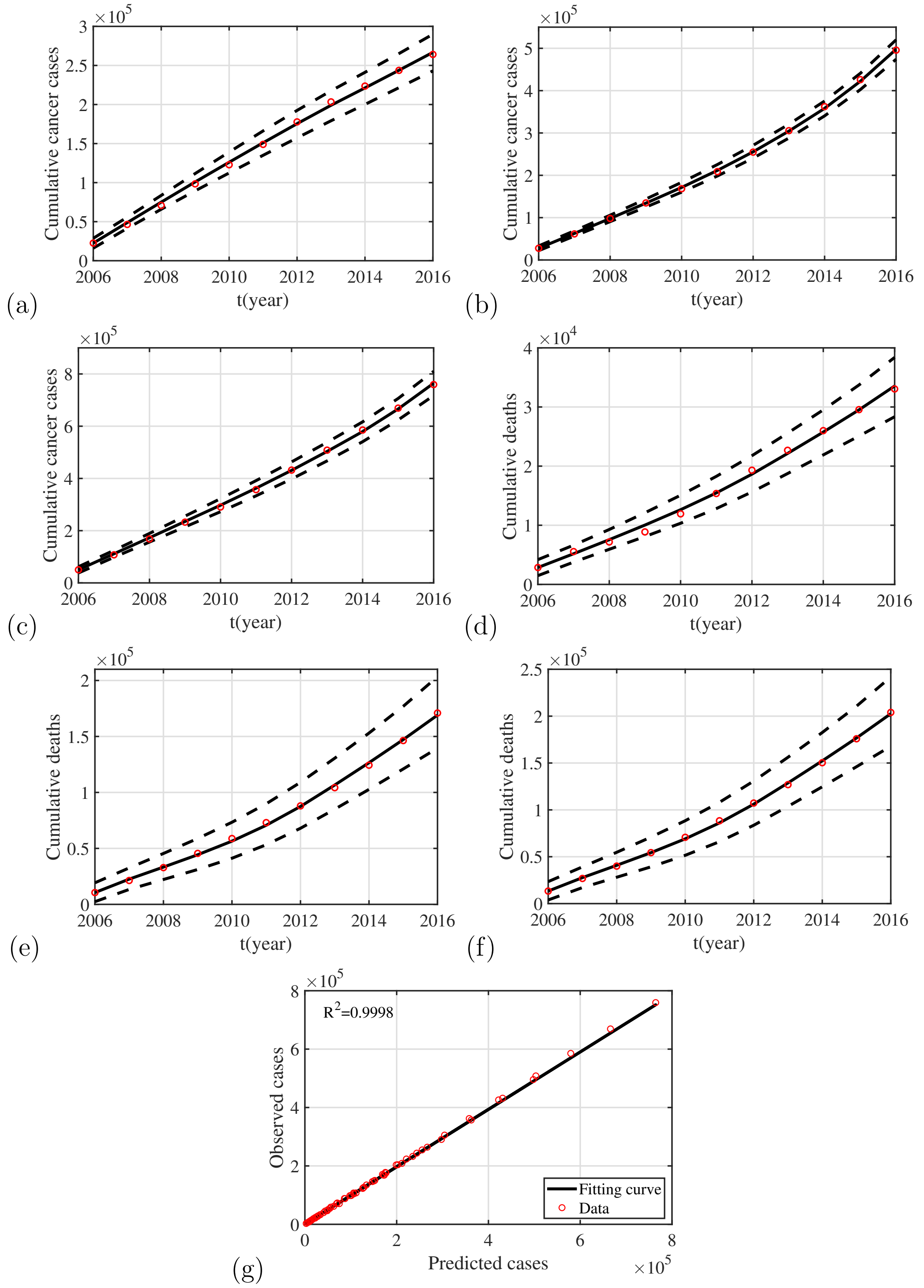

Fig. S3: Fitting results regarding the number of HPV16-related cervical cancer cases and deaths from 2006 to 2016. The red circles represent the actual cumulative number of cervical cancer cases or deaths. The black solid line represents the fitting result, and the black dashed line represents the 95% confidence interval. (a) Cumulative cases in the first age group. (b) Cumulative cases in the second age group. (c) The sum of cumulative cases in the two age groups. (d) Cumulative deaths in the first age group. (e) Cumulative deaths in the second age group. (f) The sum of cumulative deaths in the two age groups. (g) Relation between observed and predicted cases.

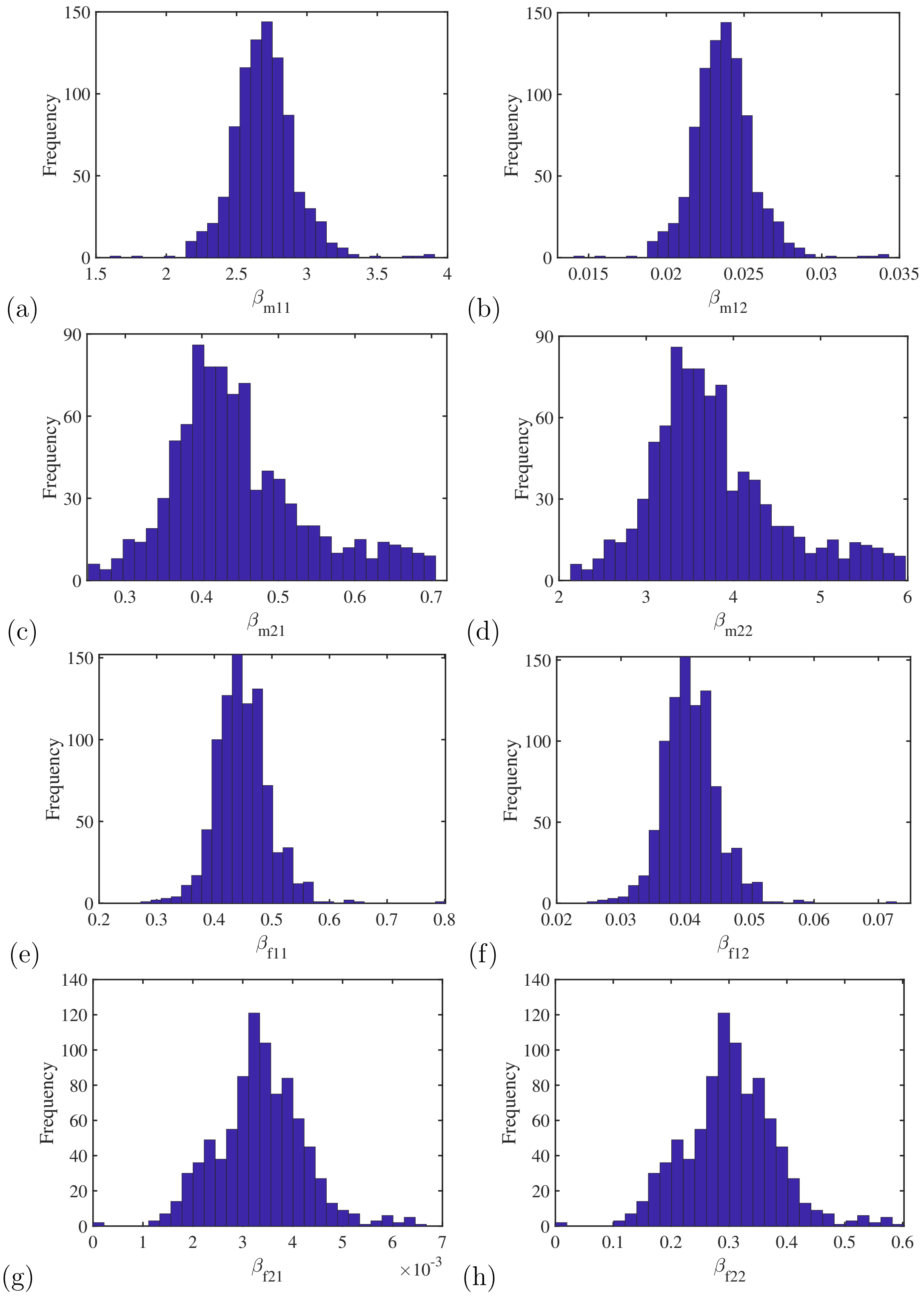

Fig. S4: For HPV16, histogram of 1000 values for parameters β_{m11}, β_{m12}, β_{m21}, β_{m22}, β_{f11}, β_{f12}, β_{f21} and β_{f22}.

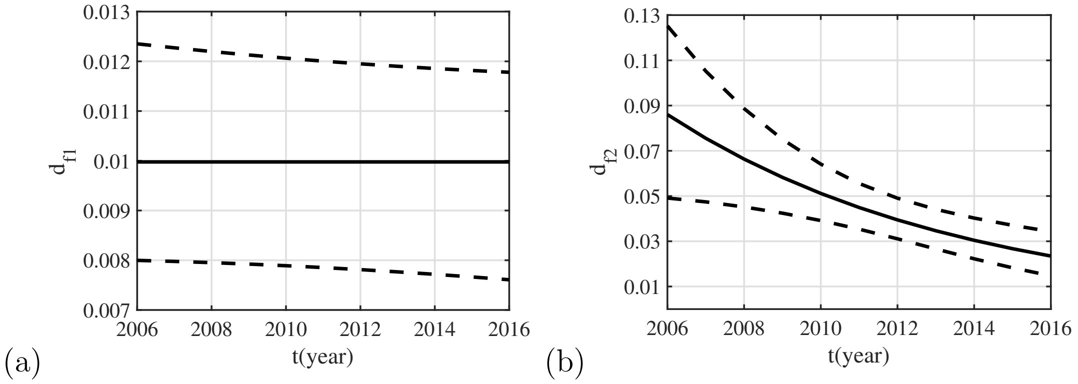

Fig. S5: For HPV16, estimation results about d_{f1} and d_{f2}. The black solid line represents the fitting result, and the black dashed line represents the 95% confidence interval.

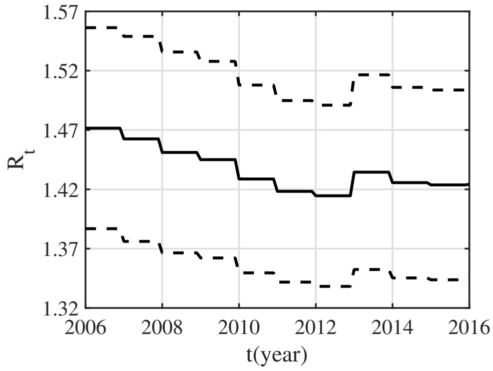

Fig. S6: For HPV16, time-varying reproduction number. The black solid line represents the fitting result, and the black dashed line represents the 95% confidence interval.

Table S2: For HPV16, means and 95% confidence interval of the estimated parameters and initial values.

Parameter or initial value	Value	95% CI
β_{m11}	2.4632	$[2.2573, 3.1338]$
β_{m12}	0.0216	$[0.0198, 0.0275]$
β_{m21}	0.4363	$[0.2721, 0.6281]$
β_{m22}	3.6948	$[2.3046, 5.3197]$
β_{f11}	0.49	$[0.3568, 0.5415]$
β_{f12}	0.0445	$[0.0324, 0.0492]$
β_{f21}	0.0033	$[0.0016, 0.0051]$
β_{f22}	0.296	$[0.1473, 0.458]$
d_{k1}	0.01	$[0.008, 0.0124]$
d_{b1}	1.4645×10^{-11}	$[3.23 \times 10^{-13}, 0.01]$
d_{k2}	0.086	$[0.05, 0.12]$
d_{b2}	0.1299	$[0.0533, 0.1994]$
$C^u_{f1}(0)$	6.6553×10^4	$[5.9691 \times 10^4, 7.4764 \times 10^4]$
$C^u_{f2}(0)$	8.3676×10^4	$[8.3462 \times 10^4, 9.6801 \times 10^4]$

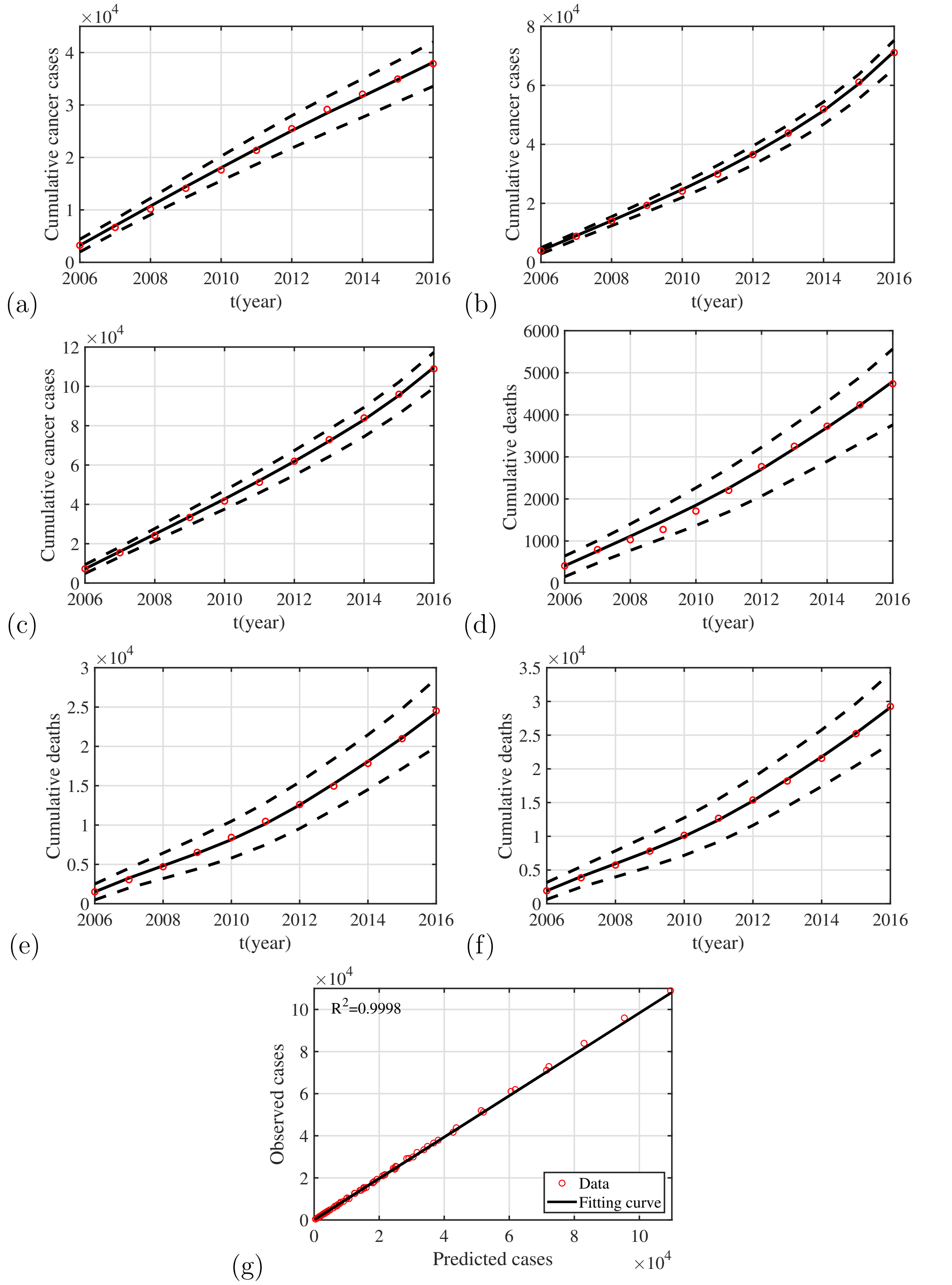

Fig. S7: Fitting results regarding the number of HPV18-related cervical cancer cases and deaths from 2006 to 2016. The red circles represent the actual cumulative number of cervical cancer cases or deaths. The black solid line represents the fitting result, and the black dashed line represents the 95% confidence interval. (a) Cumulative cases in the first age group. (b) Cumulative cases in the second age group. (c) The sum of cumulative cases in the two age groups. (d) Cumulative deaths in the first age group. (e) Cumulative deaths in the second age group. (f) The sum of cumulative deaths in the two age groups. (g) Relation between observed and predicted cases.

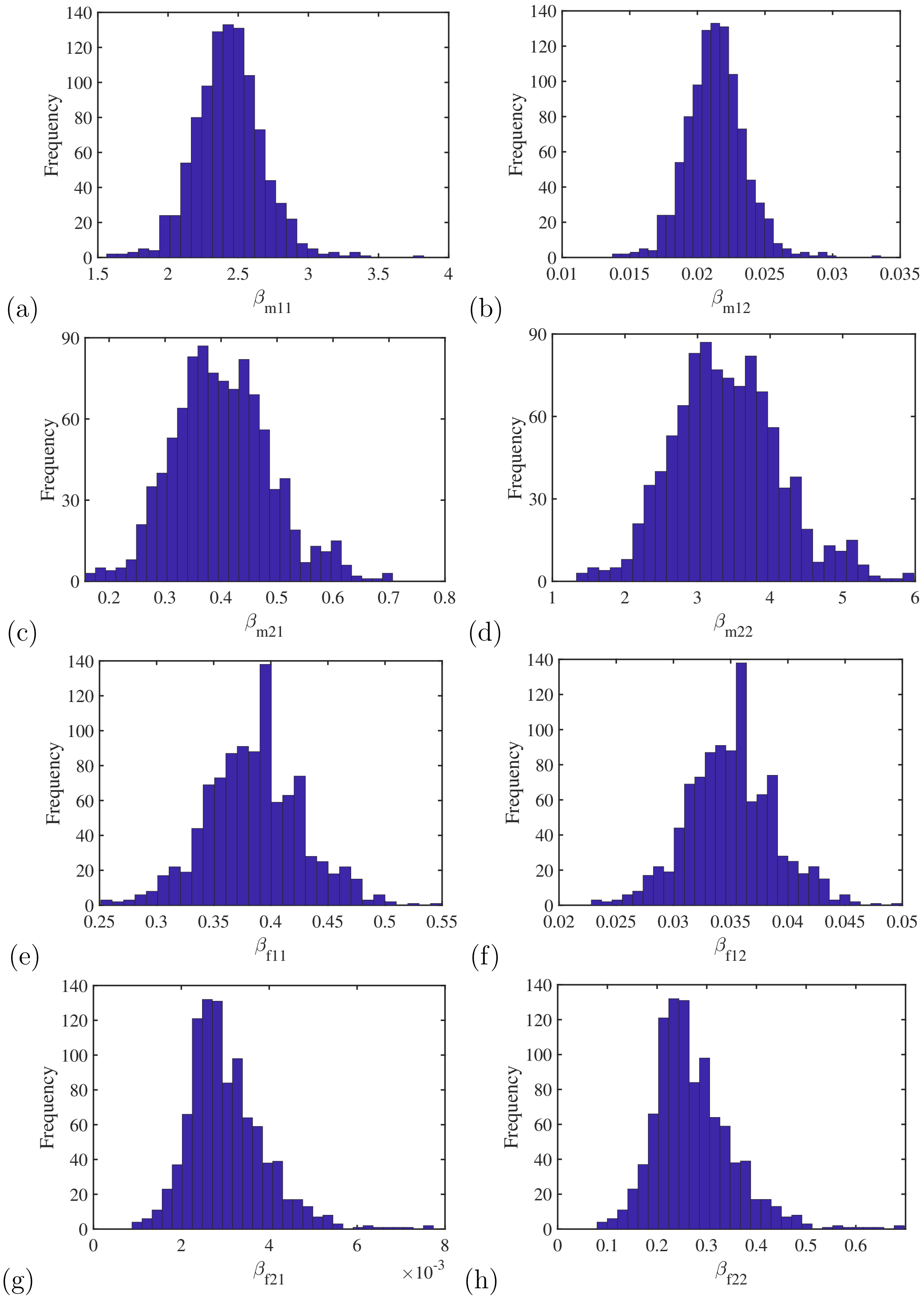

Fig. S8: For HPV18, histogram of 1000 values for parameters β_{m11}, β_{m12}, β_{m21}, β_{m22}, β_{f11}, β_{f12}, β_{f21} and β_{f22}.

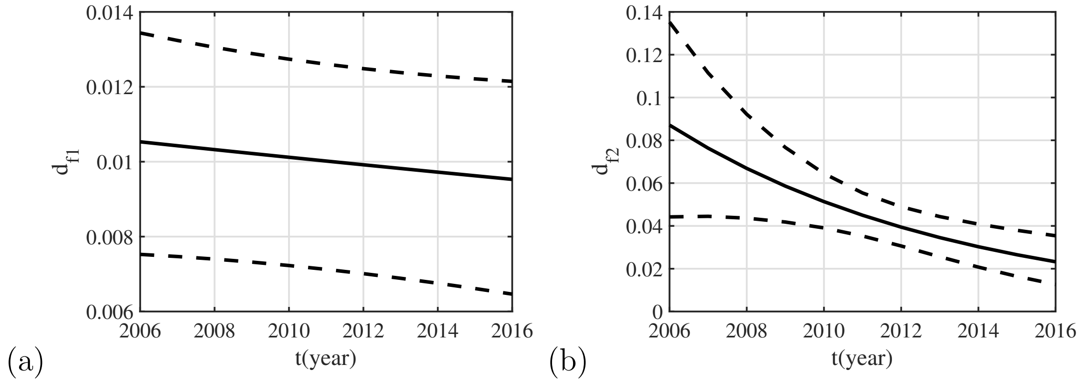

Fig. S9: For HPV18, estimation results about d_{f1} and d_{f2}. The black solid line represents the fitting result, and the black dashed line represents the 95% confidence interval.

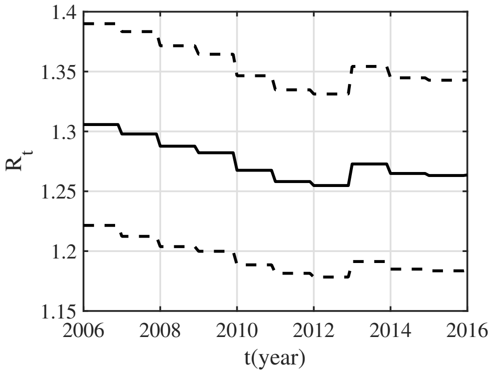

Fig. S10: For HPV18, time-varying reproduction number. The black solid line represents the fitting result, and the black dashed line represents the 95% confidence interval.

Table S3: For HPV18, means and 95% confidence interval of the estimated parameters and initial values.

Parameter or initial value	Value	95% CI
β_{m11}	2.4189	$[1.953, 2.9167]$
β_{m12}	0.0212	$[0.0171, 0.0256]$
β_{m21}	0.4196	$[0.2304, 0.5738]$
β_{m22}	3.5535	$[1.9512, 4.8601]$
β_{f11}	0.389	$[0.3035, 0.4678]$
β_{f12}	0.0353	$[0.0276, 0.0425]$
β_{f21}	0.0027	$[0.0013, 0.0048]$
β_{f22}	0.2435	$[0.1164, 0.4288]$
d_{k1}	0.0105	$[0.0075, 0.0134]$
d_{b1}	0.01	$[2.488 \times 10^{-13}, 0.02]$
d_{k2}	0.0871	$[0.0442, 0.1352]$
d_{b2}	0.1321	$[0.046, 0.2165]$
$C^u_{f1}(0)$	9.7364×10^3	$[8.2169 \times 10^3, 1.0923 \times 10^4]$
$C^u_{f2}(0)$	1.2987×10^4	$[1.1406 \times 10^4, 1.4177 \times 10^4]$

Appendix 8. System (S8.1)-(S8.5)

$$
\begin{aligned}
\frac{\mathrm{d}S^u_{f1}}{\mathrm{d}t} &= A_f(1-\phi_f(t)) - \lambda_{m1}S^u_{f1} - k_f(t)S^u_{f1} + \delta S^v_{f1} - \mu_{f1}S^u_{f1} - \alpha_f S^u_{f1} + \zeta_{f1}R_{f1},\\
\frac{\mathrm{d}I^u_{f1}}{\mathrm{d}t} &= \lambda_{m1}S^u_{f1} - \rho I^u_{f1} - \mu_{f1}I^u_{f1} - \alpha_f I^u_{f1},\\
\frac{\mathrm{d}L^u_{f1}}{\mathrm{d}t} &= (1-r^u_{f1})\rho I^u_{f1} - \eta L^u_{f1} - \sigma^u_{f1}(t)L^u_{f1} - \mu_{f1}L^u_{f1} - \alpha_f L^u_{f1},\\
\frac{\mathrm{d}H^u_{f1}}{\mathrm{d}t} &= (1-e^u_{f1})\eta L^u_{f1} - \xi^u_{f1}H^u_{f1} - \sigma^u_{f1}(t)H^u_{f1} - \mu_{f1}H^u_{f1} - \alpha_f H^u_{f1},\\
\frac{\mathrm{d}C^u_{f1}}{\mathrm{d}t} &= \xi^u_{f1}H^u_{f1} - \Lambda C^u_{f1} - (1-\Lambda)\sigma^u_{f1}(t)C^u_{f1} - \mu_{f1}C^u_{f1} - \alpha_f C^u_{f1},\\
\frac{\mathrm{d}S^v_{f1}}{\mathrm{d}t} &= A_f\phi_f(t) - (1-\varepsilon_{f1})\lambda_{m1}S^v_{f1} + k_f(t)S^u_{f1} - \delta S^v_{f1} - \mu_{f1}S^v_{f1} - \alpha_f S^v_{f1},\\
\frac{\mathrm{d}I^v_{f1}}{\mathrm{d}t} &= (1-\varepsilon_{f1})\lambda_{m1}S^v_{f1} - \rho I^v_{f1} - \mu_{f1}I^v_{f1} - \alpha_f I^v_{f1},\\
\frac{\mathrm{d}L^v_{f1}}{\mathrm{d}t} &= (1-r^v_{f1})\rho I^v_{f1} - \eta L^v_{f1} - \sigma^v_{f1}(t)L^v_{f1} - \mu_{f1}L^v_{f1} - \alpha_f L^v_{f1},\\
\frac{\mathrm{d}H^v_{f1}}{\mathrm{d}t} &= (1-e^v_{f1})\eta L^v_{f1} - \xi^v_{f1}H^v_{f1} - \sigma^v_{f1}(t)H^v_{f1} - \mu_{f1}H^v_{f1} - \alpha_f H^v_{f1},\\
\frac{\mathrm{d}C^v_{f1}}{\mathrm{d}t} &= \xi^v_{f1}H^v_{f1} - \Lambda C^v_{f1} - (1-\Lambda)\sigma^v_{f1}(t)C^v_{f1} - \mu_{f1}C^v_{f1} - \alpha_f C^v_{f1},\\
\frac{\mathrm{d}D_{f1}}{\mathrm{d}t} &= \sigma^u_{f1}(t)(L^u_{f1} + H^u_{f1}) + [\Lambda + (1-\Lambda)\sigma^u_{f1}(t)]C^u_{f1} + \sigma^v_{f1}(t)(L^v_{f1} + H^v_{f1})\\
&\quad + [\Lambda + (1-\Lambda)\sigma^v_{f1}(t)]C^v_{f1} - (\gamma_{f1} + d_{f1} + \mu_{f1} + \alpha_f)D_{f1},\\
\frac{\mathrm{d}R_{f1}}{\mathrm{d}t} &= \gamma_{f1}D_{f1} + r^u_{f1}\rho I^u_{f1} + e^u_{f1}\eta L^u_{f1} + r^v_{f1}\rho I^v_{f1} + e^v_{f1}\eta L^v_{f1} - \mu_{f1}R_{f1} - \alpha_f R_{f1} - \zeta_{f1}R_{f1},
\end{aligned}
\tag{S8.1}
$$

$$
\begin{aligned}
\frac{\mathrm{d}S^u_{f2}}{\mathrm{d}t} &= \alpha_f S^u_{f1} - \lambda_{m2}S^u_{f2} + \delta S^v_{f2} - \mu_{f2}S^u_{f2} + \zeta_{f2}R_{f2},\\
\frac{\mathrm{d}I^u_{f2}}{\mathrm{d}t} &= \alpha_f I^u_{f1} + \lambda_{m2}S^u_{f2} - \rho I^u_{f2} - \mu_{f2}I^u_{f2},\\
\frac{\mathrm{d}L^u_{f2}}{\mathrm{d}t} &= \alpha_f L^u_{f1} + (1-r^u_{f2})\rho I^u_{f2} - \eta L^u_{f2} - \sigma^u_{f2}(t)L^u_{f2} - \mu_{f2}L^u_{f2},\\
\frac{\mathrm{d}H^u_{f2}}{\mathrm{d}t} &= \alpha_f H^u_{f1} + (1-e^u_{f2})\eta L^u_{f2} - \xi^u_{f2}H^u_{f2} - \sigma^u_{f2}(t)H^u_{f2} - \mu_{f2}H^u_{f2},\\
\frac{\mathrm{d}C^u_{f2}}{\mathrm{d}t} &= \alpha_f C^u_{f1} + \xi^u_{f2}H^u_{f2} - \Lambda C^u_{f2} - (1-\Lambda)\sigma^u_{f2}(t)C^u_{f2} - \mu_{f2}C^u_{f2},\\
\frac{\mathrm{d}S^v_{f2}}{\mathrm{d}t} &= \alpha_f S^v_{f1} - (1-\varepsilon_{f2})\lambda_{m2}S^v_{f2} - \delta S^v_{f2} - \mu_{f2}S^v_{f2},\\
\frac{\mathrm{d}I^v_{f2}}{\mathrm{d}t} &= \alpha_f I^v_{f1} + (1-\varepsilon_{f2})\lambda_{m2}S^v_{f2} - \rho I^v_{f2} - \mu_{f2}I^v_{f2},\\
\frac{\mathrm{d}L^v_{f2}}{\mathrm{d}t} &= \alpha_f L^v_{f1} + (1-r^v_{f2})\rho I^v_{f2} - \eta L^v_{f2} - \sigma^v_{f2}(t)L^v_{f2} - \mu_{f2}L^v_{f2},\\
\frac{\mathrm{d}H^v_{f2}}{\mathrm{d}t} &= \alpha_f H^v_{f1} + (1-e^v_{f2})\eta L^v_{f2} - \xi^v_{f2}H^v_{f2} - \sigma^v_{f2}(t)H^v_{f2} - \mu_{f2}H^v_{f2},\\
\frac{\mathrm{d}C^v_{f2}}{\mathrm{d}t} &= \alpha_f C^v_{f1} + \xi^v_{f2}H^v_{f2} - \Lambda C^v_{f2} - (1-\Lambda)\sigma^v_{f2}(t)C^v_{f2} - \mu_{f2}C^v_{f2},\\
\frac{\mathrm{d}D_{f2}}{\mathrm{d}t} &= \alpha_f D_{f1} + \sigma^u_{f2}(t)(L^u_{f2} + H^u_{f2}) + [\Lambda + (1-\Lambda)\sigma^u_{f2}(t)]C^u_{f2} + \sigma^v_{f2}(t)(L^v_{f2} + H^v_{f2})
\end{aligned}
\tag{S8.2}
$$

$$
\begin{aligned}
&+[\Lambda+(1-\Lambda)\sigma_{f2}^{v}(t)]C_{f2}^{v}-(\gamma_{f2}+d_{f2}+\mu_{f2})D_{f2},\\
\frac{\mathrm{d}R_{f2}}{\mathrm{d}t}&=\alpha_f R_{f1}+\gamma_{f2}D_{f2}+r_{f2}^{u}\rho I_{f2}^{u}+e_{f2}^{u}\eta L_{f2}^{u}+r_{f2}^{v}\rho I_{f2}^{v}+e_{f2}^{v}\eta L_{f2}^{v}-\mu_{f2}R_{f2}-\zeta_{f2}R_{f2},
\end{aligned}
$$

$$
\begin{aligned}
\frac{\mathrm{d}S_{m1}^{u}}{\mathrm{d}t}&=A_m(1-\phi_m(t))-\lambda_{f1}S_{m1}^{u}-k_m(t)S_{m1}^{u}+\delta S_{m1}^{v}-\mu_{m1}S_{m1}^{u}-\alpha_m S_{m1}^{u}+\zeta_{m1}R_{m1},\\
\frac{\mathrm{d}I_{m1}^{u}}{\mathrm{d}t}&=\lambda_{f1}S_{m1}^{u}-\omega_{m1}^{u}I_{m1}^{u}-\mu_{m1}I_{m1}^{u}-\alpha_m I_{m1}^{u},\\
\frac{\mathrm{d}S_{m1}^{v}}{\mathrm{d}t}&=A_m\phi_m(t)-(1-\varepsilon_{m1})\lambda_{f1}S_{m1}^{v}+k_m(t)S_{m1}^{u}-\delta S_{m1}^{v}-\mu_{m1}S_{m1}^{v}-\alpha_m S_{m1}^{v},\\
\frac{\mathrm{d}I_{m1}^{v}}{\mathrm{d}t}&=(1-\varepsilon_{m1})\lambda_{f1}S_{m1}^{v}-\omega_{m1}^{v}I_{m1}^{v}-\mu_{m1}I_{m1}^{v}-\alpha_m I_{m1}^{v},\\
\frac{\mathrm{d}R_{m1}}{\mathrm{d}t}&=\omega_{m1}^{u}I_{m1}^{u}+\omega_{m1}^{v}I_{m1}^{v}-\mu_{m1}R_{m1}-\alpha_m R_{m1}-\zeta_{m1}R_{m1},
\end{aligned}
\tag{S8.3}
$$

$$
\begin{aligned}
\frac{\mathrm{d}S_{m2}^{u}}{\mathrm{d}t}&=\alpha_m S_{m1}^{u}-\lambda_{f2}S_{m2}^{u}+\delta S_{m2}^{v}-\mu_{m2}S_{m2}^{u}+\zeta_{m2}R_{m2},\\
\frac{\mathrm{d}I_{m2}^{u}}{\mathrm{d}t}&=\alpha_m I_{m1}^{u}+\lambda_{f2}S_{m2}^{u}-\omega_{m2}^{u}I_{m2}^{u}-\mu_{m2}I_{m2}^{u},\\
\frac{\mathrm{d}S_{m2}^{v}}{\mathrm{d}t}&=\alpha_m S_{m1}^{v}-(1-\varepsilon_{m2})\lambda_{f2}S_{m2}^{v}-\delta S_{m2}^{v}-\mu_{m2}S_{m2}^{v},\\
\frac{\mathrm{d}I_{m2}^{v}}{\mathrm{d}t}&=\alpha_m I_{m1}^{v}+(1-\varepsilon_{m2})\lambda_{f2}S_{m2}^{v}-\omega_{m2}^{v}I_{m2}^{v}-\mu_{m2}I_{m2}^{v},\\
\frac{\mathrm{d}R_{m2}}{\mathrm{d}t}&=\alpha_m R_{m1}+\omega_{m2}^{u}I_{m2}^{u}+\omega_{m2}^{v}I_{m2}^{v}-\mu_{m2}R_{m2}-\zeta_{m2}R_{m2},
\end{aligned}
\tag{S8.4}
$$

where

$$
\begin{aligned}
\lambda_{m1}&=\frac{\beta_m c_{m11}(I_{m1}^{u}+I_{m1}^{v})+\beta_m c_{m21}(I_{m2}^{u}+I_{m2}^{v})}{N_{f1}},\\
\lambda_{m2}&=\frac{\beta_m c_{m12}(I_{m1}^{u}+I_{m1}^{v})+\beta_m c_{m22}(I_{m2}^{u}+I_{m2}^{v})}{N_{f2}},\\
\lambda_{f1}&=\frac{\beta_f c_{f11}\left(\sum_{h\in\{u,v\}}I_{f1}^{h}+L_{f1}^{h}+H_{f1}^{h}+C_{f1}^{h}\right)+\beta_f c_{f21}\left(\sum_{h\in\{u,v\}}I_{f2}^{h}+L_{f2}^{h}+H_{f2}^{h}+C_{f2}^{h}\right)}{N_{m1}},\\
\lambda_{f2}&=\frac{\beta_f c_{f12}\left(\sum_{h\in\{u,v\}}I_{f1}^{h}+L_{f1}^{h}+H_{f1}^{h}+C_{f1}^{h}\right)+\beta_f c_{f22}\left(\sum_{h\in\{u,v\}}I_{f2}^{h}+L_{f2}^{h}+H_{f2}^{h}+C_{f2}^{h}\right)}{N_{m2}},\\
N_{f1}&=S_{f1}^{u}+I_{f1}^{u}+L_{f1}^{u}+H_{f1}^{u}+C_{f1}^{u}+S_{f1}^{v}+I_{f1}^{v}+L_{f1}^{v}+H_{f1}^{v}+C_{f1}^{v}+D_{f1}+R_{f1},\\
N_{f2}&=S_{f2}^{u}+I_{f2}^{u}+L_{f2}^{u}+H_{f2}^{u}+C_{f2}^{u}+S_{f2}^{v}+I_{f2}^{v}+L_{f2}^{v}+H_{f2}^{v}+C_{f2}^{v}+D_{f2}+R_{f2},\\
N_{m1}&=S_{m1}^{u}+I_{m1}^{u}+S_{m1}^{v}+I_{m1}^{v}+R_{m1},\\
N_{m2}&=S_{m2}^{u}+I_{m2}^{u}+S_{m2}^{v}+I_{m2}^{v}+R_{m2},\\
N_f&=N_{f1}+N_{f2},\\
N_m&=N_{m1}+N_{m2}.
\end{aligned}
$$

The initial conditions for system (S8.1)-(S8.4) is as follows:

$$
x_i(0)\in[0,\hat{M}],\, i=1,2,\cdots,34, \tag{S8.5}
$$

where $\hat{M}$ is a positive constant, and x_i is the i-th element in x. Let $F_c(t,x^T,\mathbf{u}^T)=(f_1,f_2,\cdots,f_{34})^T$, where f_i is the i-th right-hand side function of (S8.1)-(S8.4).

Appendix 9. Proof of Theorem 5.1

Proof. (1) It is easy to see that $F_c(t, x^T, \mathbf{u}^T)$ is C^1. And, from Theorem 3.1, x_i is bounded, for $i = 1, 2, \cdots, 34$. Thus, it is easy to see that $F_c(t, 0, 0)$ and the partial derivatives of $F_c(t, x^T, \mathbf{u}^T)$ with respect to all control variables and state variables are bounded. (2) From Theorem 3.1, system (S8.1)-(S8.5) with a constant control has a unique solution. Therefore, the set

$$\begin{aligned}\mathscr{F} = \{&(\mathbf{u}, x(0))|\text{There exists a solution to system (S8.1)-(S8.5)}\\ &\text{with control } \mathbf{u} \in U \text{and initial condition } x(0)\}\end{aligned}$$

is non-empty. (3) Obviously, U is convex and closed. And, $F(t, x^T, \mathbf{u}^T)$ is linear with respect to $\mathbf{u}$, and the coefficients depend on time t and state x. (4) Since the initial value $x_i(0)$ of each state variable is bounded, according to the similar proof of Theorem 3.1, it can be seen that there is a compact and invariant set containing $x(t)$ for every $t \geq 0$. Further, there exists a compact set $\mathcal{S}$ such that all $e \in \mathcal{S}$ and $\phi(e)$ is continuous on $\mathcal{S}$. (5) For any $\mathbf{u}_1 \in U$, $\mathbf{u}_2 \in U$ and $q \in [0, 1]$,

$$(1-q)\Phi(t, x, \mathbf{u}_1) + q\Phi(t, x, \mathbf{u}_2) \geq \Phi(t, x, (1-q)\mathbf{u}_1 + q\mathbf{u}_2),$$

because

$$(1-q)u_{1i}^2 + qu_{2i}^2 \geq [(1-q)u_{1i} + qu_{2i}]^2$$

where u_{1i} and u_{2i} are the elements of $\mathbf{u}_1$ and $\mathbf{u}_2$, respectively. Hence, $\Phi(t, x, \mathbf{u})$ is convex on U. (6) There are constants $C_1 > 0$, C_2 and $b > 1$, such that

$$\Phi(t, x, \mathbf{u}) \geq C_1|\mathbf{u}|^b - C_2,$$

where $|\mathbf{u}|$ is magnitude of $\mathbf{u}$. Obviously, $\Phi(t, x, \mathbf{u}) \geq 0 \geq |\mathbf{u}|^2 - \max|\mathbf{u}|^2$, hence we take $C_1 = 1$, $b = 2$ and $C_2 = \max|\mathbf{u}|^2 = 3.7524$. To sum up, from Theorem 4.1 and Corollary 4.1 in [23], Theorem 5.1 is proved.

Appendix 10. The adjoint equations (S10.1)-(S10.35)

$$\begin{aligned}p_1' =& -\frac{\partial\mathscr{H}(t, \mathbf{u}^*, x^*, p)}{\partial S_{f1}^u}\\ =& -a_2\kappa_1(B_2k_f^*(t))^2S_{f1}^{u*}(t) - a_2\kappa_2(E_2\sigma_{f1}^{u*}(t))^2(S_{f1}^{u*}(t) + I_{f1}^{u*}(t) + R_{f1}^*(t))\\ &+ p_1\left(k_f^*(t) + \mu_{f1} + \alpha_f\right) - (p_2 - p_1)\left.\frac{\partial(\lambda_{m1}S_{f1}^u)}{\partial S_{f1}^u}\right|_{(\mathbf{u}^*, x^*)}\\ &- p_6k_f^*(t) - (p_7 - p_6)(1 - \varepsilon_{f1})\left.\frac{\partial(\lambda_{m1}S_{f1}^v)}{\partial S_{f1}^u}\right|_{(\mathbf{u}^*, x^*)} - p_{13}\alpha_f, \qquad \text{(S10.1)}\\ p_2' =& -\frac{\partial\mathscr{H}(t, \mathbf{u}^*, x^*, p)}{\partial I_{f1}^u}\\ =& -a_2\kappa_2(E_2\sigma_{f1}^{u*}(t))^2(S_{f1}^{u*}(t) + I_{f1}^{u*}(t) + R_{f1}^*(t))\\ &- (p_2 - p_1)\left.\frac{\partial(\lambda_{m1}S_{f1}^u)}{\partial I_{f1}^u}\right|_{(\mathbf{u}^*, x^*)} + p_2\left(\rho + \mu_{f1} + \alpha_f\right) - p_3(1 - r_{f1}^u)\rho\end{aligned}$$

$$
\begin{aligned}
&- (p_7 - p_6)(1-\varepsilon_{f1}) \left.\frac{\partial(\lambda_{m1}S^v_{f1})}{\partial I^u_{f1}}\right|_{(\mathbf{u}^*,x^*)} - p_{12} r^u_{f1}\rho - p_{14}\alpha_f \\
&- (p_{26} - p_{25}) \left.\frac{\partial(\lambda_{f1}S^u_{m1})}{\partial I^u_{f1}}\right|_{(\mathbf{u}^*,x^*)} - (p_{28}-p_{27})(1-\varepsilon_{m1}) \left.\frac{\partial(\lambda_{f1}S^v_{m1})}{\partial I^u_{f1}}\right|_{(\mathbf{u}^*,x^*)} \\
&- (p_{31} - p_{30}) \left.\frac{\partial(\lambda_{f2}S^u_{m2})}{\partial I^u_{f1}}\right|_{(\mathbf{u}^*,x^*)} - (p_{33}-p_{32})(1-\varepsilon_{m2}) \left.\frac{\partial(\lambda_{f2}S^v_{m2})}{\partial I^u_{f1}}\right|_{(\mathbf{u}^*,x^*)}, \qquad \text{(S10.2)}
\end{aligned}
$$

$$
\begin{aligned}
p_3^{'} =& - \frac{\partial \mathscr{H}(t,\mathbf{u}^*,x^*,p)}{\partial L^u_{f1}} \\
=& - a_2\kappa_2 (E_1\sigma^{u*}_{f1}(t))^2 L^{u*}_{f1}(t) - a_2\kappa_3 \left(T_L \sigma^{u*}_{f1}(t)\right)^2 L^{u*}_{f1}(t) \\
&- (p_2 - p_1) \left.\frac{\partial(\lambda_{m1}S^u_{f1})}{\partial L^u_{f1}}\right|_{(\mathbf{u}^*,x^*)} + p_3(\eta + \sigma^{u*}_{f1}(t) + \mu_{f1} + \alpha_f) - p_4(1-e^u_{f1})\eta \\
&- (p_7 - p_6)(1-\varepsilon_{f1}) \left.\frac{\partial(\lambda_{m1}S^v_{f1})}{\partial L^u_{f1}}\right|_{(\mathbf{u}^*,x^*)} - p_{11}\sigma^{u*}_{f1}(t) - p_{12} e^u_{f1}\eta - p_{15}\alpha_f \\
&- (p_{26} - p_{25}) \left.\frac{\partial(\lambda_{f1}S^u_{m1})}{\partial L^u_{f1}}\right|_{(\mathbf{u}^*,x^*)} - (p_{28}-p_{27})(1-\varepsilon_{m1}) \left.\frac{\partial(\lambda_{f1}S^v_{m1})}{\partial L^u_{f1}}\right|_{(\mathbf{u}^*,x^*)} \\
&- (p_{31} - p_{30}) \left.\frac{\partial(\lambda_{f2}S^u_{m2})}{\partial L^u_{f1}}\right|_{(\mathbf{u}^*,x^*)} - (p_{33}-p_{32})(1-\varepsilon_{m2}) \left.\frac{\partial(\lambda_{f2}S^v_{m2})}{\partial L^u_{f1}}\right|_{(\mathbf{u}^*,x^*)}, \qquad \text{(S10.3)}
\end{aligned}
$$

$$
\begin{aligned}
p_4^{'} =& - \frac{\partial \mathscr{H}(t,\mathbf{u}^*,x^*,p)}{\partial H^u_{f1}} \\
=& - a_2\kappa_2 (E_1\sigma^{u*}_{f1}(t))^2 H^{u*}_{f1}(t) - a_2\kappa_3 \left(T_H \sigma^{u*}_{f1}(t)\right)^2 H^{u*}_{f1}(t) \\
&- (p_2 - p_1) \left.\frac{\partial(\lambda_{m1}S^u_{f1})}{\partial H^u_{f1}}\right|_{(\mathbf{u}^*,x^*)} + p_4(\xi^u_{f1} + \sigma^{u*}_{f1}(t) + \mu_{f1} + \alpha_f) - p_5\xi^u_{f1} \\
&- (p_7 - p_6)(1-\varepsilon_{f1}) \left.\frac{\partial(\lambda_{m1}S^v_{f1})}{\partial H^u_{f1}}\right|_{(\mathbf{u}^*,x^*)} - p_{11}\sigma^{u*}_{f1}(t) - p_{16}\alpha_f \\
&- (p_{26} - p_{25}) \left.\frac{\partial(\lambda_{f1}S^u_{m1})}{\partial H^u_{f1}}\right|_{(\mathbf{u}^*,x^*)} - (p_{28}-p_{27})(1-\varepsilon_{m1}) \left.\frac{\partial(\lambda_{f1}S^v_{m1})}{\partial H^u_{f1}}\right|_{(\mathbf{u}^*,x^*)} \\
&- (p_{31} - p_{30}) \left.\frac{\partial(\lambda_{f2}S^u_{m2})}{\partial H^u_{f1}}\right|_{(\mathbf{u}^*,x^*)} - (p_{33}-p_{32})(1-\varepsilon_{m2}) \left.\frac{\partial(\lambda_{f2}S^v_{m2})}{\partial H^u_{f1}}\right|_{(\mathbf{u}^*,x^*)}, \qquad \text{(S10.4)}
\end{aligned}
$$

$$
\begin{aligned}
p_5^{'} =& - \frac{\partial \mathscr{H}(t,\mathbf{u}^*,x^*,p)}{\partial C^u_{f1}} \\
=& - a_2\kappa_2 \left\{E_1[\Lambda + (1-\Lambda)\sigma^{u*}_{f1}(t)]\right\}^2 C^{u*}_{f1}(t) - a_2\kappa_3 \left\{T_C[\Lambda + (1-\Lambda)\sigma^{u*}_{f1}(t)]\right\}^2 C^{u*}_{f1}(t) \\
&- (p_2 - p_1) \left.\frac{\partial(\lambda_{m1}S^u_{f1})}{\partial C^u_{f1}}\right|_{(\mathbf{u}^*,x^*)} + p_5[\Lambda + (1-\Lambda)\sigma^{u*}_{f1}(t) + \mu_{f1} + \alpha_f] \\
&- (p_7 - p_6)(1-\varepsilon_{f1}) \left.\frac{\partial(\lambda_{m1}S^v_{f1})}{\partial C^u_{f1}}\right|_{(\mathbf{u}^*,x^*)} - p_{11}[\Lambda + (1-\Lambda)\sigma^{u*}_{f1}(t)] - p_{17}\alpha_f \\
&- (p_{26} - p_{25}) \left.\frac{\partial(\lambda_{f1}S^u_{m1})}{\partial C^u_{f1}}\right|_{(\mathbf{u}^*,x^*)} - (p_{28}-p_{27})(1-\varepsilon_{m1}) \left.\frac{\partial(\lambda_{f1}S^v_{m1})}{\partial C^u_{f1}}\right|_{(\mathbf{u}^*,x^*)}
\end{aligned}
$$

$$
\begin{aligned}
&- (p_{31} - p_{30}) \left.\frac{\partial(\lambda_{f2} S^u_{m2})}{\partial C^u_{f1}}\right|_{(\mathbf{u}^*,x^*)} - (p_{33} - p_{32})(1-\varepsilon_{m2}) \left.\frac{\partial(\lambda_{f2} S^v_{m2})}{\partial C^u_{f1}}\right|_{(\mathbf{u}^*,x^*)}, \qquad \text{(S10.5)}\\
p_6^{'} =& - \frac{\partial \mathscr{H}(t,\mathbf{u}^*,x^*,p)}{\partial S^v_{f1}}\\
=& - a_2\kappa_2(E_2\sigma^{v*}_{f1}(t))^2(S^{v*}_{f1}(t) + I^{v*}_{f1}(t)) - (p_2 - p_1) \left.\frac{\partial(\lambda_{m1} S^u_{f1})}{\partial S^v_{f1}}\right|_{(\mathbf{u}^*,x^*)} - p_1\delta\\
&+ p_6(\delta + \mu_{f1} + \alpha_f) - (p_7 - p_6)(1-\varepsilon_{f1}) \left.\frac{\partial(\lambda_{m1} S^v_{f1})}{\partial S^v_{f1}}\right|_{(\mathbf{u}^*,x^*)} - p_{18}\alpha_f, \qquad \text{(S10.6)}\\
p_7^{'} =& - \frac{\partial \mathscr{H}(t,\mathbf{u}^*,x^*,p)}{\partial I^v_{f1}}\\
=& - a_2\kappa_2(E_2\sigma^{v*}_{f1}(t))^2(S^{v*}_{f1}(t) + I^{v*}_{f1}(t))\\
&- (p_2 - p_1) \left.\frac{\partial(\lambda_{m1} S^u_{f1})}{\partial I^v_{f1}}\right|_{(\mathbf{u}^*,x^*)} - (p_7 - p_6)(1-\varepsilon_{f1}) \left.\frac{\partial(\lambda_{m1} S^v_{f1})}{\partial I^v_{f1}}\right|_{(\mathbf{u}^*,x^*)}\\
&+ p_7(\rho + \mu_{f1} + \alpha_f) - p_8(1 - r^v_{f1})\rho - p_{12} r^v_{f1}\rho - p_{19}\alpha_f\\
&- (p_{26} - p_{25}) \left.\frac{\partial(\lambda_{f1} S^u_{m1})}{\partial I^v_{f1}}\right|_{(\mathbf{u}^*,x^*)} - (p_{28} - p_{27})(1-\varepsilon_{m1}) \left.\frac{\partial(\lambda_{f1} S^v_{m1})}{\partial I^v_{f1}}\right|_{(\mathbf{u}^*,x^*)}\\
&- (p_{31} - p_{30}) \left.\frac{\partial(\lambda_{f2} S^u_{m2})}{\partial I^v_{f1}}\right|_{(\mathbf{u}^*,x^*)} - (p_{33} - p_{32})(1-\varepsilon_{m2}) \left.\frac{\partial(\lambda_{f2} S^v_{m2})}{\partial I^v_{f1}}\right|_{(\mathbf{u}^*,x^*)}, \qquad \text{(S10.7)}\\
p_8^{'} =& - \frac{\partial \mathscr{H}(t,\mathbf{u}^*,x^*,p)}{\partial L^v_{f1}}\\
=& - a_2\kappa_2(E_1\sigma^{v*}_{f1}(t))^2 L^{v*}_{f1}(t) - a_2\kappa_3 \left(T_L\sigma^{v*}_{f1}(t)\right)^2 L^{v*}_{f1}(t)\\
&- (p_2 - p_1) \left.\frac{\partial(\lambda_{m1} S^u_{f1})}{\partial L^v_{f1}}\right|_{(\mathbf{u}^*,x^*)} - (p_7 - p_6)(1-\varepsilon_{f1}) \left.\frac{\partial(\lambda_{m1} S^v_{f1})}{\partial L^v_{f1}}\right|_{(\mathbf{u}^*,x^*)}\\
&+ p_8(\eta + \sigma^{v*}_{f1}(t) + \mu_{f1} + \alpha_f) - p_9(1 - e^v_{f1})\eta - p_{11}\sigma^{v*}_{f1}(t) - p_{12} e^v_{f1}\eta - p_{20}\alpha_f\\
&- (p_{26} - p_{25}) \left.\frac{\partial(\lambda_{f1} S^u_{m1})}{\partial L^v_{f1}}\right|_{(\mathbf{u}^*,x^*)} - (p_{28} - p_{27})(1-\varepsilon_{m1}) \left.\frac{\partial(\lambda_{f1} S^v_{m1})}{\partial L^v_{f1}}\right|_{(\mathbf{u}^*,x^*)}\\
&- (p_{31} - p_{30}) \left.\frac{\partial(\lambda_{f2} S^u_{m2})}{\partial L^v_{f1}}\right|_{(\mathbf{u}^*,x^*)} - (p_{33} - p_{32})(1-\varepsilon_{m2}) \left.\frac{\partial(\lambda_{f2} S^v_{m2})}{\partial L^v_{f1}}\right|_{(\mathbf{u}^*,x^*)}, \qquad \text{(S10.8)}\\
p_9^{'} =& - \frac{\partial \mathscr{H}(t,\mathbf{u}^*,x^*,p)}{\partial H^v_{f1}}\\
=& - a_2\kappa_2(E_1\sigma^{v*}_{f1}(t))^2 H^{v*}_{f1}(t) - a_2\kappa_3 \left(T_H\sigma^{v*}_{f1}(t)\right)^2 H^{v*}_{f1}(t)\\
&- (p_2 - p_1) \left.\frac{\partial(\lambda_{m1} S^u_{f1})}{\partial H^v_{f1}}\right|_{(\mathbf{u}^*,x^*)} - (p_7 - p_6)(1-\varepsilon_{f1}) \left.\frac{\partial(\lambda_{m1} S^v_{f1})}{\partial H^v_{f1}}\right|_{(\mathbf{u}^*,x^*)}\\
&+ p_9(\xi^v_{f1} + \sigma^{v*}_{f1}(t) + \mu_{f1} + \alpha_f) - p_{10}\xi^v_{f1} - p_{11}\sigma^{v*}_{f1}(t) - p_{21}\alpha_f\\
&- (p_{26} - p_{25}) \left.\frac{\partial(\lambda_{f1} S^u_{m1})}{\partial H^v_{f1}}\right|_{(\mathbf{u}^*,x^*)} - (p_{28} - p_{27})(1-\varepsilon_{m1}) \left.\frac{\partial(\lambda_{f1} S^v_{m1})}{\partial H^v_{f1}}\right|_{(\mathbf{u}^*,x^*)}\\
&- (p_{31} - p_{30}) \left.\frac{\partial(\lambda_{f2} S^u_{m2})}{\partial H^v_{f1}}\right|_{(\mathbf{u}^*,x^*)} - (p_{33} - p_{32})(1-\varepsilon_{m2}) \left.\frac{\partial(\lambda_{f2} S^v_{m2})}{\partial H^v_{f1}}\right|_{(\mathbf{u}^*,x^*)}, \qquad \text{(S10.9)}
\end{aligned}
$$

$$
\begin{aligned}
p_{10}^{'} =& -\frac{\partial \mathscr{H}(t,\mathbf{u}^{*},x^{*},p)}{\partial C_{f1}^{v}} \\
=& -a_2\kappa_2\left\{E_1[\Lambda+(1-\Lambda)\sigma_{f1}^{v*}(t)]\right\}^2 C_{f1}^{v*}(t) - a_2\kappa_3\left\{T_C[\Lambda+(1-\Lambda)\sigma_{f1}^{v*}(t)]\right\}^2 C_{f1}^{v*}(t) \\
& -(p_2-p_1)\left.\frac{\partial(\lambda_{m1}S_{f1}^{u})}{\partial C_{f1}^{v}}\right|_{(\mathbf{u}^*,x^*)} -(p_7-p_6)(1-\varepsilon_{f1})\left.\frac{\partial(\lambda_{m1}S_{f1}^{v*})}{\partial C_{f1}^{v*}}\right|_{(\mathbf{u}^*,x^*)} \\
& +p_{10}[\Lambda+(1-\Lambda)\sigma_{f1}^{v*}(t)+\mu_{f1}+\alpha_f] - p_{11}[\Lambda+(1-\Lambda)\sigma_{f1}^{v*}(t)] - p_{22}\alpha_f \\
& -(p_{26}-p_{25})\left.\frac{\partial(\lambda_{f1}S_{m1}^{u})}{\partial C_{f1}^{v}}\right|_{(\mathbf{u}^*,x^*)} -(p_{28}-p_{27})(1-\varepsilon_{m1})\left.\frac{\partial(\lambda_{f1}S_{m1}^{v})}{\partial C_{f1}^{v}}\right|_{(\mathbf{u}^*,x^*)} \\
& -(p_{31}-p_{30})\left.\frac{\partial(\lambda_{f2}S_{m2}^{u})}{\partial C_{f1}^{v}}\right|_{(\mathbf{u}^*,x^*)} -(p_{33}-p_{32})(1-\varepsilon_{m2})\left.\frac{\partial(\lambda_{f2}S_{m2}^{v})}{\partial C_{f1}^{v}}\right|_{(\mathbf{u}^*,x^*)}, \quad \text{(S10.10)} \\
p_{11}^{'} =& -\frac{\partial \mathscr{H}(t,\mathbf{u}^{*},x^{*},p)}{\partial D_{f1}} \\
=& -(p_2-p_1)\left.\frac{\partial(\lambda_{m1}S_{f1}^{u})}{\partial D_{f1}}\right|_{(\mathbf{u}^*,x^*)} -(p_7-p_6)(1-\varepsilon_{f1})\left.\frac{\partial(\lambda_{m1}S_{f1}^{v})}{\partial D_{f1}}\right|_{(\mathbf{u}^*,x^*)} \\
& +p_{11}(\gamma_{f1}+d_{f1}+\mu_{f1}+\alpha_f) - p_{12}\gamma_{f1} - p_{23}\alpha_f, \quad \text{(S10.11)} \\
p_{12}^{'} =& -\frac{\partial \mathscr{H}(t,\mathbf{u}^{*},x^{*},p)}{\partial R_{f1}} \\
=& -a_2\kappa_2(E_2\sigma_{f1}^{u*}(t))^2(S_{f1}^{u*}(t)+I_{f1}^{u*}(t)+R_{f1}^{*}(t)) \\
& -p_1\zeta_{f1}-(p_2-p_1)\left.\frac{\partial(\lambda_{m1}S_{f1}^{u})}{\partial R_{f1}}\right|_{(\mathbf{u}^*,x^*)} -(p_7-p_6)(1-\varepsilon_{f1})\left.\frac{\partial(\lambda_{m1}S_{f1}^{v})}{\partial R_{f1}}\right|_{(\mathbf{u}^*,x^*)} \\
& +p_{12}(\mu_{f1}+\alpha_f+\zeta_{f1}) - p_{24}\alpha_f, \quad \text{(S10.12)} \\
p_{13}^{'} =& -\frac{\partial \mathscr{H}(t,\mathbf{u}^{*},x^{*},p)}{\partial S_{f2}^{u}} \\
=& -a_2\kappa_2(E_2\sigma_{f2}^{u*}(t))^2(S_{f2}^{u*}(t)+I_{f2}^{u*}(t)+R_{f2}^{*}(t)) + p_{13}\mu_{f2} \\
& -(p_{14}-p_{13})\left.\frac{\partial(\lambda_{m2}S_{f2}^{u})}{\partial S_{f2}^{u}}\right|_{(\mathbf{u}^*,x^*)} -(p_{19}-p_{18})(1-\varepsilon_{f2})\left.\frac{\partial(\lambda_{m2}S_{f2}^{v})}{\partial S_{f2}^{u}}\right|_{(\mathbf{u}^*,x^*)}, \quad \text{(S10.13)} \\
p_{14}^{'} =& -\frac{\partial \mathscr{H}(t,\mathbf{u}^{*},x^{*},p)}{\partial I_{f2}^{u}} \\
=& -a_2\kappa_2(E_2\sigma_{f2}^{u*}(t))^2(S_{f2}^{u*}(t)+I_{f2}^{u*}(t)+R_{f2}^{*}(t)) \\
& -(p_{14}-p_{13})\left.\frac{\partial(\lambda_{m2}S_{f2}^{u})}{\partial I_{f2}^{u}}\right|_{(\mathbf{u}^*,x^*)} + p_{14}(\rho+\mu_{f2}) - p_{15}(1-r_{f2}^{u})\rho \\
& -(p_{19}-p_{18})(1-\varepsilon_{f2})\left.\frac{\partial(\lambda_{m2}S_{f2}^{v})}{\partial I_{f2}^{u}}\right|_{(\mathbf{u}^*,x^*)} - p_{24}r_{f2}^{u}\rho \\
& -(p_{26}-p_{25})\left.\frac{\partial(\lambda_{f1}S_{m1}^{u})}{\partial I_{f2}^{u}}\right|_{(\mathbf{u}^*,x^*)} -(p_{28}-p_{27})(1-\varepsilon_{m1})\left.\frac{\partial(\lambda_{f1}S_{m1}^{v})}{\partial I_{f2}^{u}}\right|_{(\mathbf{u}^*,x^*)} \\
& -(p_{31}-p_{30})\left.\frac{\partial(\lambda_{f2}S_{m2}^{u})}{\partial I_{f2}^{u}}\right|_{(\mathbf{u}^*,x^*)} -(p_{33}-p_{32})(1-\varepsilon_{m2})\left.\frac{\partial(\lambda_{f2}S_{m2}^{v})}{\partial I_{f2}^{u}}\right|_{(\mathbf{u}^*,x^*)}, \quad \text{(S10.14)} \\
p_{15}^{'} =& -\frac{\partial \mathscr{H}(t,\mathbf{u}^{*},x^{*},p)}{\partial L_{f2}^{u}}
\end{aligned}
$$

$$
\begin{aligned}
=&- a_2\kappa_2(E_1\sigma_{f2}^{u*}(t))^2L_{f2}^{u*}(t) - a_2\kappa_3\left(T_L\sigma_{f2}^{u*}(t)\right)^2L_{f2}^{u*}(t)\\
&-(p_{14}-p_{13})\left.\frac{\partial(\lambda_{m2}S_{f2}^u)}{\partial L_{f2}^u}\right|_{(\mathbf{u}^*,x^*)} + p_{15}(\eta+\sigma_{f2}^{u*}(t)+\mu_{f2}) - p_{16}(1-e_{f2}^u)\eta\\
&-(p_{19}-p_{18})(1-\varepsilon_{f2})\left.\frac{\partial(\lambda_{m2}S_{f2}^v)}{\partial L_{f2}^u}\right|_{(\mathbf{u}^*,x^*)} - p_{23}\sigma_{f2}^{u*}(t) - p_{24}e_{f2}^u\eta\\
&-(p_{26}-p_{25})\left.\frac{\partial(\lambda_{f1}S_{m1}^u)}{\partial L_{f2}^u}\right|_{(\mathbf{u}^*,x^*)} - (p_{28}-p_{27})(1-\varepsilon_{m1})\left.\frac{\partial(\lambda_{f1}S_{m1}^v)}{\partial L_{f2}^u}\right|_{(\mathbf{u}^*,x^*)}\\
&-(p_{31}-p_{30})\left.\frac{\partial(\lambda_{f2}S_{m2}^u)}{\partial L_{f2}^u}\right|_{(\mathbf{u}^*,x^*)} - (p_{33}-p_{32})(1-\varepsilon_{m2})\left.\frac{\partial(\lambda_{f2}S_{m2}^v)}{\partial L_{f2}^u}\right|_{(\mathbf{u}^*,x^*)}, \qquad \text{(S10.15)}
\end{aligned}
$$

$$
\begin{aligned}
p_{16}^{'} =&-\frac{\partial\mathscr{H}(t,\mathbf{u}^*,x^*,p)}{\partial H_{f2}^u}\\
=&- a_2\kappa_2(E_1\sigma_{f2}^{u*}(t))^2H_{f2}^{u*}(t) - a_2\kappa_3\left(T_H\sigma_{f2}^{u*}(t)\right)^2H_{f2}^{u*}(t)\\
&-(p_{14}-p_{13})\left.\frac{\partial(\lambda_{m2}S_{f2}^u)}{\partial H_{f2}^u}\right|_{(\mathbf{u}^*,x^*)} + p_{16}(\xi_{f2}^u+\sigma_{f2}^{u*}(t)+\mu_{f2}) - p_{17}\xi_{f2}^u\\
&-(p_{19}-p_{18})(1-\varepsilon_{f2})\left.\frac{\partial(\lambda_{m2}S_{f2}^v)}{\partial H_{f2}^u}\right|_{(\mathbf{u}^*,x^*)} - p_{23}\sigma_{f2}^{u*}(t)\\
&-(p_{26}-p_{25})\left.\frac{\partial(\lambda_{f1}S_{m1}^u)}{\partial H_{f2}^u}\right|_{(\mathbf{u}^*,x^*)} - (p_{28}-p_{27})(1-\varepsilon_{m1})\left.\frac{\partial(\lambda_{f1}S_{m1}^v)}{\partial H_{f2}^u}\right|_{(\mathbf{u}^*,x^*)}\\
&-(p_{31}-p_{30})\left.\frac{\partial(\lambda_{f2}S_{m2}^u)}{\partial H_{f2}^u}\right|_{(\mathbf{u}^*,x^*)} - (p_{33}-p_{32})(1-\varepsilon_{m2})\left.\frac{\partial(\lambda_{f2}S_{m2}^v)}{\partial H_{f2}^u}\right|_{(\mathbf{u}^*,x^*)}, \qquad \text{(S10.16)}
\end{aligned}
$$

$$
\begin{aligned}
p_{17}^{'} =&-\frac{\partial\mathscr{H}(t,\mathbf{u}^*,x^*,p)}{\partial C_{f2}^u}\\
=&- a_2\kappa_2\left\{E_1[\Lambda+(1-\Lambda)\sigma_{f2}^{u*}(t)]\right\}^2C_{f2}^{u*}(t) - a_2\kappa_3\left\{T_C[\Lambda+(1-\Lambda)\sigma_{f2}^{u*}(t)]\right\}^2C_{f2}^{u*}(t)\\
&-(p_{14}-p_{13})\left.\frac{\partial(\lambda_{m2}S_{f2}^u)}{\partial C_{f2}^u}\right|_{(\mathbf{u}^*,x^*)} + p_{17}[\Lambda+(1-\Lambda)\sigma_{f2}^{u*}(t)+\mu_{f2}]\\
&-(p_{19}-p_{18})(1-\varepsilon_{f2})\left.\frac{\partial(\lambda_{m2}S_{f2}^v)}{\partial C_{f2}^u}\right|_{(\mathbf{u}^*,x^*)} - p_{23}[\Lambda+(1-\Lambda)\sigma_{f2}^{u*}(t)]\\
&-(p_{26}-p_{25})\left.\frac{\partial(\lambda_{f1}S_{m1}^u)}{\partial C_{f2}^u}\right|_{(\mathbf{u}^*,x^*)} - (p_{28}-p_{27})(1-\varepsilon_{m1})\left.\frac{\partial(\lambda_{f1}S_{m1}^v)}{\partial C_{f2}^u}\right|_{(\mathbf{u}^*,x^*)}\\
&-(p_{31}-p_{30})\left.\frac{\partial(\lambda_{f2}S_{m2}^u)}{\partial C_{f2}^u}\right|_{(\mathbf{u}^*,x^*)} - (p_{33}-p_{32})(1-\varepsilon_{m2})\left.\frac{\partial(\lambda_{f2}S_{m2}^v)}{\partial C_{f2}^u}\right|_{(\mathbf{u}^*,x^*)}, \qquad \text{(S10.17)}
\end{aligned}
$$

$$
\begin{aligned}
p_{18}^{'} =&-\frac{\partial\mathscr{H}(t,\mathbf{u}^*,x^*,p)}{\partial S_{f2}^v}\\
=&- a_2\kappa_2(E_2\sigma_{f2}^{v*}(t))^2(S_{f2}^{v*}(t)+I_{f2}^{v*}(t)) - p_{13}\delta - (p_{14}-p_{13})\left.\frac{\partial(\lambda_{m2}S_{f2}^u)}{\partial S_{f2}^v}\right|_{(\mathbf{u}^*,x^*)}\\
&-(p_{19}-p_{18})(1-\varepsilon_{f2})\left.\frac{\partial(\lambda_{m2}S_{f2}^v)}{\partial S_{f2}^v}\right|_{(\mathbf{u}^*,x^*)} + p_{18}(\delta+\mu_{f2}), \qquad \text{(S10.18)}
\end{aligned}
$$

$$
\begin{aligned}
p_{19}^{'} =& -\frac{\partial \mathscr{H}(t,\mathbf{u}^*,x^*,p)}{\partial I_{f2}^v} \\
=& -a_2\kappa_2(E_2\sigma_{f2}^{v*}(t))^2(S_{f2}^{v*}(t)+I_{f2}^{v*}(t)) \\
& -(p_{14}-p_{13})\left.\frac{\partial(\lambda_{m2}S_{f2}^u)}{\partial I_{f2}^v}\right|_{(\mathbf{u}^*,x^*)} -(p_{19}-p_{18})(1-\varepsilon_{f2})\left.\frac{\partial(\lambda_{m2}S_{f2}^v)}{\partial I_{f2}^v}\right|_{(\mathbf{u}^*,x^*)} \\
& +p_{19}(\rho+\mu_{f2})-p_{20}(1-r_{f2}^v)\rho-p_{24}r_{f2}^v\rho-(p_{26}-p_{25})\left.\frac{\partial(\lambda_{f1}S_{m1}^u)}{\partial I_{f2}^v}\right|_{(\mathbf{u}^*,x^*)} \\
& -(p_{28}-p_{27})(1-\varepsilon_{m1})\left.\frac{\partial(\lambda_{f1}S_{m1}^v)}{\partial I_{f2}^v}\right|_{(\mathbf{u}^*,x^*)} -(p_{31}-p_{30})\left.\frac{\partial(\lambda_{f2}S_{m2}^u)}{\partial I_{f2}^v}\right|_{(\mathbf{u}^*,x^*)} \\
& -(p_{33}-p_{32})(1-\varepsilon_{m2})\left.\frac{\partial(\lambda_{f2}S_{m2}^v)}{\partial I_{f2}^v}\right|_{(\mathbf{u}^*,x^*)}, \qquad \text{(S10.19)}
\end{aligned}
$$

$$
\begin{aligned}
p_{20}^{'} =& -\frac{\partial \mathscr{H}(t,\mathbf{u}^*,x^*,p)}{\partial L_{f2}^v} \\
=& -a_2\kappa_2(E_1\sigma_{f2}^{v*}(t))^2L_{f2}^{v*}(t)-a_2\kappa_3\left(T_L\sigma_{f2}^{v*}(t)\right)^2L_{f2}^{v*}(t) \\
& -(p_{14}-p_{13})\left.\frac{\partial(\lambda_{m2}S_{f2}^u)}{\partial L_{f2}^v}\right|_{(\mathbf{u}^*,x^*)} -(p_{19}-p_{18})(1-\varepsilon_{f2})\left.\frac{\partial(\lambda_{m2}S_{f2}^v)}{\partial L_{f2}^v}\right|_{(\mathbf{u}^*,x^*)} \\
& +p_{20}(\eta+\sigma_{f2}^{v*}(t)+\mu_{f2})-p_{21}(1-e_{f2}^v)\eta-p_{23}\sigma_{f2}^{v*}(t)-p_{24}e_{f2}^v\eta \\
& -(p_{26}-p_{25})\left.\frac{\partial(\lambda_{f1}S_{m1}^u)}{\partial L_{f2}^v}\right|_{(\mathbf{u}^*,x^*)} -(p_{28}-p_{27})(1-\varepsilon_{m1})\left.\frac{\partial(\lambda_{f1}S_{m1}^v)}{\partial L_{f2}^v}\right|_{(\mathbf{u}^*,x^*)} \\
& -(p_{31}-p_{30})\left.\frac{\partial(\lambda_{f2}S_{m2}^u)}{\partial L_{f2}^v}\right|_{(\mathbf{u}^*,x^*)} -(p_{33}-p_{32})(1-\varepsilon_{m2})\left.\frac{\partial(\lambda_{f2}S_{m2}^v)}{\partial L_{f2}^v}\right|_{(\mathbf{u}^*,x^*)}, \qquad \text{(S10.20)}
\end{aligned}
$$

$$
\begin{aligned}
p_{21}^{'} =& -\frac{\partial \mathscr{H}(t,\mathbf{u}^*,x^*,p)}{\partial H_{f2}^v} \\
=& -a_2\kappa_2(E_1\sigma_{f2}^{v*}(t))^2H_{f2}^{v*}(t)-a_2\kappa_3\left(T_H\sigma_{f2}^{v*}(t)\right)^2H_{f2}^{v*}(t) \\
& -(p_{14}-p_{13})\left.\frac{\partial(\lambda_{m2}S_{f2}^u)}{\partial H_{f2}^v}\right|_{(\mathbf{u}^*,x^*)} -(p_{19}-p_{18})(1-\varepsilon_{f2})\left.\frac{\partial(\lambda_{m2}S_{f2}^v)}{\partial H_{f2}^v}\right|_{(\mathbf{u}^*,x^*)} \\
& +p_{21}(\xi_{f2}^v+\sigma_{f2}^{v*}(t)+\mu_{f2})-p_{22}\xi_{f2}^v-p_{23}\sigma_{f2}^{v*}(t) \\
& -(p_{26}-p_{25})\left.\frac{\partial(\lambda_{f1}S_{m1}^u)}{\partial H_{f2}^v}\right|_{(\mathbf{u}^*,x^*)} -(p_{28}-p_{27})(1-\varepsilon_{m1})\left.\frac{\partial(\lambda_{f1}S_{m1}^v)}{\partial H_{f2}^v}\right|_{(\mathbf{u}^*,x^*)} \\
& -(p_{31}-p_{30})\left.\frac{\partial(\lambda_{f2}S_{m2}^u)}{\partial H_{f2}^v}\right|_{(\mathbf{u}^*,x^*)} -(p_{33}-p_{32})(1-\varepsilon_{m2})\left.\frac{\partial(\lambda_{f2}S_{m2}^v)}{\partial H_{f2}^v}\right|_{(\mathbf{u}^*,x^*)}, \qquad \text{(S10.21)}
\end{aligned}
$$

$$
\begin{aligned}
p_{22}^{'} =& -\frac{\partial \mathscr{H}(t,\mathbf{u}^*,x^*,p)}{\partial C_{f2}^v} \\
=& -a_2\kappa_2\left\{E_1[\Lambda+(1-\Lambda)\sigma_{f2}^{v*}(t)]\right\}^2C_{f2}^{v*}(t)-a_2\kappa_3\left\{T_C[\Lambda+(1-\Lambda)\sigma_{f2}^{v*}(t)]\right\}^2C_{f2}^{v*}(t) \\
& -(p_{14}-p_{13})\left.\frac{\partial(\lambda_{m2}S_{f2}^u)}{\partial C_{f2}^v}\right|_{(\mathbf{u}^*,x^*)} -(p_{19}-p_{18})(1-\varepsilon_{f2})\left.\frac{\partial(\lambda_{m2}S_{f2}^v)}{\partial C_{f2}^v}\right|_{(\mathbf{u}^*,x^*)} \\
& +p_{22}[\Lambda+(1-\Lambda)\sigma_{f2}^{v*}(t)+\mu_{f2}]-p_{23}[\Lambda+(1-\Lambda)\sigma_{f2}^{v*}(t)]
\end{aligned}
$$

$$
\begin{aligned}
&- (p_{26} - p_{25}) \left.\frac{\partial(\lambda_{f1} S^u_{m1})}{\partial C^v_{f2}}\right|_{(\mathbf{u}^*, x^*)} - (p_{28} - p_{27})(1 - \varepsilon_{m1}) \left.\frac{\partial(\lambda_{f1} S^v_{m1})}{\partial C^v_{f2}}\right|_{(\mathbf{u}^*, x^*)} \\
&- (p_{31} - p_{30}) \left.\frac{\partial(\lambda_{f2} S^u_{m2})}{\partial C^v_{f2}}\right|_{(\mathbf{u}^*, x^*)} - (p_{33} - p_{32})(1 - \varepsilon_{m2}) \left.\frac{\partial(\lambda_{f2} S^v_{m2})}{\partial C^v_{f2}}\right|_{(\mathbf{u}^*, x^*)}, \quad \text{(S10.22)}
\end{aligned}
$$

$$
\begin{aligned}
p_{23}^{'} = &- \frac{\partial \mathscr{H}(t, \mathbf{u}^*, x^*, p)}{\partial D_{f2}} \\
= &- (p_{14} - p_{13}) \left.\frac{\partial(\lambda_{m2} S^u_{f2})}{\partial D_{f2}}\right|_{(\mathbf{u}^*, x^*)} - (p_{19} - p_{18})(1 - \varepsilon_{f2}) \left.\frac{\partial(\lambda_{m2} S^v_{f2})}{\partial D_{f2}}\right|_{(\mathbf{u}^*, x^*)} \\
&+ p_{23}(\gamma_{f2} + d_{f2} + \mu_{f2}) - p_{24}\gamma_{f2}, \quad \text{(S10.23)}
\end{aligned}
$$

$$
\begin{aligned}
p_{24}^{'} = &- \frac{\partial \mathscr{H}(t, \mathbf{u}^*, x^*, p)}{\partial R_{f2}} \\
= &- a_2 \kappa_2 (E_2 \sigma^{u*}_{f2}(t))^2 (S^{u*}_{f2}(t) + I^{u*}_{f2}(t) + R^*_{f2}(t)) - p_{13}\zeta_{f2} + p_{24}(\mu_{f2} + \zeta_{f2}) \\
&- (p_{14} - p_{13}) \left.\frac{\partial(\lambda_{m2} S^u_{f2})}{\partial R_{f2}}\right|_{(\mathbf{u}^*, x^*)} - (p_{19} - p_{18})(1 - \varepsilon_{f2}) \left.\frac{\partial(\lambda_{m2} S^v_{f2})}{\partial R_{f2}}\right|_{(\mathbf{u}^*, x^*)}, \quad \text{(S10.24)}
\end{aligned}
$$

$$
\begin{aligned}
p_{25}^{'} = &- \frac{\partial \mathscr{H}(t, \mathbf{u}^*, x^*, p)}{\partial S^u_{m1}} \\
= &- a_2 \kappa_1 (B_2 k^*_m(t))^2 S^{u*}_{m1}(t) \\
&- (p_{26} - p_{25}) \left.\frac{\partial(\lambda_{f1} S^u_{m1})}{\partial S^u_{m1}}\right|_{(\mathbf{u}^*, x^*)} + p_{25}(k^*_m(t) + \mu_{m1} + \alpha_m) - p_{27} k^*_m(t) \\
&- (p_{28} - p_{27})(1 - \varepsilon_{m1}) \left.\frac{\partial(\lambda_{f1} S^v_{m1})}{\partial S^u_{m1}}\right|_{(\mathbf{u}^*, x^*)} - p_{30}\alpha_m, \quad \text{(S10.25)}
\end{aligned}
$$

$$
\begin{aligned}
p_{26}^{'} = &- \frac{\partial \mathscr{H}(t, \mathbf{u}^*, x^*, p)}{\partial I^u_{m1}} \\
= &- (p_2 - p_1) \left.\frac{\partial(\lambda_{m1} S^u_{f1})}{\partial I^u_{m1}}\right|_{(\mathbf{u}^*, x^*)} - (p_7 - p_6)(1 - \varepsilon_{f1}) \left.\frac{\partial(\lambda_{m1} S^v_{f1})}{\partial I^u_{m1}}\right|_{(\mathbf{u}^*, x^*)} \\
&- (p_{14} - p_{13}) \left.\frac{\partial(\lambda_{m2} S^u_{f2})}{\partial I^u_{m1}}\right|_{(\mathbf{u}^*, x^*)} - (p_{19} - p_{18})(1 - \varepsilon_{f2}) \left.\frac{\partial(\lambda_{m2} S^v_{f2})}{\partial I^u_{m1}}\right|_{(\mathbf{u}^*, x^*)} \\
&- (p_{26} - p_{25}) \left.\frac{\partial(\lambda_{f1} S^u_{m1})}{\partial I^u_{m1}}\right|_{(\mathbf{u}^*, x^*)} + p_{26}(\omega^u_{m1} + \mu_{m1} + \alpha_m) \\
&- (p_{28} - p_{27})(1 - \varepsilon_{m1}) \left.\frac{\partial(\lambda_{f1} S^v_{m1})}{\partial I^u_{m1}}\right|_{(\mathbf{u}^*, x^*)} - p_{29}\omega^u_{m1} - p_{31}\alpha_m, \quad \text{(S10.26)}
\end{aligned}
$$

$$
\begin{aligned}
p_{27}^{'} = &- \frac{\partial \mathscr{H}(t, \mathbf{u}^*, x^*, p)}{\partial S^v_{m1}} \\
= &- (p_{26} - p_{25}) \left.\frac{\partial(\lambda_{f1} S^u_{m1})}{\partial S^v_{m1}}\right|_{(\mathbf{u}^*, x^*)} - p_{25}\delta + p_{27}(\delta + \mu_{m1} + \alpha_m) - p_{32}\alpha_m \\
&- (p_{28} - p_{27})(1 - \varepsilon_{m1}) \left.\frac{\partial(\lambda_{f1} S^v_{m1})}{\partial S^v_{m1}}\right|_{(\mathbf{u}^*, x^*)}, \quad \text{(S10.27)}
\end{aligned}
$$

$$
\begin{aligned}
p_{28}^{'} = &- \frac{\partial \mathscr{H}(t, \mathbf{u}^*, x^*, p)}{\partial I^v_{m1}} \\
= &- (p_2 - p_1) \left.\frac{\partial(\lambda_{m1} S^u_{f1})}{\partial I^v_{m1}}\right|_{(\mathbf{u}^*, x^*)} - (p_7 - p_6)(1 - \varepsilon_{f1}) \left.\frac{\partial(\lambda_{m1} S^v_{f1})}{\partial I^v_{m1}}\right|_{(\mathbf{u}^*, x^*)}
\end{aligned}
$$

$$
\begin{aligned}
&- (p_{14}-p_{13})\left.\frac{\partial(\lambda_{m2}S^u_{f2})}{\partial I^v_{m1}}\right|_{(\mathbf{u}^*,x^*)} - (p_{19}-p_{18})(1-\varepsilon_{f2})\left.\frac{\partial(\lambda_{m2}S^v_{f2})}{\partial I^v_{m1}}\right|_{(\mathbf{u}^*,x^*)}\\
&+ p_{28}(\omega^v_{m1}+\mu_{m1}+\alpha_m) - p_{29}\omega^v_{m1} - p_{33}\alpha_m\\
&- (p_{26}-p_{25})\left.\frac{\partial(\lambda_{f1}S^u_{m1})}{\partial I^v_{m1}}\right|_{(\mathbf{u}^*,x^*)} - (p_{28}-p_{27})(1-\varepsilon_{m1})\left.\frac{\partial(\lambda_{f1}S^v_{m1})}{\partial I^v_{m1}}\right|_{(\mathbf{u}^*,x^*)}, \quad \text{(S10.28)}
\end{aligned}
$$

$$
\begin{aligned}
p'_{29} =& -\frac{\partial\mathscr{H}(t,\mathbf{u}^*,x^*,p)}{\partial R_{m1}}\\
=& -(p_{26}-p_{25})\left.\frac{\partial(\lambda_{f1}S^u_{m1})}{\partial R_{m1}}\right|_{(\mathbf{u}^*,x^*)} - (p_{28}-p_{27})(1-\varepsilon_{m1})\left.\frac{\partial(\lambda_{f1}S^v_{m1})}{\partial R_{m1}}\right|_{(\mathbf{u}^*,x^*)}\\
&- p_{25}\zeta_{m1} + p_{29}(\mu_{m1}+\alpha_m+\zeta_{m1}) - p_{34}\alpha_m, \quad \text{(S10.29)}
\end{aligned}
$$

$$
\begin{aligned}
p'_{30} =& -\frac{\partial\mathscr{H}(t,\mathbf{u}^*,x^*,p)}{\partial S^u_{m2}}\\
=& -(p_{31}-p_{30})\left.\frac{\partial(\lambda_{f2}S^u_{m2})}{\partial S^u_{m2}}\right|_{(\mathbf{u}^*,x^*)} + p_{30}\mu_{m2} - (p_{33}-p_{32})(1-\varepsilon_{m2})\left.\frac{\partial(\lambda_{f2}S^v_{m2})}{\partial S^u_{m2}}\right|_{(\mathbf{u}^*,x^*)}, \quad \text{(S10.30)}
\end{aligned}
$$

$$
\begin{aligned}
p'_{31} =& -\frac{\partial\mathscr{H}(t,\mathbf{u}^*,x^*,p)}{\partial I^u_{m2}}\\
=& -(p_{2}-p_{1})\left.\frac{\partial(\lambda_{m1}S^u_{f1})}{\partial I^u_{m2}}\right|_{(\mathbf{u}^*,x^*)} - (p_{7}-p_{6})(1-\varepsilon_{f1})\left.\frac{\partial(\lambda_{m1}S^v_{f1})}{\partial I^u_{m2}}\right|_{(\mathbf{u}^*,x^*)}\\
&- (p_{14}-p_{13})\left.\frac{\partial(\lambda_{m2}S^u_{f2})}{\partial I^u_{m2}}\right|_{(\mathbf{u}^*,x^*)} - (p_{19}-p_{18})(1-\varepsilon_{f2})\left.\frac{\partial(\lambda_{m2}S^v_{f2})}{\partial I^u_{m2}}\right|_{(\mathbf{u}^*,x^*)}\\
&- (p_{31}-p_{30})\left.\frac{\partial(\lambda_{f2}S^u_{m2})}{\partial I^u_{m2}}\right|_{(\mathbf{u}^*,x^*)} - (p_{33}-p_{32})(1-\varepsilon_{m2})\left.\frac{\partial(\lambda_{f2}S^v_{m2})}{\partial I^u_{m2}}\right|_{(\mathbf{u}^*,x^*)}\\
&+ p_{31}(\omega^u_{m2}+\mu_{m2}) - p_{34}\omega^u_{m2}, \quad \text{(S10.31)}
\end{aligned}
$$

$$
\begin{aligned}
p'_{32} =& -\frac{\partial\mathscr{H}(t,\mathbf{u}^*,x^*,p)}{\partial S^v_{m2}}\\
=& -(p_{31}-p_{30})\left.\frac{\partial(\lambda_{f2}S^u_{m2})}{\partial S^v_{m2}}\right|_{(\mathbf{u}^*,x^*)} - p_{30}\delta + p_{32}(\delta+\mu_{m2})\\
&- (p_{33}-p_{32})(1-\varepsilon_{m2})\left.\frac{\partial(\lambda_{f2}S^v_{m2})}{\partial S^v_{m2}}\right|_{(\mathbf{u}^*,x^*)}, \quad \text{(S10.32)}
\end{aligned}
$$

$$
\begin{aligned}
p'_{33} =& -\frac{\partial\mathscr{H}(t,\mathbf{u}^*,x^*,p)}{\partial I^v_{m2}}\\
=& -(p_{2}-p_{1})\left.\frac{\partial(\lambda_{m1}S^u_{f1})}{\partial I^v_{m2}}\right|_{(\mathbf{u}^*,x^*)} - (p_{7}-p_{6})(1-\varepsilon_{f1})\left.\frac{\partial(\lambda_{m1}S^v_{f1})}{\partial I^v_{m2}}\right|_{(\mathbf{u}^*,x^*)}\\
&- (p_{14}-p_{13})\left.\frac{\partial(\lambda_{m2}S^u_{f2})}{\partial I^v_{m2}}\right|_{(\mathbf{u}^*,x^*)} - (p_{19}-p_{18})(1-\varepsilon_{f2})\left.\frac{\partial(\lambda_{m2}S^v_{f2})}{\partial I^v_{m2}}\right|_{(\mathbf{u}^*,x^*)}\\
&- (p_{31}-p_{30})\left.\frac{\partial(\lambda_{f2}S^u_{m2})}{\partial I^v_{m2}}\right|_{(\mathbf{u}^*,x^*)} - (p_{33}-p_{32})(1-\varepsilon_{m2})\left.\frac{\partial(\lambda_{f2}S^v_{m2})}{\partial I^v_{m2}}\right|_{(\mathbf{u}^*,x^*)}\\
&+ p_{33}(\omega^v_{m2}+\mu_{m2}) - p_{34}\omega^v_{m2}, \quad \text{(S10.33)}
\end{aligned}
$$

$$
p'_{34} = -\frac{\partial\mathscr{H}(t,\mathbf{u}^*,x^*,p)}{\partial R_{m2}}
$$

$$= -p_{30}\zeta_{m2} - (p_{31}-p_{30})\,\frac{\partial(\lambda_{f2}S^u_{m2})}{\partial R_{m2}}\Big|_{(\mathbf{u}^*,x^*)}$$
$$-(p_{33}-p_{32})(1-\varepsilon_{m2})\,\frac{\partial(\lambda_{f2}S^v_{m2})}{\partial R_{m2}}\Big|_{(\mathbf{u}^*,x^*)} + p_{34}(\mu_{m2}+\zeta_{m2}), \quad \text{(S10.34)}$$

where

$$\frac{\partial(\lambda_{m1}S^u_{f1})}{\partial S^u_{f1}} = [\beta_m c_{m11}(I^u_{m1}+I^v_{m1}) + \beta_m c_{m21}(I^u_{m2}+I^v_{m2})]\frac{N_{f1}-S^u_{f1}}{N^2_{f1}},$$
$$\frac{\partial(\lambda_{m1}S^u_{f1})}{\partial I^u_{f1}} = \frac{\partial(\lambda_{m1}S^u_{f1})}{\partial L^u_{f1}} = \frac{\partial(\lambda_{m1}S^u_{f1})}{\partial H^u_{f1}} = \frac{\partial(\lambda_{m1}S^u_{f1})}{\partial C^u_{f1}} = \frac{\partial(\lambda_{m1}S^u_{f1})}{\partial S^v_{f1}}$$
$$= \frac{\partial(\lambda_{m1}S^u_{f1})}{\partial I^v_{f1}} = \frac{\partial(\lambda_{m1}S^u_{f1})}{\partial L^v_{f1}} = \frac{\partial(\lambda_{m1}S^u_{f1})}{\partial H^v_{f1}} = \frac{\partial(\lambda_{m1}S^u_{f1})}{\partial C^v_{f1}} = \frac{\partial(\lambda_{m1}S^u_{f1})}{\partial D_{f1}}$$
$$= \frac{\partial(\lambda_{m1}S^u_{f1})}{\partial R_{f1}} = -[\beta_m c_{m11}(I^u_{m1}+I^v_{m1}) + \beta_m c_{m21}(I^u_{m2}+I^v_{m2})]\frac{S^u_{f1}}{N^2_{f1}},$$
$$\frac{\partial(\lambda_{m1}S^v_{f1})}{\partial S^v_{f1}} = [\beta_m c_{m11}(I^u_{m1}+I^v_{m1}) + \beta_m c_{m21}(I^u_{m2}+I^v_{m2})]\frac{N_{f1}-S^v_{f1}}{N^2_{f1}},$$
$$\frac{\partial(\lambda_{m1}S^v_{f1})}{\partial S^u_{f1}} = \frac{\partial(\lambda_{m1}S^v_{f1})}{\partial I^u_{f1}} = \frac{\partial(\lambda_{m1}S^v_{f1})}{\partial L^u_{f1}} = \frac{\partial(\lambda_{m1}S^v_{f1})}{\partial H^u_{f1}} = \frac{\partial(\lambda_{m1}S^v_{f1})}{\partial C^u_{f1}}$$
$$= \frac{\partial(\lambda_{m1}S^v_{f1})}{\partial I^v_{f1}} = \frac{\partial(\lambda_{m1}S^v_{f1})}{\partial L^v_{f1}} = \frac{\partial(\lambda_{m1}S^v_{f1})}{\partial H^v_{f1}} = \frac{\partial(\lambda_{m1}S^v_{f1})}{\partial C^v_{f1}} = \frac{\partial(\lambda_{m1}S^v_{f1})}{\partial D_{f1}}$$
$$= \frac{\partial(\lambda_{m1}S^v_{f1})}{\partial R_{f1}} = -[\beta_m c_{m11}(I^u_{m1}+I^v_{m1}) + \beta_m c_{m21}(I^u_{m2}+I^v_{m2})]\frac{S^v_{f1}}{N^2_{f1}},$$
$$\frac{\partial(\lambda_{f1}S^u_{m1})}{\partial I^u_{f1}} = \frac{\partial(\lambda_{f1}S^u_{m1})}{\partial L^u_{f1}} = \frac{\partial(\lambda_{f1}S^u_{m1})}{\partial H^u_{f1}} = \frac{\partial(\lambda_{f1}S^u_{m1})}{\partial C^u_{f1}}$$
$$= \frac{\partial(\lambda_{f1}S^u_{m1})}{\partial I^v_{f1}} = \frac{\partial(\lambda_{f1}S^u_{m1})}{\partial L^v_{f1}} = \frac{\partial(\lambda_{f1}S^u_{m1})}{\partial H^v_{f1}} = \frac{\partial(\lambda_{f1}S^u_{m1})}{\partial C^v_{f1}} = \frac{\beta_f c_{f11}S^u_{m1}}{N_{m1}},$$
$$\frac{\partial(\lambda_{f1}S^v_{m1})}{\partial I^u_{f1}} = \frac{\partial(\lambda_{f1}S^v_{m1})}{\partial L^u_{f1}} = \frac{\partial(\lambda_{f1}S^v_{m1})}{\partial H^u_{f1}} = \frac{\partial(\lambda_{f1}S^v_{m1})}{\partial C^u_{f1}}$$
$$= \frac{\partial(\lambda_{f1}S^v_{m1})}{\partial I^v_{f1}} = \frac{\partial(\lambda_{f1}S^v_{m1})}{\partial L^v_{f1}} = \frac{\partial(\lambda_{f1}S^v_{m1})}{\partial H^v_{f1}} = \frac{\partial(\lambda_{f1}S^v_{m1})}{\partial C^v_{f1}} = \frac{\beta_f c_{f11}S^v_{m1}}{N_{m1}},$$
$$\frac{\partial(\lambda_{f2}S^u_{m2})}{\partial I^u_{f1}} = \frac{\partial(\lambda_{f2}S^u_{m2})}{\partial L^u_{f1}} = \frac{\partial(\lambda_{f2}S^u_{m2})}{\partial H^u_{f1}} = \frac{\partial(\lambda_{f2}S^u_{m2})}{\partial C^u_{f1}}$$
$$= \frac{\partial(\lambda_{f2}S^u_{m2})}{\partial I^v_{f1}} = \frac{\partial(\lambda_{f2}S^u_{m2})}{\partial L^v_{f1}} = \frac{\partial(\lambda_{f2}S^u_{m2})}{\partial H^v_{f1}} = \frac{\partial(\lambda_{f2}S^u_{m2})}{\partial C^v_{f1}} = \frac{\beta_f c_{f12}S^u_{m2}}{N_{m2}},$$
$$\frac{\partial(\lambda_{f2}S^v_{m2})}{\partial I^u_{f1}} = \frac{\partial(\lambda_{f2}S^v_{m2})}{\partial L^u_{f1}} = \frac{\partial(\lambda_{f2}S^v_{m2})}{\partial H^u_{f1}} = \frac{\partial(\lambda_{f2}S^v_{m2})}{\partial C^u_{f1}}$$
$$= \frac{\partial(\lambda_{f2}S^v_{m2})}{\partial I^v_{f1}} = \frac{\partial(\lambda_{f2}S^v_{m2})}{\partial L^v_{f1}} = \frac{\partial(\lambda_{f2}S^v_{m2})}{\partial H^v_{f1}} = \frac{\partial(\lambda_{f2}S^v_{m2})}{\partial C^v_{f1}} = \frac{\beta_f c_{f12}S^v_{m2}}{N_{m2}},$$
$$\frac{\partial(\lambda_{m2}S^u_{f2})}{\partial S^u_{f2}} = [\beta_m c_{m12}(I^u_{m1}+I^v_{m1}) + \beta_m c_{m22}(I^u_{m2}+I^v_{m2})]\frac{N_{f2}-S^u_{f2}}{N^2_{f2}},$$
$$\frac{\partial(\lambda_{m2}S^u_{f2})}{\partial I^u_{f2}} = \frac{\partial(\lambda_{m2}S^u_{f2})}{\partial L^u_{f2}} = \frac{\partial(\lambda_{m2}S^u_{f2})}{\partial H^u_{f2}} = \frac{\partial(\lambda_{m2}S^u_{f2})}{\partial C^u_{f2}} = \frac{\partial(\lambda_{m2}S^u_{f2})}{\partial S^v_{f2}}$$

$$
\begin{aligned}
&=\frac{\partial(\lambda_{m2}S^u_{f2})}{\partial I^v_{f2}} = \frac{\partial(\lambda_{m2}S^u_{f2})}{\partial L^v_{f2}} = \frac{\partial(\lambda_{m2}S^u_{f2})}{\partial H^v_{f2}} = \frac{\partial(\lambda_{m2}S^u_{f2})}{\partial C^v_{f2}} = \frac{\partial(\lambda_{m2}S^u_{f2})}{\partial D_{f2}} \\
&=\frac{\partial(\lambda_{m2}S^u_{f2})}{\partial R_{f2}} = -[\beta_m c_{m12}(I^u_{m1}+I^v_{m1}) + \beta_m c_{m22}(I^u_{m2}+I^v_{m2})]\frac{S^u_{f2}}{N^2_{f2}}, \\
&\frac{\partial(\lambda_{m2}S^v_{f2})}{\partial S^v_{f2}} = [\beta_m c_{m12}(I^u_{m1}+I^v_{m1}) + \beta_m c_{m22}(I^u_{m2}+I^v_{m2})]\frac{N_{f2}-S^v_{f2}}{N^2_{f2}}, \\
&\frac{\partial(\lambda_{m2}S^v_{f2})}{\partial S^u_{f2}} = \frac{\partial(\lambda_{m2}S^v_{f2})}{\partial I^u_{f2}} = \frac{\partial(\lambda_{m2}S^v_{f2})}{\partial L^u_{f2}} = \frac{\partial(\lambda_{m2}S^v_{f2})}{\partial H^u_{f2}} = \frac{\partial(\lambda_{m2}S^v_{f2})}{\partial C^u_{f2}} \\
&=\frac{\partial(\lambda_{m2}S^v_{f2})}{\partial I^v_{f2}} = \frac{\partial(\lambda_{m2}S^v_{f2})}{\partial L^v_{f2}} = \frac{\partial(\lambda_{m2}S^v_{f2})}{\partial H^v_{f2}} = \frac{\partial(\lambda_{m2}S^v_{f2})}{\partial C^v_{f2}} = \frac{\partial(\lambda_{m2}S^v_{f2})}{\partial D_{f2}} \\
&=\frac{\partial(\lambda_{m2}S^v_{f2})}{\partial R_{f2}} = -[\beta_m c_{m12}(I^u_{m1}+I^v_{m1}) + \beta_m c_{m22}(I^u_{m2}+I^v_{m2})]\frac{S^v_{f2}}{N^2_{f2}}, \\
&\frac{\partial(\lambda_{f1}S^u_{m1})}{\partial I^u_{f2}} = \frac{\partial(\lambda_{f1}S^u_{m1})}{\partial L^u_{f2}} = \frac{\partial(\lambda_{f1}S^u_{m1})}{\partial H^u_{f2}} = \frac{\partial(\lambda_{f1}S^u_{m1})}{\partial C^u_{f2}} \\
&=\frac{\partial(\lambda_{f1}S^u_{m1})}{\partial I^v_{f2}} = \frac{\partial(\lambda_{f1}S^u_{m1})}{\partial L^v_{f2}} = \frac{\partial(\lambda_{f1}S^u_{m1})}{\partial H^v_{f2}} = \frac{\partial(\lambda_{f1}S^u_{m1})}{\partial C^v_{f2}} = \frac{\beta_f c_{f21} S^u_{m1}}{N_{m1}}, \\
&\frac{\partial(\lambda_{f1}S^v_{m1})}{\partial I^u_{f2}} = \frac{\partial(\lambda_{f1}S^v_{m1})}{\partial L^u_{f2}} = \frac{\partial(\lambda_{f1}S^v_{m1})}{\partial H^u_{f2}} = \frac{\partial(\lambda_{f1}S^v_{m1})}{\partial C^u_{f2}} \\
&=\frac{\partial(\lambda_{f1}S^v_{m1})}{\partial I^v_{f2}} = \frac{\partial(\lambda_{f1}S^v_{m1})}{\partial L^v_{f2}} = \frac{\partial(\lambda_{f1}S^v_{m1})}{\partial H^v_{f2}} = \frac{\partial(\lambda_{f1}S^v_{m1})}{\partial C^v_{f2}} = \frac{\beta_f c_{f21} S^v_{m1}}{N_{m1}}, \\
&\frac{\partial(\lambda_{f2}S^u_{m2})}{\partial I^u_{f2}} = \frac{\partial(\lambda_{f2}S^u_{m2})}{\partial L^u_{f2}} = \frac{\partial(\lambda_{f2}S^u_{m2})}{\partial H^u_{f2}} = \frac{\partial(\lambda_{f2}S^u_{m2})}{\partial C^u_{f2}} \\
&=\frac{\partial(\lambda_{f2}S^u_{m2})}{\partial I^v_{f2}} = \frac{\partial(\lambda_{f2}S^u_{m2})}{\partial L^v_{f2}} = \frac{\partial(\lambda_{f2}S^u_{m2})}{\partial H^v_{f2}} = \frac{\partial(\lambda_{f2}S^u_{m2})}{\partial C^v_{f2}} = \frac{\beta_f c_{f22} S^u_{m2}}{N_{m2}}, \\
&\frac{\partial(\lambda_{f2}S^v_{m2})}{\partial I^u_{f2}} = \frac{\partial(\lambda_{f2}S^v_{m2})}{\partial L^u_{f2}} = \frac{\partial(\lambda_{f2}S^v_{m2})}{\partial H^u_{f2}} = \frac{\partial(\lambda_{f2}S^v_{m2})}{\partial C^u_{f2}} \\
&=\frac{\partial(\lambda_{f2}S^v_{m2})}{\partial I^v_{f2}} = \frac{\partial(\lambda_{f2}S^v_{m2})}{\partial L^v_{f2}} = \frac{\partial(\lambda_{f2}S^v_{m2})}{\partial H^v_{f2}} = \frac{\partial(\lambda_{f2}S^v_{m2})}{\partial C^v_{f2}} = \frac{\beta_f c_{f22} S^v_{m2}}{N_{m2}}, \\
&\frac{\partial(\lambda_{f1}S^u_{m1})}{\partial S^u_{m1}} = \left[\beta_f c_{f11}\left(\sum_{h\in\{u,v\}} I^h_{f1}+L^h_{f1}+H^h_{f1}+C^h_{f1}\right)\right. \\
&\qquad\qquad \left. + \beta_f c_{f21}\left(\sum_{h\in\{u,v\}} I^h_{f2}+L^h_{f2}+H^h_{f2}+C^h_{f2}\right)\right]\frac{N_{m1}-S^u_{m1}}{N^2_{m1}}, \\
&\frac{\partial(\lambda_{f1}S^v_{m1})}{\partial S^v_{m1}} = \left[\beta_f c_{f11}\left(\sum_{h\in\{u,v\}} I^h_{f1}+L^h_{f1}+H^h_{f1}+C^h_{f1}\right)\right. \\
&\qquad\qquad \left. + \beta_f c_{f21}\left(\sum_{h\in\{u,v\}} I^h_{f2}+L^h_{f2}+H^h_{f2}+C^h_{f2}\right)\right]\frac{N_{m1}-S^v_{m1}}{N^2_{m1}}, \\
&\frac{\partial(\lambda_{f2}S^u_{m2})}{\partial S^u_{m2}} = \left[\beta_f c_{f12}\left(\sum_{h\in\{u,v\}} I^h_{f1}+L^h_{f1}+H^h_{f1}+C^h_{f1}\right)\right.
\end{aligned}
$$

$$
\begin{aligned}
&+\beta_f c_{f22}\left(\sum_{h\in\{u,v\}} I^h_{f2}+L^h_{f2}+H^h_{f2}+C^h_{f2}\right)\Bigg]\frac{N_{m2}-S^u_{m2}}{N^2_{m2}},\\
\frac{\partial(\lambda_{f2}S^v_{m2})}{\partial S^v_{m2}} &= \Bigg[\beta_f c_{f12}\left(\sum_{h\in\{u,v\}} I^h_{f1}+L^h_{f1}+H^h_{f1}+C^h_{f1}\right)\\
&+\beta_f c_{f22}\left(\sum_{h\in\{u,v\}} I^h_{f2}+L^h_{f2}+H^h_{f2}+C^h_{f2}\right)\Bigg]\frac{N_{m2}-S^v_{m2}}{N^2_{m2}},
\end{aligned}
$$

$$
\begin{aligned}
&\frac{\partial(\lambda_{f1}S^u_{m1})}{\partial I^u_{m1}} = \frac{\partial(\lambda_{f1}S^u_{m1})}{\partial S^v_{m1}} = \frac{\partial(\lambda_{f1}S^u_{m1})}{\partial I^v_{m1}} = \frac{\partial(\lambda_{f1}S^u_{m1})}{\partial R_{m1}}\\
&= -\Bigg[\beta_f c_{f11}\left(\sum_{h\in\{u,v\}} I^h_{f1}+L^h_{f1}+H^h_{f1}+C^h_{f1}\right)\\
&\quad +\beta_f c_{f21}\left(\sum_{h\in\{u,v\}} I^h_{f2}+L^h_{f2}+H^h_{f2}+C^h_{f2}\right)\Bigg]\frac{S^u_{m1}}{N^2_{m1}},
\end{aligned}
$$

$$
\begin{aligned}
&\frac{\partial(\lambda_{f1}S^v_{m1})}{\partial S^u_{m1}} = \frac{\partial(\lambda_{f1}S^v_{m1})}{\partial I^u_{m1}} = \frac{\partial(\lambda_{f1}S^v_{m1})}{\partial I^v_{m1}} = \frac{\partial(\lambda_{f1}S^v_{m1})}{\partial R_{m1}}\\
&= -\Bigg[\beta_f c_{f11}\left(\sum_{h\in\{u,v\}} I^h_{f1}+L^h_{f1}+H^h_{f1}+C^h_{f1}\right)\\
&\quad +\beta_f c_{f21}\left(\sum_{h\in\{u,v\}} I^h_{f2}+L^h_{f2}+H^h_{f2}+C^h_{f2}\right)\Bigg]\frac{S^v_{m1}}{N^2_{m1}},
\end{aligned}
$$

$$
\begin{aligned}
&\frac{\partial(\lambda_{f2}S^u_{m2})}{\partial I^u_{m2}} = \frac{\partial(\lambda_{f2}S^u_{m2})}{\partial S^v_{m2}} = \frac{\partial(\lambda_{f2}S^u_{m2})}{\partial I^v_{m2}} = \frac{\partial(\lambda_{f2}S^u_{m2})}{\partial R_{m2}}\\
&= -\Bigg[\beta_f c_{f12}\left(\sum_{h\in\{u,v\}} I^h_{f1}+L^h_{f1}+H^h_{f1}+C^h_{f1}\right)\\
&\quad +\beta_f c_{f22}\left(\sum_{h\in\{u,v\}} I^h_{f2}+L^h_{f2}+H^h_{f2}+C^h_{f2}\right)\Bigg\}\frac{S^u_{m2}}{N^2_{m2}},
\end{aligned}
$$

$$
\begin{aligned}
&\frac{\partial(\lambda_{f2}S^v_{m2})}{\partial S^u_{m2}} = \frac{\partial(\lambda_{f2}S^v_{m2})}{\partial I^u_{m2}} = \frac{\partial(\lambda_{f2}S^v_{m2})}{\partial I^v_{m2}} = \frac{\partial(\lambda_{f2}S^v_{m2})}{\partial R_{m2}}\\
&= -\Bigg\{\beta_f c_{f12}\left(\sum_{h\in\{u,v\}} I^h_{f1}+L^h_{f1}+H^h_{f1}+C^h_{f1}\right)\\
&\quad +\beta_f c_{f22}\left(\sum_{h\in\{u,v\}} I^h_{f2}+L^h_{f2}+H^h_{f2}+C^h_{f2}\right)\Bigg\}\frac{S^v_{m2}}{N^2_{m2}},
\end{aligned}
$$

$$
\begin{aligned}
\frac{\partial(\lambda_{m1}S^u_{f1})}{\partial I^u_{m1}} &= \frac{\partial(\lambda_{m1}S^u_{f1})}{\partial I^v_{m1}} = \frac{\beta_m c_{m11}S^u_{f1}}{N_{f1}},\\
\frac{\partial(\lambda_{m1}S^v_{f1})}{\partial I^u_{m1}} &= \frac{\partial(\lambda_{m1}S^v_{f1})}{\partial I^v_{m1}} = \frac{\beta_m c_{m11}S^v_{f1}}{N_{f1}},\\
\frac{\partial(\lambda_{m2}S^u_{f2})}{\partial I^u_{m1}} &= \frac{\partial(\lambda_{m2}S^u_{f2})}{\partial I^v_{m1}} = \frac{\beta_m c_{m12}S^u_{f2}}{N_{f2}},
\end{aligned}
$$

$$\frac{\partial(\lambda_{m2}S^v_{f2})}{\partial I^u_{m1}} = \frac{\partial(\lambda_{m2}S^v_{f2})}{\partial I^v_{m1}} = \frac{\beta_m c_{m12} S^v_{f2}}{N_{f2}},$$
$$\frac{\partial(\lambda_{m1}S^u_{f1})}{\partial I^u_{m2}} = \frac{\partial(\lambda_{m1}S^u_{f1})}{\partial I^v_{m2}} = \frac{\beta_m c_{m21} S^u_{f1}}{N_{f1}},$$
$$\frac{\partial(\lambda_{m1}S^v_{f1})}{\partial I^u_{m2}} = \frac{\partial(\lambda_{m1}S^v_{f1})}{\partial I^v_{m2}} = \frac{\beta_m c_{m21} S^v_{f1}}{N_{f1}},$$
$$\frac{\partial(\lambda_{m2}S^u_{f2})}{\partial I^u_{m2}} = \frac{\partial(\lambda_{m2}S^u_{f2})}{\partial I^v_{m2}} = \frac{\beta_m c_{m22} S^u_{f2}}{N_{f2}},$$
$$\frac{\partial(\lambda_{m2}S^v_{f2})}{\partial I^u_{m2}} = \frac{\partial(\lambda_{m2}S^v_{f2})}{\partial I^v_{m2}} = \frac{\beta_m c_{m22} S^v_{f2}}{N_{f2}}.$$

And

$$\begin{aligned}
&p_1(T) = \phi_{S^u_{f1}}(x^*(T)) = 0, \qquad p_2(T) = \phi_{I^u_{f1}}(x^*(T)) = 0,\\
&p_3(T) = \phi_{L^u_{f1}}(x^*(T)) = 0, \qquad p_4(T) = \phi_{H^u_{f1}}(x^*(T)) = 0,\\
&p_5(T) = \phi_{C^u_{f1}}(x^*(T)) = a_1(C^{u*}_{f1}(T) + C^{v*}_{f1}(T) + C^{u*}_{f2}(T) + C^{v*}_{f2}(T) - \mathbb{I});\\
&p_6(T) = \phi_{S^v_{f1}}(x^*(T)) = 0, \qquad p_7(T) = \phi_{I^v_{f1}}(x^*(T)) = 0,\\
&p_8(T) = \phi_{L^v_{f1}}(x^*(T)) = 0, \qquad p_9(T) = \phi_{H^v_{f1}}(x^*(T)) = 0,\\
&p_{10}(T) = \phi_{C^v_{f1}}(x^*(T)) = a_1(C^{u*}_{f1}(T) + C^{v*}_{f1}(T) + C^{u*}_{f2}(T) + C^{v*}_{f2}(T) - \mathbb{I});\\
&p_{11}(T) = \phi_{D_{f1}}(x^*(T)) = 0, \qquad p_{12}(T) = \phi_{R_{f1}}(x^*(T)) = 0,\\
&p_{13}(T) = \phi_{S^u_{f2}}(x^*(T)) = 0, \qquad p_{14}(T) = \phi_{I^u_{f2}}(x^*(T)) = 0,\\
&p_{15}(T) = \phi_{L^u_{f2}}(x^*(T)) = 0, \qquad p_{16}(T) = \phi_{H^u_{f2}}(x^*(T)) = 0,\\
&p_{17}(T) = \phi_{C^u_{f2}}(x^*(T)) = a_1(C^{u*}_{f1}(T) + C^{v*}_{f1}(T) + C^{u*}_{f2}(T) + C^{v*}_{f2}(T) - \mathbb{I});\\
&p_{18}(T) = \phi_{S^v_{f2}}(x^*(T)) = 0, \qquad p_{19}(T) = \phi_{I^v_{f2}}(x^*(T)) = 0,\\
&p_{20}(T) = \phi_{L^v_{f2}}(x^*(T)) = 0, \qquad p_{21}(T) = \phi_{H^v_{f2}}(x^*(T)) = 0,,\\
&p_{22}(T) = \phi_{C^v_{f2}}(x^*(T)) = a_1(C^{u*}_{f1}(T) + C^{v*}_{f1}(T) + C^{u*}_{f2}(T) + C^{v*}_{f2}(T) - \mathbb{I});\\
&p_{23}(T) = \phi_{D_{f2}}(x^*(T)) = 0, \qquad p_{24}(T) = \phi_{R_{f2}}(x^*(T)) = 0,\\
&p_{25}(T) = \phi_{S^u_{m1}}(x^*(T)) = 0, \qquad p_{26}(T) = \phi_{I^u_{m1}}(x^*(T)) = 0,\\
&p_{27}(T) = \phi_{S^v_{m1}}(x^*(T)) = 0, \qquad p_{28}(T) = \phi_{I^v_{m1}}(x^*(T)) = 0,\\
&p_{29}(T) = \phi_{R_{m1}}(x^*(T)) = 0, \qquad p_{30}(T) = \phi_{S^u_{m2}}(x^*(T)) = 0,\\
&p_{31}(T) = \phi_{I^u_{m2}}(x^*(T)) = 0, \qquad p_{32}(T) = \phi_{S^v_{m2}}(x^*(T)) = 0,\\
&p_{33}(T) = \phi_{I^v_{m2}}(x^*(T)) = 0, \qquad p_{34}(T) = \phi_{R_{m2}}(x^*(T)) = 0,
\end{aligned} \tag{S10.35}$$

where ϕ_{x_i} is the partial derivative of ϕ with respect to the component x_i.

Appendix 11. The choice about time T

This section is mainly to choose the appropriate T for optimal control by simulating the change of $C(t)$ with time in Case 1 and Case 2.

Case 1: $\phi_f = 0.96$, $k_f = 0.96$, $\sigma^u_{f1} = 0.232$, $\sigma^u_{f2} = 1/3$, $\phi_m = 0$ and $k_m = 0$.

Case 2: $\phi_f = 0.95$, $k_f = 0.95$, $\sigma^u_{f1} = 0.232$, $\sigma^u_{f2} = 1/3$, $\phi_m = 0.95$ and $k_m = 0.96$.

The control strategy corresponding to Case 1 is that all females are vaccinated, males are not vaccinated, and the values of parameters ϕ_f, k_f, σ^u_{f1} and σ^u_{f2} are all their

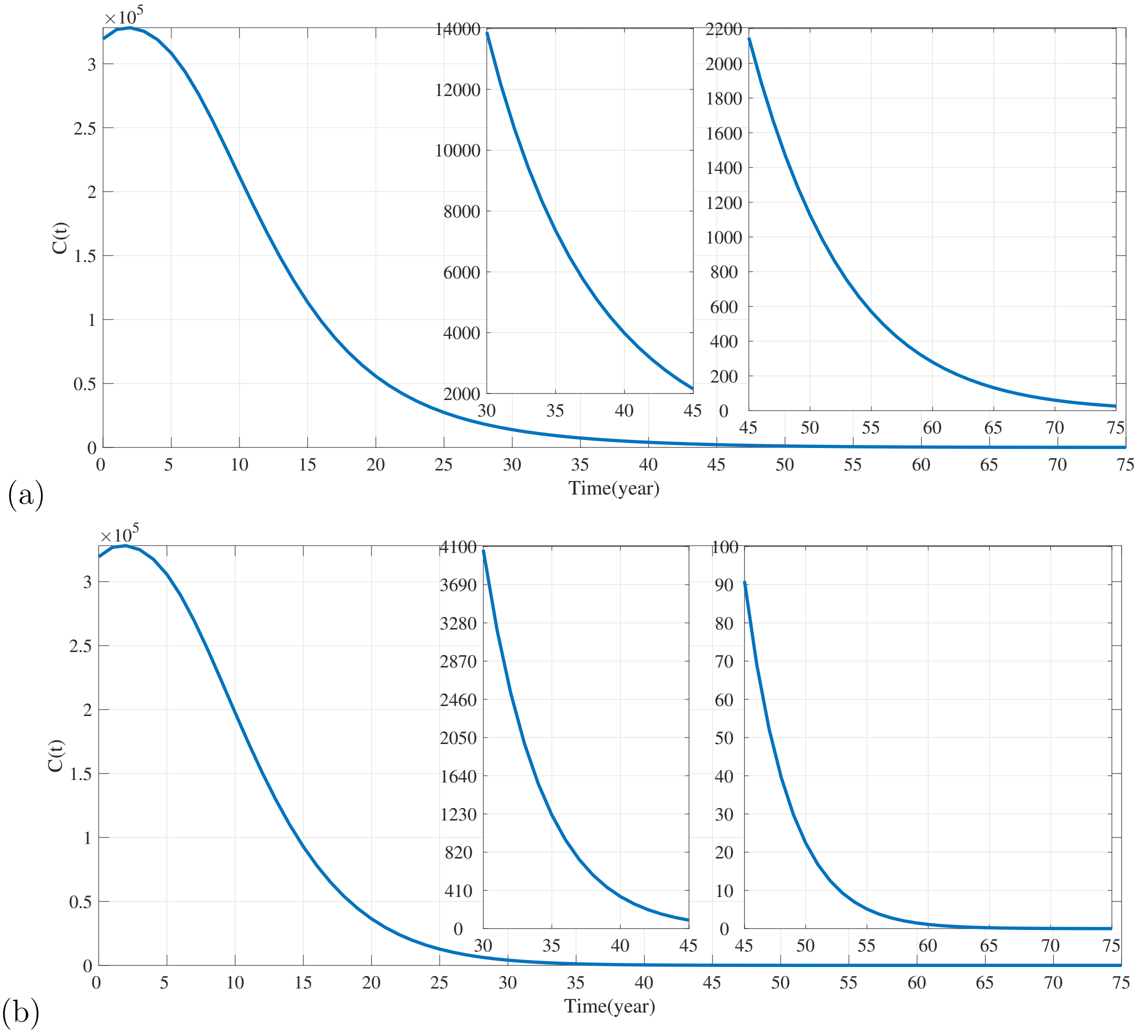

Fig. S11: Variation of $C(t)$ with time for Case 1 and Case 2. (a) Case 1. (b) Case 2.

maximum values. The control strategy corresponding to Case 2 is that both males and females are vaccinated, and the values of parameters ϕ_f, k_f, σ^u_{f1}, σ^u_{f2}, ϕ_m and k_m are all their maximum values. The initial value of the variable whose superscript is not v is the value corresponding to each variable in 2017, and the initial value of the variable whose superscript is v is equal to 0. The values of other parameters can be seen in Tables 3 and 4 (see text). Assume that $d_{f1} = 0.01$ and $d_{f2} = 0.0226$. In addition, when $r^v_{fi} = 1$, $e^v_{fi} = 1$ and $\xi^v_{fi} = 0$ ($i \in \{1, 2\}$), the variables L^v_{fi}, H^v_{fi} and C^v_{fi} are equal to 0. So σ^v_{f1} and σ^v_{f2} are equal to 0. According to the fitting results corresponding to Case 1 and Case 2 (see Fig. S11), $C(51) = 986 < 1000$ for Case 1 and $C(51) = 17 < 20$ for Case 2. Therefore, when the target value $\mathbb{I} = 0$, we can choose a value greater than 50 as the value of T based on the above fitting results.

Appendix 12. Values and sources of parameters related to cost and DALY

The values and sources of the parameters involved in cost and DALY are shown in Table S4.

Table S4: The values and sources of the parameters involved in cost and DALY.

Parameter	Value ($)	Reference	Parameter	Value ($)	Reference
B_1	17.96	[24]	DW_L	0.03	[24]
B_2	26.94	[24]	DW_H	0.1	[24]
E_1	53.265	[24]	DW_C	0.165	[24]
E_2	20.41	[24]	DW_{LD}	0.03	[24]
T_L	214.425	[24]	DW_{HD}	0.0183	[24]
T_H	1503.5925	[24]	DW_{CD}	0.1642	[24]
T_C	9043.615	[24]	a_{f1}	43.24	[19, 5]
\	\	\	a_{f2}	22.51	[19, 5]

References

[1] H. R. Thieme, Mathematics in population biology, Princeton University Press, Princeton, 2003.

[2] P. van den Driessche, J. Watmough, Reproduction numbers and sub-threshold endemic equilibria for compartmental models of disease transmission, Math. Biosci. 180 (2002) 29–48.

[3] H. Smith, Monotone dynamical systems: An introduction to the theory of competitive and cooperative systems, American Mathematical Society, 1995.

[4] H. Smith, X. Q. Zhao, Robust persistence for semidynamical systems, Nonlinear Anal. 47 (2001) 6169–6179.

[5] X. Gu, G. Sun, R. Zheng, S. Zhang, H. Zeng, K. Sun, et al., Incidence and mortality of cervical cancer in China in 2015, J. Natl. Cancer Center 2 (2022) 70–77.

[6] R. Zheng, S. Zhang, H. Zeng, et al., Cancer incidence and mortality in China, 2016, J. Natl. Cancer Center 2 (2022) 1–9.

[7] W. Chen, K. Sun, R. Zheng, et al., Cancer incidence and mortality in China, 2014, Chin. J. Cancer Res. 30 (2018) 1–12.

[8] W. Chen, R. Zheng, S. Zhang, et al., Report of cancer incidence and mortality in China, 2010, Ann. Transl. Med. 2 (2014) 1–25.

[9] W. Chen, R. Zheng, S. Zhang, et al., Report of incidence and mortality in china cancer registries, 2008, Chin. J. Cancer Res. 24 (2012) 171–180.

[10] N. Li, R. Zheng, S. Zhang, et al., An Analysis of Incidence and Mortality of Cervical Cancer in China, 2003 2007, China Cancer 21 (2012) 801–804.

[11] W. Chen, R. Zheng, S. Zhang, et al., Report of Cancer Incidence and Mortality in China, 2013, China Cancer 26 (2017) 1–7.

[12] W. Chen, R. Zheng, H. Zeng, et al., Report of Cancer Incidence and Mortality in China, 2011, China Cancer 24 (2015) 1–10.

[13] W. Chen, R. Zheng, S. Zhang, et al., Report of Cancer Incidence and Mortality in China, 2012, China Cancer 25 (2016) 1–8.

[14] W. Chen, S. Zhang, R. Zheng, et al., Report of Cancer Incidence and Mortality in China, 2009, China Cancer 22 (2013) 2–12.

[15] C. Wei, J. Zhu, Y. Niu, et al., Analysis of the mortality trend of female reproductive system cancer in China from 2004 to 2016, Chin. J. Dis. Control Prev.

23 (2019) 506–511.
[16] X. Li, C. Liu, W. Zhou, et al., Changing Trend of the Incidence and Mortality of Cervical Cancer in China from 2005 to 2015, Act. Med. Univ. Sci. Technol. Huazhong 50 (2021) 325–346.
[17] M. Li, X. Du, M. Lu, et al., Prevalence characteristics of single and multiple HPV infections in women with cervical cancer and precancerous lesions in Beijing, China, J. Med. Virol. 91 (2019) 473–481.
[18] K. Li, R. Yin, D. Wang, Q. Li, Human papillomavirus subtypes distribution among 2309 cervical cancer patients in West China, Oncotarget 8 (2017) 28502–28509.
[19] UN, World Population Prospects 2022, 2022. https://population.un.org/wpp/Download/Standard/Population/.
[20] M. Yuan, H. Wang, R. Duan, et al., Analysis on cancer incidence and mortality attributed to human papillomavirus infection in China, Chin. J. Epidemiol. 43 (2022) 702–708.
[21] J. A. Bogaards, M. Xiridou, V. M. Coupé, et al., Model-based estimation of viral transmissibility and infection-induced resistance from the age-dependent prevalence of infection for 14 high-risk types of human papillomavirus, Am. J. Epidemiol. 171 (2010) 817–825.
[22] D. C. Beachler, G. Jenkins, M. Safaeian, et al., Natural acquired immunity against subsequent genital human papillomavirus infection: a systematic review and meta-analysis, J. Infect. Dis. 213 (2016) 1444–1454.
[23] W. H. Fleming, R. W. Rishel, Deterministic and Stochastic Optimal Control, Springer-Verlag, 1975.
[24] C. Xia, Optimal Strategy and Pathway Towards Cervical Cancer Elimination in China (In Chinese), Peking Union Medical College (2021).
